# Randomized study of rivaroxaban vs. placebo on disease progression and symptoms resolution in high-risk adults with mild COVID-19

**DOI:** 10.1093/cid/ciab813

**Published:** 2021-09-15

**Authors:** Jintanat Ananworanich, Robin Mogg, Michael W Dunne, Mohamed Bassyouni, Consuela Vera David, Erika Gonzalez, Taryn Rogalski-Salter, Heather Shih, Jared Silverman, Jeroen Medema, Penny Heaton

**Affiliations:** 1 Bill & Melinda Gates Medical Research Institute, Cambridge, MA, USA; 2 Science 37, Los Angeles, CA, USA; 3 South Texas Allergy & Asthma Medical Professionals, San Antonio, TX, USA

**Keywords:** coronavirus, SARS-CoV-2, COVID, COVID-19, pneumonia, infection, rivaroxaban

## Abstract

**Background:**

SARS-CoV-2 infection may be associated with a prothrombotic state, predisposing patients for a progressive disease course. We investigated whether rivaroxaban, a direct oral anticoagulant factor Xa inhibitor would reduce COVID-19 progression.

**Methods:**

Adults (N=497) symptomatic with mild COVID-19 and at high-risk for COVID-19 progression based on age, body mass index, or comorbidity were randomized 1:1 to either daily oral rivaroxaban 10 mg (N=246) or placebo-equivalent (N=251) for 21 days and followed to Day 35. Primary endpoints were safety and progression to moderate or severe disease, per the Gates MRI scale. Absolute difference in progression risk was assessed using a stratified Miettinen and Nurminen method.

**Results:**

The study was terminated after 497 of target 600 participants were enrolled due to a pre-specified interim analysis of the first 200 participants which crossed the futility boundary for the primary efficacy endpoint in the Intent to Treat population. Enrollees were 85% aged < 65 years old, 60% female, 27% Hispanic, Black or other minorities and 69% with ≥2 comorbidities. Rivaroxaban was well-tolerated. Disease progression rates were 46/222 (20.7%) in rivaroxaban vs. 44/222 (19.8%) in placebo groups, with a risk difference of -1.0, 95% CI, -6.4 to 8.4; P = 0.78.

**Conclusions:**

Our study did not demonstrate an impact of rivaroxaban on disease progression in high-risk adults with mild COVID-19. There remains a critical public health gap in identifying scalable effective therapies for high-risk people in the outpatient setting to prevent COVID-19 progression.

**Trial Registration Number:** Clinicaltrials.gov, NCT04504032

## Introduction

The global Coronavirus Disease 2019 (COVID-19) pandemic caused by severe acute respiratory syndrome coronavirus 2 (SARS-CoV-2) infection has killed more than 4 million people, overwhelmed healthcare systems, and devastated communities and economies. Most people with SARS-CoV-2 infection are asymptomatic or have mild or moderate symptoms, but 15-20% experience severe symptoms, such as pneumonia and thromboembolic events (TE). Patients with COVID-19 could be predisposed to TE due to excessive inflammation, platelet activation, endothelial dysfunction, and stasis [1, 2]. Pulmonary embolism, deep vein thrombosis (DVT), and other vascular events have been observed in about 17% of COVID-19 patients [3, 4, 5, 6, 7]. Widespread thrombosis and distinct alveolar capillary thrombi were evident in the lungs of patients who died from COVID-19, as compared to those of patients who died from influenza [8]. Even non-severe COVID-19 cases display abnormal coagulation, and infrequently, venous TE (VTE) [4,9,10]. It is possible that immunothrombosis underlies COVID-19 progression and symptoms. Importantly, there are shared risks for COVID-19 progression and thrombosis such as advanced age (≥65 years), presence of comorbidities (e.g., cardiopulmonary disease, diabetes mellitus), and obesity [11].

Intervening early in an outpatient setting before COVID-19 progression is important for low- and middle-income countries (LMICs) where limited healthcare resources exist. Currently the US National Institutes of Health guidelines recommend prophylactic anticoagulants only for hospitalized nonpregnant adults with COVID-19 [12], given the known bleeding risks, and insufficient data, for outpatient prophylaxis.

Rivaroxaban (Xarelto®, Janssen Pharmaceuticals, Inc.) is a licensed, direct oral anticoagulant factor Xa inhibitor for treating and preventing VTE. It has a well characterized safety profile, and the potential for scale-up of global supply. The VTE prophylactic dose of 10mg daily has been given to outpatients after hospital discharge without requirements for blood coagulation monitoring or cold chain storage. We hypothesized that rivaroxaban would reduce COVID-19 progression in people at high risk for disease progression, and evaluated whether rivaroxaban could affect disease resolution or hospitalization.

## Methods

### *Study population and setting*

This Phase 2b, randomized study included male and female participants ≥18 years of age, with a documented positive SARs-CoV-2 polymerase chain reaction (PCR) test within 10 days of screening, and at least one COVID-19 sign or symptom within 7 days of randomization. Eligibility criteria included mild COVID-19 at screening (Supplemental Table 1), and high risk for severe COVID-19 (either ≥65 years of age, diagnosed with a chronic disease requiring daily treatment (such as diabetes, lung disease, heart disease, hypertension or cancer), or self-reported obesity). Participants must not have had any condition associated with bleeding risk (Appendix 1). The study was conducted at 13 outpatient clinics in 7 US states, and at one virtual site (Decentralized Clinical Trial Operating System™, Science37, Culver City, CA) that enrolled participants from 40 states.

The study was approved by the Advarra Institutional Review Board, Columbia MD, United States, which served as the central IRB for all sites, and conformed to the ethical standards of the Declaration of Helsinki.

***Study procedures and outcomes***

The study was conducted virtually at all study sites (virtual site and outpatient clinics) utilizing a telemedicine platform for screening and study visits (Decentralized Clinical Trial Operating System™). Recruitment for the virtual site was done via social media. Participants were randomized 1:1, stratified by site and symptom duration (<6 days vs. ≥6 days), to receive either rivaroxaban (one 10 mg tablet) or placebo-equivalent (multi-vitamin, 1 tablet), orally daily for 21 consecutive days. Participants received a box delivered to their home containing study drug, thermometer, pulse oximeter, nasal swab test kit and labels, and personal protective equipment. There were 12 telemedicine visits (Days 1, 4, 6, 8, 10, 12, 14, 18, 21, 24, 28 and 35). At each visit, adverse events (AEs) and bleeding events based on standardized definitions [13] were recorded per the investigator. Through Day 28, the investigator assessed COVID-19 signs and symptoms per checklist (Appendix 1), Gates MRI scale and WHO scale (Supplemental Table 2 and Supplemental Table 3, respectively), and temperature and oxygen saturation measured by participants. Participants performed nasal swab sample collection on Days 1, 4, 8, 14, 21, and 28. These samples (Viral Transport Medium & Flocked Swab, Ruhof, NY, USA) were picked up by a courier and shipped to the laboratory for PCR testing (Cerba Research, NY, NY, USA). Nasal sample PCR testing was performed sequentially starting with the Day 1 sample, until the last sample was tested, or until viral clearance (two consecutive negative PCR results).

The primary safety endpoint was the frequency of adverse events (AEs) including Grades 3 and 4, resulting in discontinuation, serious AEs and hypersensitivity and major bleeding events through Day 35. The primary efficacy endpoint was the proportion of participants who progressed to moderate or severe disease category (Gates MRI scale 3 or higher), through Day 28. The Gates MRI scale 3 included at least one of these symptoms: shortness of breath, tachypnea (respiratory rate ≥ 20 breathes per minute), or hypoxemia. The Gates MRI scales 4 to 7 encompassed critically ill status to death. The key secondary efficacy endpoint was time to disease resolution. This included symptoms resolution (resolution of all symptoms per checklist), and symptoms resolution with viral clearance. Anosmia and ageusia were collected but not included in the time to symptoms resolution analysis, as they were expected to be long-lasting due to local infection of non-neuronal cells surrounding the olfactory and gustatory neurons. Other secondary endpoints were incidence of hospitalization, and proportion of participants with disease progression, disease resolution, and distributions of the Gates MRI and WHO scale scores, at Days 8, 14, 21 and 28.

### *Sample size and statistical analysis*

The target sample size of 600 was based on an assumed true control rate for progression to moderate or severe category or greater of at least 30%, with 80% power to detect a true study intervention effect to reduce progression rate by 35%. This presumed effect size was guided by that of remdesivir in hospitalized patients [14]. A 2-stage group sequential design was used, with the first interim analysis occurring after the first 200 participants (33%) completed Day 28. A binding futility boundary 1-sided P value of 0.755 was used based on the Hwang-Shih-DeCani spending function with a pre-defined gamma parameter of -4. At interim analysis (03 February 2021), the Independent Data Monitoring Committee (IDMC) recommended that the study be stopped because this futility boundary was crossed.

The final statistical analysis plan was amended to remove a second planned interim analysis after 67% of participants completed Day 28, due to fast enrollment. The amendment also mitigated potential substantial under-estimation of the treatment effect following early study termination, and study drug termination of many of the enrolled participants at the time futility was declared. The final efficacy analysis population included participants who either *1)* completed the study or discontinued the study prior to February 4, 2021, or *2)* completed treatment period or discontinued study drug prior to February 4, 2021.

The intention-to-treat (ITT) population (all randomized participants) was used for the primary analysis after interactions with regulators. Sensitivity analyses were repeated for both *1)* modified ITT (mITT) population (i.e., ITT participants who received at least one dose of study drug and had mild disease at Day 1), and *2)* Per protocol (PP) population (i.e., mITT participants who did not substantially deviate from protocol procedures). The safety analysis population included participants who had at least one dose of study drug.

The absolute difference in the risk of progression of COVID-19 was assessed using a stratified Miettinen and Nurminen method [15] with Cochran-Mantel-Haenszel (CMH) weights [16] stratified by number of days since onset of symptoms at the time of randomization (< 6 or ≥ 6 days). Disease resolution endpoints were assessed using a stratified 1-sided log-rank test. A stratified Cox PH model was used to estimate the hazard ratios and corresponding confidence intervals (CIs) associated with the rate of disease resolution for the study intervention relative to control. No adjustments were made for multiple comparisons. The proportion of participants at key time points with disease progression, disease resolution, AEs, and hospitalizations were summarized by treatment group, with 95% exact CIs and 2-sided P values.

**Results**

Approximately 27,000 adults were prescreened using the Science 37 pre-screening questionnaire (Supplemental Table 4) before entering the screening process. Between August 16, 2020 and February 3, 2021, 538 adults were screened and 497 were enrolled (246 in rivaroxaban and 251 in placebo). At study termination (04 February 2021), 321 (65%) had already completed the study. The numbers of participants in each analysis population were similar between treatment groups (Figure 1). Sixty-four participants discontinued the study and 89 discontinued study drug (similar between groups). In both groups, the most common reasons for discontinuation were COVID-19 worsening before dosing, lost to follow-up, participant's request, and early study termination by the sponsor.

Demographic and baseline characteristics were similar between groups (Table 1). The majority of participants were <65 years old, female, and white. Sixty-nine percent had two or more comorbidities (risk factors for severe COVID-19), the most common being hypertension, obesity, diabetes, and other chronic diseases. Although a positive SARS-CoV-2 PCR, and symptomatic status were required at screening, by Day 1, 18% had a negative SARS-CoV-2 PCR and 0.7% were asymptomatic, and 4% had progressed to moderate or severe disease. During the study, participants had a mean study drug exposure of 18.6 days (vs. 21 days for full exposure) and 82% had ≥75% compliance.

The primary endpoint showed approximately 20% (ITT population) experiencing disease progression, without a difference between groups (Table 2). There were also no differences between groups for the secondary endpoints in this population. The remaining results herein are focused on the mITT population, which is the most clinically relevant (Table 2).

There were no differences in disease progression rates in the mITT population between groups overall, and by subgroups (i.e., by race, ethnicity, sex, duration of symptoms, age, types of comorbidities and BMI) (Supplemental Figure 1). All progressions were to Gates MRI scale 3, except one placebo participant who progressed to Gates MRI scale 4.

There was a higher proportion of asymptomatic participants at Day 28 in the rivaroxaban arm as defined by the Gates MRI scale 1, P = 0.024 (Table 2, Figure 2, and Supplemental Table 5), particularly among those with ≥ 6 days of symptoms at randomization. Similar findings were observed with the WHO ordinal scale (Figure 3).

At Day 28, symptoms that persisted in ≥10% of participants were fatigue (16.9% vs. 22.1%), cough (10.4% vs. 15.8%), nasal congestion (12.6% vs. 12.1%), anosmia (17.5% vs. 22.1%) and ageusia (14.2% vs. 16.3%) in the rivaroxaban and placebo groups, respectively. These symptoms occurred at a similar frequency between groups at baseline. The proportion of participants who had SARS CoV-2 negative test through Day 28 did not differ between groups (Supplemental Table 6).

Nine participants were hospitalized due to COVID-19 pneumonia; however, 3 participants had not begun study intervention when hospitalized. During study treatment, 6 participants were hospitalized due to COVID-19 pneumonia; 2 in the rivaroxaban group (after 2 and 6 days of treatment) and 4 in the placebo group (after 1, 3, 3, and 8 days of treatment). Two additional placebo participants were hospitalized for pancreatitis and malignant hypertension/acute kidney injury, respectively.

The frequencies of any AEs and AEs resulting in study intervention discontinuation were similar between groups. In the rivaroxaban group, slightly more AEs related to study intervention, but fewer SAEs, were reported (Table 3). There were no deaths during the study period. Two SAEs not requiring hospitalization occurred in 2 placebo participants. One experienced DVT diagnosed 13 days after stopping study drug, and 11 days after being discharged for a COVID-19 pneumonia hospitalization. The other participant was diagnosed with recurrent breast cancer after receiving 11 days of placebo.

As shown in Table 3, no participant experienced major bleeding and clinically relevant non-major bleeding was rare, and included 3 participants with hematuria, and 2 with hemorrhoidal bleeding in the rivaroxaban group (2.3%), and 1 participant with rectal bleeding and one with blood in stool in the placebo group (0.9%). One rivaroxaban participant had minimal bilateral subconjunctival hemorrhage. All participants discontinued study drug due to the bleeding events except for 1 participant with hematuria reported 13 days after completing rivaroxaban.

**Discussion**

In adults with mild COVID-19 at high risk for severe disease, administration of rivaroxaban for 21 days did not reduce progression to moderate or severe disease, compared to placebo. We did observe a higher proportion of asymptomatic participants at Day 28, after rivaroxaban treatment. The incidence of hospitalization was low in both groups, but more cases of COVID-19 pneumonia and vascular events were observed in placebo compared to rivaroxaban recipients. Rivaroxaban was well-tolerated with few AEs and minor bleeding events related to the drug.

We enrolled high-risk adults who presented with mild disease during the first week of their illness, to intervene, as the disease is thought to transition from the active viral replication phase to the inflammatory phase, and before disease progressed [17]. Rivaroxaban does not possess direct antiviral or anti-inflammatory properties, but it may mitigate the consequences of SARS- CoV-2 replication and inflammation. We posited that rivaroxaban could prevent COVID-19- associated VTE and microthrombi in the lungs and other organs, that have been theorized to cause COVID-19 symptoms and progression. This could potentially explain the observation that more participants achieved asymptomatic status. It appears that the observed differences between groups occurred after Day 14 in the study, and in participants who had longer time from onset of symptoms to randomization. We postulate that the persistence of symptoms after the second week of illness may be the result of microthrombi against which rivaroxaban is exerting a positive effect. It is possible that administering rivaroxaban during mild disease and within the first 2 weeks of illness was too early, as a major shift to proinflammatory and coagulopathy state occurs when disease transitions from mild to moderate [18]. We did not collect blood for biomarkers such as D-dimer in this community-based study. However, such examination should be considered in future studies to aid in potentially identifying a population that would most benefit from prophylactic anticoagulant. The daily dose of 10mg rivaroxaban for 21 days may have been insufficient. The selection was based on the indication for VTE prophylaxis in acutely ill patients (10mg daily for 31-39 days after hospital discharge), and a safety profile suitable for remote monitoring in our study. Fatigue, cough, anosmia, and ageusia appeared less frequently in the rivaroxaban group at Day 28. Whether rivaroxaban could benefit long-term COVID-19 symptoms is unknown and should be further investigated.

Our study does not rule out a possible effect of rivaroxaban on disease progression and hospitalization. The sample size was too small to detect an effect size lower than 35% for disease progression, and hospitalizations were rare. Our primary endpoint of disease progression was based on the Gates MRI scale, that, like other instruments, was not validated at the time of use. We established the scale to provide more granular definitions for mild disease. It may have inaccurately captured disease progression due to the subjective nature of shortness of breath that defined the majority of the progression cases. Moreover, we may not have enrolled people with the highest risks for severe COVID-19, as reflected by the low representations of the elderly, minorities, and people with significant comorbidities. The recruitment via social media and the virtual trial design posed challenges in engaging these groups. The need to ship supplies to participants' homes delayed time to first dose of study drug by about 2 days, which may have contributed to a proportion of participants experiencing negative SARS CoV-2 PCR and/or COVID-19 progression at Day 1.

The NIH COVID-19 guidelines did not recommend anticoagulants for non-hospitalized patients for the prevention of VTE or arterial thrombosis unless the patient has other indications for the therapy or is participating in a clinical trial [12]. A large nationwide registry during COVID-19 pandemic in Sweden showed no association between outpatient DOAC use and risk of hospitalization for COVID-19 [19]. As of July 2020, there were at least 75 randomized clinical trials of antithrombotic therapy in COVID-19, including at least 7 studies of prophylactic DOAC in non-hospitalized patients, which vary in study populations and designs, type of drugs and their dosing [5]. Timing of administration and dose level of anti-thrombotics appear critical. Therapeutic rivaroxaban or enoxaparin given to hospitalized patients may not significantly improve outcomes and could increase bleeding events [20, 21].

There is limited capacity to treat severe disease in LMICs [22]. It is of high public health interest to identify mild disease treatments with greater potential for scale-up (e.g., oral administration, stable at room temperature, and low cost), but none has gained regulatory approval. Trial results of oral drugs such as hydroxychloroquine have been disappointing [23]. Modest benefits were observed for colchicine in reducing the risk of death and hospitalization [24] and inhaled budesonide in reducing urgent care visits and hastening symptoms resolution [25]. Monoclonal antibody (mAbs) treatments for people with mild to moderate COVID-19 and at risk for severe disease have been granted FDA Emergency Use Authorization, including bamlanivimab/etesevimab and casirivimab/imdevimab combinations. Their requirement for an intravenous or injectable administration during the first few days of symptoms poses an uptake challenge. The relatively high cost, and cold chain requirements render mAbs unlikely to be readily available in LMICs.

Our study did not demonstrate an impact of rivaroxaban on disease progression in adults with mild COVID-19 and at high risk for severe COVID-19. The potential effect of rivaroxaban on symptoms resolution should be confirmed in larger studies. A number of trials are already ongoing to evaluate the effects of rivaroxaban [26] and apixaban [27] on thromboembolic events, hospitalizations, and deaths. There remains a critical global public health gap in identifying effective therapies that could be scaled-up for at-risk people in the outpatient setting to accelerate disease resolution, and to prevent disease progression, hospitalizations, and deaths.

**NOTES**

**Acknowledgements**

The authors are grateful to the study participants for their contribution to this study. We would like to thank the study investigators Shari DeSilva, Shirley Nagel, Neal Warshoff for their contribution to this study. We thank the members of the Gates MRI RESPOND-1 Study Group: Russ Weiner, Jane Kramer, Ram Narayanan, Chantal Sarmanoukian, Micha Levi, Cari Tsinovoi, Maryann Livolsi, Sally Jennings, Alan Gibson, Alexander Schmidt, Charles Wells, Jim Alexander, Joel Alvarez, Karen Ward, Melissa Tice, Apurve Dhariwal, Laura Gibson, Lynn Agostinelli, Caroline Alix, and Paul Lithotomos. In addition we would like to thank our partners the contract research organization (CRO), PPD, particularly Irak Leon and Paul Carter for their contributions, along with many other PPD's team members, and Kaila Bode-Shaw from Science37 and the rest of Science37 team members.

We thank Debbie Daehnick for providing medical writing support, which was funded by the Gates MRI in accordance with Good Publication Practice (GPP3) guidelines (http://www.ismpp.org/gpp3). We also thank the regulatory authorities reviewing this study, US FDA and MHRA, for their tireless work during the pandemic to provide thorough and expedient reviews of clinical trial applications.

**Funding:**

This work was supported by a grant from Gates Philanthropy Partnerships and the Bill & Melinda Gates Foundation.

**Conflict of Interest**

MD reports support from Bill and Melinda Gates Medical Research Institute (they are an employee of the Gates MRI which funded this project.)

EG reports support from AstraZeneca, Genentech, Regeneron, TEVA, and DBV; reports consulting fees from P&T Ascellahealth; payment/honoraria from TEVA (Digihaler) and Genentech (Omalizumab); reports serving on Regeneron Pediatric Asthma Advisory Board and ACAAI Patient Advocacy Board. TRS, RM and JAS report employment with Gates MRI. TRS reports being a participant/presenter for Gates MRI Scientific Advisory Board – COVID.

**Table 1: Baseline Characteristics (ITT population)**

| Characteristic at Day 1 | Rivaroxaban (N=222) n (%) | Placebo (N=222) n (%) | Total (N=444) n (%) |
|---|---|---|---|
| **Median age, years (min - max)** | 49 (20-83) | 49 (18-75) | 49 (18-83) |
| <65 years | 187 (84.2) | 192 (86.5) | 379 (85.4) |
| ≥65 years | 35 (15.8) | 30 (13.5) | 65 (14.6) |
| **Male** | 96 (43.2) | 81 (36.5) | 177 (39.9) |
| **Female** | 126 (56.8) | 141 (63.5) | 267 (60.1) |
| **Race** | | | |
| American Indian or Alaska Native | 1 (0.5) | 2 (0.9) | 3 (0.7) |
| Asian | 0 (0.0) | 1 (0.5) | 1 (0.2) |
| Black or African American | 15 (6.8) | 15 (6.8) | 30 (6.8) |
| White | 198 (89.2) | 197 (88.7) | 395 (89.0) |
| Mixed race | 0 (0.0) | 0 (0.0) | 0 (0.0) |
| Other | 7 (3.2) | 7 (3.2) | 14 (3.2) |
| Unknown | 1 (0.5) | 0 (0.0) | 1 (0.2) |
| **Ethnicity** | | | |
| Hispanic or Latino | 48 (21.6) | 40 (18.0) | 88 (19.8) |
| Not Hispanic or Latino | 174 (78.4) | 178 (80.2) | 352 (79.3) |
| Not Reported | 0 (0.0) | 4 (1.8) | 4 (0.9) |
| Unknown | 0 (0.0) | 0 (0.0) | 0 (0.0) |

| | | | |
|---|---|---|---|
| **Body Mass Index** | | | |
| Median | 35.2<br>(16.9 to 68.7) | 33.2<br>(18.8 to 66.4) | 34.7<br>(16.9 to 68.7) |
| <25 kg/m^2 | 17 (7.7) | 29 (13.1) | 46 (10.4) |
| 25 to <35 kg/m^2 | 88 (39.6) | 96 (43.2) | 184 (41.4) |
| ≥35 kg/m^2 | 117 (52.7) | 97 (43.7) | 214 (48.2) |
| **Co-morbidity Factors** | | | |
| Age ≥65 years | 35 (15.8) | 30 (13.5) | 65 (14.6) |
| Presence of chronic pulmonary disease, COPD, pulmonary hypertension | 14 (6.3) | 13 (5.9) | 27 (6.1) |
| Diabetes mellitus (type 1 or type 2) requiring medication or insulin | 57 (25.7) | 66 (29.7) | 123 (27.7) |
| Hypertension, requiring at least one oral medication for intervention | 106 (47.7) | 124 (55.9) | 230 (51.8) |
| Immunocompromised status due to disease | 4 (1.8) | 11 (5.0) | 15 (3.4) |
| Participants with 2 or more co-morbidity factors | 154 (69.4) | 151 (68.0) | 305 (68.7) |
| **Median Duration of Symptoms, days** | | | |
| Median (min - max) | 8.0 (2-14) | 8.0 (2-12) | 8.0 (2-14) |
| < 6 days | 24 (12.2) | 35 (17.4) | 59 (14.9) |
| ≥ 6 days | 172 (87.8) | 166 (82.6) | 338 (85.1) |
| **Positive SARS CoV-2 PCR test** | 158 (81.4) | 163 (82.3) | 321 (81.9) |

| | | | |
|---|---|---|---|
| **Gates MRI ordinal scale** | | | |
| 1 (Asymptomatic) | 2 (1.0) | 1 (0.5) | 3 (0.7) |
| 2 (Mild disease) | 193 (93.2) | 200 (97.6) | 393 (95.4) |
| 3 (Moderate or severe disease) | 12 (5.8) | 4 (2.0) | 16 (3.9) |
| **WHO ordinal scale** | | | |
| 0 (Uninfected, no viral RNA detected) | 2 (1.0) | 0 (0) | 2 (0.5) |
| 1 (Asymptomatic; viral RNA detected) | 0 (0) | 1 (0.5) | 1 (0.2) |
| 2 (Symptomatic; independent) | 204 (98.6) | 204 (99.5) | 408 (99.0) |
| 3 (Symptomatic; assistance needed) | 1 (0.5) | 0 (0) | 1 (0.2) |

**Table 2: Proportion of Participants with Disease Progression, Symptoms Resolution, No Symptoms and Hospitalization Through Day 28**

| | ITT Population | | | mITT Population | | | Per Protocol Population | | |
|---|---|---|---|---|---|---|---|---|---|
| | Rivaroxaban (N=222) | Placebo (N=222) | Rivaroxaban vs. Placebo Risk difference [95% CI] | Rivaroxaban (N=192) | Placebo (N=199) | Rivaroxaban vs. Placebo Risk difference [95% CI] | Rivaroxaban (N=172) | Placebo (N=174) | Rivaroxaban vs. Placebo Risk difference [95% CI] |
| **Proportion with disease progression, n (%) [CI]** | 46 (20.7) [15.8, 26.4] | 44 (19.8) [15.0, 25.5] | 1.0 [-6.4, 8.4] P = 0.78 | 18 (9.4) [5.8, 14.1] | 23 (11.6) [7.7, 16.6] | -2.2 [-8.4, 4.0] P = 0.47 | 17 (9.9) [6.1, 15.0] | 16 (9.2) [5.5, 14.2] | 0.6 [-5.7, 7.1] P = 0.85 |
| **Proportion who met protocol-defined symptoms resolution[a], n (%) [CI]** | 132 (59.5) [52.9, 65.8] | 122 (55.0) [48.4, 61.4] | 4.5 [-4.7,13.6] P = 0.34 | 130 (67.7) [60.8, 74.0] | 119 (59.8) [52.9, 66.4] | 7.9 [-1.7, 17.3] P = 0.106 | 118 (68.6) [61.4, 75.2] | 108 (62.1) [54.7, 69.1] | 6.5 [-3.6,16.4] P = 0.21 |
| **Proportion of asymptomatic participants[b] (Gates MRI Scale 1), n (%) [CI]** | 125 (56.3) [49.7, 62.7] | 108 (48.6) [42.1,55.2] | 7.6 [-1.7, 16.8] P = 0.11 | 123 (64.1) [57.1, 70.6] | 105(52.8) [45.8, 59.6] | 11.3 [1.5, 20.9] P = 0.02 | 112 (65.1) [57.8, 72.0] | 97 (55.7) [48.3, 63.0] | 9.4 [-1.0, 19.5] P = 0.08 |
| **Proportion with hospitalization, n (%) [CI]** | 3 (1.4) [0.3, 3.6] | 7 (3.2) [1.4, 6.1] | 0.43 [0.11, 1.65] P = 0.21 | 2 (1.0) [0.2, 3.4] | 5 (2.5) [0.9, 5,5] | 0.41 [0.08, 2.07] P = 0.27 | 2 (1.2) [0.2, 3.8] | 2 (1.1) [0.2, 3.7] | 0.99 [0.14, 6.85] P = 0.99 |

CI=confidence interval; P = 2-sided P values.

Risk difference is shown for all parameters except for proportion with hospitalization, for which relative risk is shown.

Risk difference and relative risks are adjusted for the randomization stratification factor of days since onset of symptoms at time of randomization (<6 days vs. ≥6 days).

[a] Symptoms resolution is defined as disappearance or a return to baseline for all COVID-19-related symptoms, except for anosmia and ageusia, through Day 28.

[b]Asymptomatic is defined as lack of symptoms related to COVID-19 at Day 28.

## Table 3: Summary of Adverse Events

| | Rivaroxaban (N=219) | Placebo (N=230) |
|---|---|---|
| **Type of event** | **n (%)** | **n (%)** |
| **Any AEs** | 35 (16.0) | 36 (15.7) |
| **Related AEs** | 6 (2.7) | 1 (0.4) |
| **AEs resulting in discontinuation of study intervention** | 4 (1.8) | 5 (2.2) |
| **Deaths** | 0 (0.0) | 0 (0.0) |
| **Grade 4 or Grade 5 AE** | 0 (0.0) | 0 (0.0) |
| **Serious AEs**[a] | 2 (0.9) | 7 (3.0) |
| **Severe hypersensitivity** | 0 (0.0) | 0 (0.0) |
| **Major bleeding** | 0 (0.0) | 0 (0.0) |
| **Clinically relevant non-major bleeding**[b] | 5 (2.3) | 2 (0.9) |

AE=Adverse event, SAE=serious AE

N= number of participants

A participant with multiple adverse events with different grades was counted only once under the highest grade for the n and % values.

[a] None of the AEs were determined to be related to the study drug, per investigator assessment

[b] Not clinically significant (P=0.27)

**Figure Titles and Legends/Notes**

**Figure 1: Participant Flow Diagram**

No legend or footnotes

**Figure 2: Proportion of Participants Within Each Gates MRI Scale at Days 1, 8, 14, 21, and 28 (mITT Population)**

Footnotes: Gates MRI Ordinal Scale: 1 = Asymptomatic/symptoms similar to pre-COVID status, 2 = Mild, 3 = Moderate or Severe, 4 = Critically ill, 5 = Critically ill with invasive mechanical ventilation or extrapulmonary complication, 6 = Critically ill with extra corporeal membrane oxygenation, 7 = Death

NA=Participants in mITT Population who had no available Gates MRI scale value at post-baseline assessments.

**Figure 3: Proportion of Participants Within Each WHO Scale at Days 1, 8, 14, 21, and 28 (mITT Population)**

Footnotes: WHO Ordinal Scale: 0 = Uninfected; 1 = Asymptomatic; viral RNA detected, 2 = Symptomatic; independent, 3 = Symptomatic; assistance needed, 4 = Hospitalized; no oxygen therapy, 5 = Hospitalized; oxygen by mask or nasal prongs, 6 = Hospitalized; oxygen by NIV or high flow, 7 = Intubation and mechanical ventilation, $pO_2/FiO_2 \geq 150$ or $SpO_2/FiO_2 \geq 200$, 8 = Mechanical ventilation $pO_2/FiO_2 < 150$ ($SpO_2/FiO_2 < 200$) or vasopressors, 9 = Mechanical ventilation $pO_2/FiO_2 < 150$ and vasopressors, dialysis, or ECMO, 10 = Dead.

NA=Participants in mITT Population who have no available WHO scale value at post-baseline assessments

Figure 1

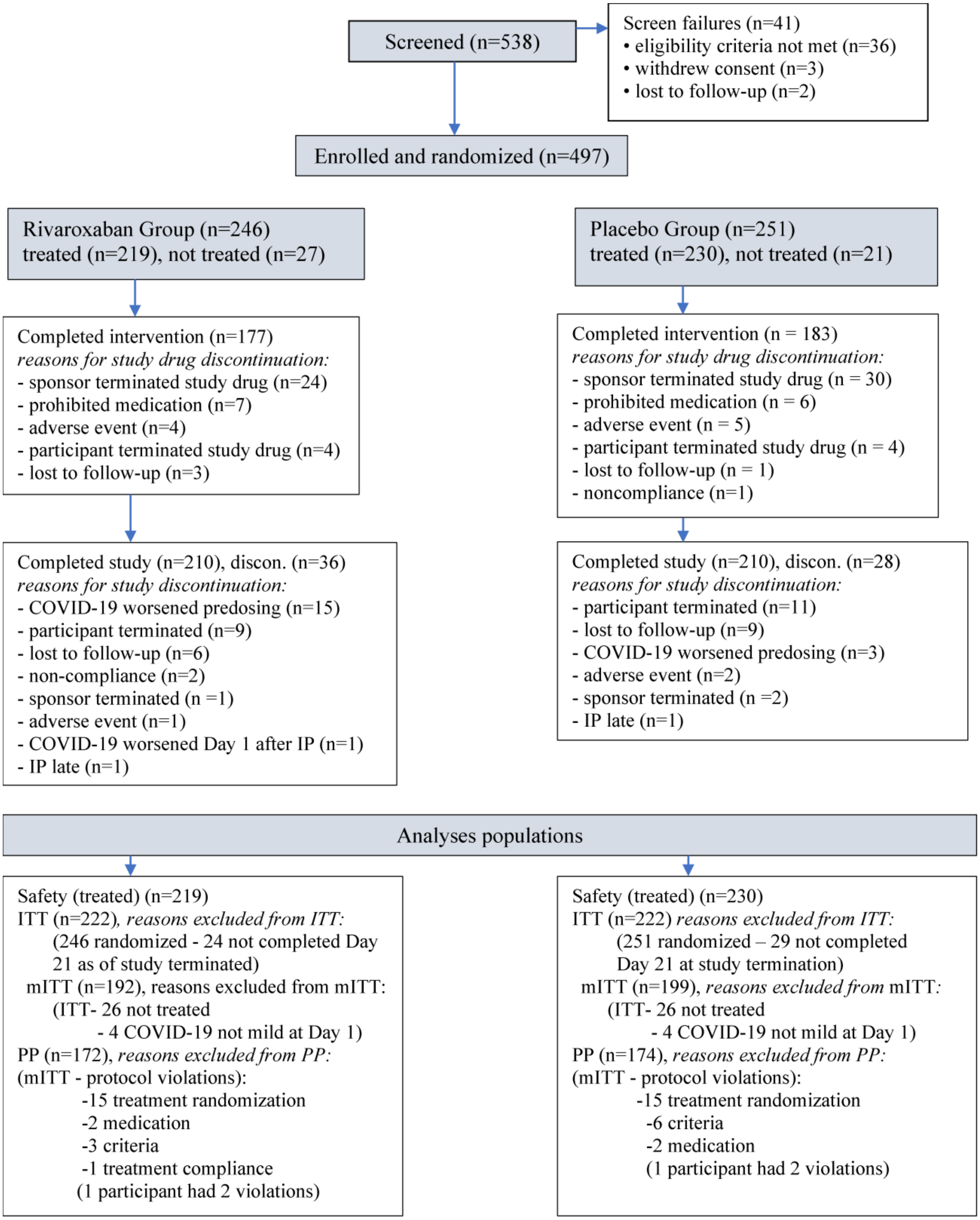

Screened (n=538)
Screen failures (n=41)
• eligibility criteria not met (n=36)
• withdrew consent (n=3)
• lost to follow-up (n=2)
Enrolled and randomized (n=497)
Rivaroxaban Group (n=246)
treated (n=219), not treated (n=27)
Placebo Group (n=251)
treated (n=230), not treated (n=21)
Completed intervention (n=177)
reasons for study drug discontinuation:
- sponsor terminated study drug (n=24)
- prohibited medication (n=7)
- adverse event (n=4)
- participant terminated study drug (n=4)
- lost to follow-up (n=3)
Completed intervention (n = 183)
reasons for study drug discontinuation:
- sponsor terminated study drug (n = 30)
- prohibited medication (n = 6)
- adverse event (n = 5)
- participant terminated study drug (n = 4)
- lost to follow-up (n = 1)
- noncompliance (n=1)
Completed study (n=210), discon. (n=36)
reasons for study discontinuation:
- COVID-19 worsened predosing (n=15)
- participant terminated (n=9)
- lost to follow-up (n=6)
- non-compliance (n=2)
- sponsor terminated (n =1)
- adverse event (n=1)
- COVID-19 worsened Day 1 after IP (n=1)
- IP late (n=1)
Completed study (n=210), discon. (n=28)
reasons for study discontinuation:
- participant terminated (n=11)
- lost to follow-up (n=9)
- COVID-19 worsened predosing (n=3)
- adverse event (n=2)
- sponsor terminated (n =2)
- IP late (n=1)
Analyses populations
Safety (treated) (n=219)
ITT (n=222), reasons excluded from ITT:
(246 randomized - 24 not completed Day 21 as of study terminated)
mITT (n=192), reasons excluded from mITT:
(ITT- 26 not treated
- 4 COVID-19 not mild at Day 1)
PP (n=172), reasons excluded from PP:
(mITT - protocol violations):
-15 treatment randomization
-2 medication
-3 criteria
-1 treatment compliance
(1 participant had 2 violations)
Safety (treated) (n=230)
ITT (n=222) reasons excluded from ITT:
(251 randomized – 29 not completed Day 21 at study termination)
mITT (n=199), reasons excluded from mITT:
(ITT- 26 not treated
- 4 COVID-19 not mild at Day 1)
PP (n=174), reasons excluded from PP:
(mITT - protocol violations):
-15 treatment randomization
-6 criteria
-2 medication
(1 participant had 2 violations)

Figure 2

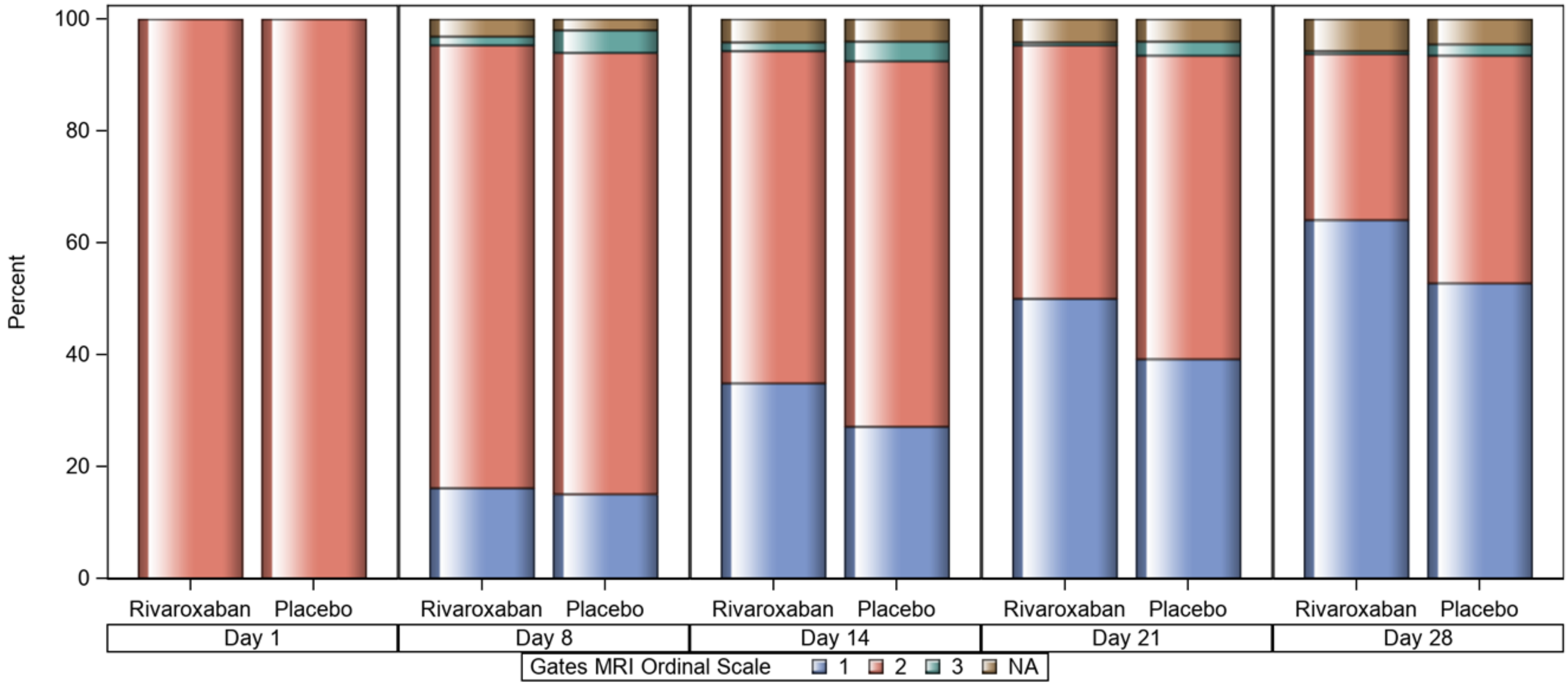

100
80
60
40
20
0
Percent
Rivaroxaban
Placebo
Rivaroxaban
Placebo
Rivaroxaban
Placebo
Rivaroxaban
Placebo
Rivaroxaban
Placebo
Day 1
Day 8
Day 14
Day 21
Day 28
Gates MRI Ordinal Scale 1 2 3 NA

Figure 3

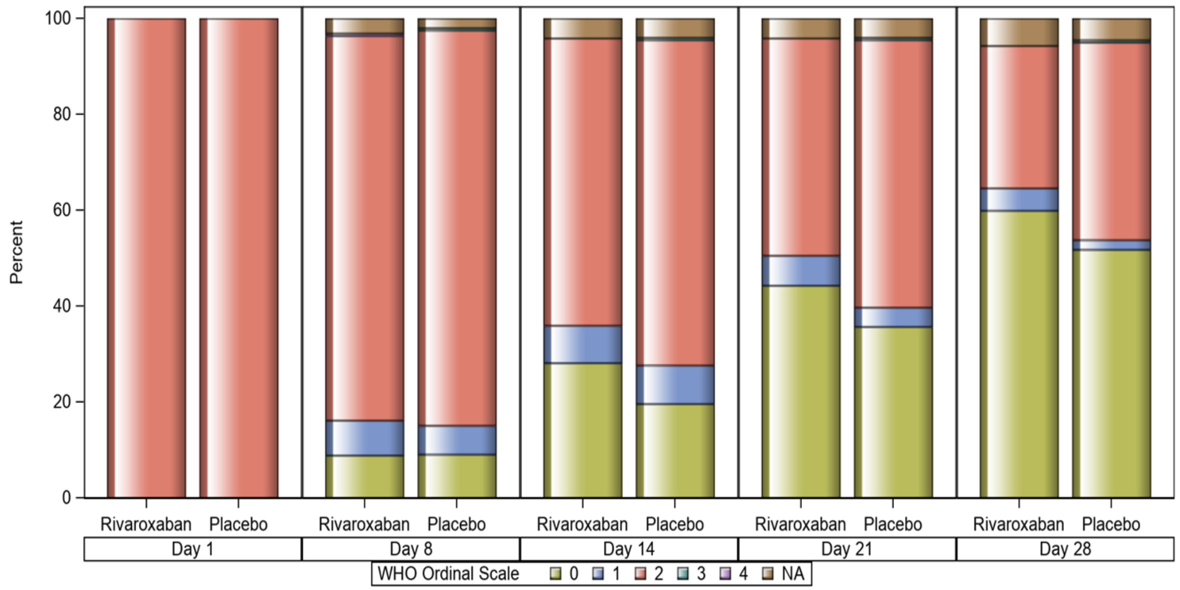

Percent
100
80
60
40
20
0
Rivaroxaban
Placebo
Rivaroxaban
Placebo
Rivaroxaban
Placebo
Rivaroxaban
Placebo
Rivaroxaban
Placebo
Day 1
Day 8
Day 14
Day 21
Day 28
WHO Ordinal Scale 0 1 2 3 4 NA

## Supplementary Material

ciab813_suppl_Supplementary_Data

ciab813_suppl_Supplementary_Figure_S1

ciab813_suppl_Supplementary_Table_S1

ciab813_suppl_Supplementary_Table_S2

ciab813_suppl_Supplementary_Table_S3

ciab813_suppl_Supplementary_Table_S4

ciab813_suppl_Supplementary_Table_S5

ciab813_suppl_Supplementary_Table_S6

