# Randomized Study of Rivaroxaban vs Placebo on Disease Progression and Symptoms Resolution in High-Risk Adults With Mild Coronavirus Disease 2019

**DOI:** 10.1093/cid/ciab813

**Published:** 2021-09-15

**Authors:** Jintanat Ananworanich, Robin Mogg, Michael W Dunne, Mohamed Bassyouni, Consuela Vera David, Erika Gonzalez, Taryn Rogalski-Salter, Heather Shih, Jared Silverman, Jeroen Medema, Penny Heaton

**Affiliations:** Department of Clinical Development, Bill & Melinda Gates Medical Research Institute, Cambridge, Massachusetts, USA; Department of Clinical Development, Bill & Melinda Gates Medical Research Institute, Cambridge, Massachusetts, USA; Department of Clinical Development, Bill & Melinda Gates Medical Research Institute, Cambridge, Massachusetts, USA; Department of Clinical Development, Bill & Melinda Gates Medical Research Institute, Cambridge, Massachusetts, USA; Department of Allergy, Asthma and Clinical Research, Science 37, Los Angeles, California, USA; Department of Medical Affairs, South Texas Allergy & Asthma Medical Professionals, San Antonio, Texas, USA; Department of Clinical Development, Bill & Melinda Gates Medical Research Institute, Cambridge, Massachusetts, USA; Department of Clinical Development, Bill & Melinda Gates Medical Research Institute, Cambridge, Massachusetts, USA; Department of Clinical Development, Bill & Melinda Gates Medical Research Institute, Cambridge, Massachusetts, USA; Department of Clinical Development, Bill & Melinda Gates Medical Research Institute, Cambridge, Massachusetts, USA; Department of Clinical Development, Bill & Melinda Gates Medical Research Institute, Cambridge, Massachusetts, USA

**Keywords:** SARS-CoV-2, COVID-19, pneumonia, infection, rivaroxaban

## Abstract

**Background:**

Severe acute respiratory syndrome coronavirus 2 infection may be associated with a prothrombotic state, predisposing patients for a progressive disease course. We investigated whether rivaroxaban, a direct oral anticoagulant factor Xa inhibitor, would reduce coronavirus disease 2019 (COVID-19) progression.

**Methods:**

Adults (N = 497) with mild COVID-19 symptoms and at high risk for COVID-19 progression based on age, body mass index, or comorbidity were randomized 1:1 to either daily oral rivaroxaban 10 mg (N = 246) or placebo equivalent (N = 251) for 21 days and followed to day 35. Primary end points were safety and progression. Absolute difference in progression risk was assessed using a stratified Miettinen and Nurminen method.

**Results:**

The study was terminated after 497 of the target 600 participants were enrolled due to a prespecified interim analysis of the first 200 participants that crossed the futility boundary for the primary efficacy end point in the intent-to-treat population. Enrollees were 85% aged <65 years; 60% female; 27% Hispanic, Black, or other minorities; and 69% with ≥2 comorbidities. Rivaroxaban was well tolerated. Disease progression rates were 46 of 222 (20.7%) in rivaroxaban vs 44 of 222 (19.8%) in placebo groups, with a risk difference of –1.0 (95% confidence interval, −6.4 to 8.4; *P* = .78).

**Conclusions:**

We did not demonstrate an impact of rivaroxaban on disease progression in high-risk adults with mild COVID-19. There remains a critical public health gap in identifying scalable effective therapies for high-risk people in the outpatient setting to prevent COVID-19 progression.

## Teaser

Rivaroxaban did not impact progression from mild to moderate or severe coronavirus disease 2019 (COVID-19) in adults at high risk for severe COVID-19.

Clinical Trials Registration. NCT04504032.

The global coronavirus disease 2019 (COVID-19) pandemic caused by severe acute respiratory syndrome coronavirus 2 (SARS-CoV-2) infection has killed more than 4 million people, overwhelmed healthcare systems, and devastated communities and economies. Most people with SARS-CoV-2 infection are asymptomatic or have mild or moderate symptoms, but 15%–20% experience severe symptoms, such as pneumonia and thromboembolic events (TEs). Patients with COVID-19 could be predisposed to TEs due to excessive inflammation, platelet activation, endothelial dysfunction, and stasis [1, 2]. Pulmonary embolism, deep vein thrombosis (DVT), and other vascular events have been observed in about 17% of COVID-19 patients [3–7]. Widespread thrombosis and distinct alveolar capillary thrombi were evident in the lungs of patients who died from COVID-19 compared with those patients who died from influenza [8]. Even nonsevere COVID-19 cases display abnormal coagulation and, infrequently, venous TE (VTE) [4, 9, 10]. It is possible that immunothrombosis underlies COVID-19 progression and symptoms. Importantly, there are shared risks for COVID-19 progression and thrombosis such as advanced age (≥65 years), presence of comorbidities (eg, cardiopulmonary disease, diabetes mellitus), and obesity [11].

Intervening early in an outpatient setting before COVID-19 progression is important for low- and middle-income countries (LMICs) where limited healthcare resources exist. Currently, the US National Institutes of Health guidelines recommend prophylactic anticoagulants only for hospitalized nonpregnant adults with COVID-19 [12], given the known bleeding risks and insufficient data for outpatient prophylaxis.

Rivaroxaban (Xarelto, Janssen Pharmaceuticals, Inc) is a licensed, direct oral anticoagulant factor Xa inhibitor for treating and preventing VTE. It has a well-characterized safety profile and the potential for scale-up of global supply. The VTE prophylactic dose of 10 mg daily has been given to outpatients after hospital discharge without requirements for blood coagulation monitoring or cold chain storage. We hypothesized that rivaroxaban would reduce COVID-19 progression in people at high risk for disease progression and determined whether rivaroxaban could affect disease resolution or hospitalization.

## METHODS

### Study Population and Setting

This phase 2b, randomized study included male and female participants aged ≥18 years with a documented positive SARs-CoV-2 polymerase chain reaction (PCR) test within 10 days of screening and at least 1 COVID-19 sign or symptom within 7 days of randomization. Eligibility criteria included mild COVID-19 at screening (Supplementary Table 1) and high risk for severe COVID-19 (either aged ≥65 years and diagnosed with a chronic disease that requires daily treatment such as diabetes, lung disease, heart disease, hypertension, or cancer or self-reported obesity). Participants must not have had any condition associated with bleeding risk (Supplementary Materials 1). The study was conducted at 13 outpatient clinics in 7 US states and at 1 virtual site (Decentralized Clinical Trial Operating System, Science37, Culver City, California) that enrolled participants from 40 states.

The Advarra Institutional Review Board (Columbia, Maryland), which served as the central institutional review board for all sites, approved the study. The study conforms to the ethical standards of the Declaration of Helsinki.

### Study Procedures and Outcomes

The study was conducted virtually at all study sites (virtual site and outpatient clinics) using a telemedicine platform for screening and study visits (Decentralized Clinical Trial Operating System). Recruitment for the virtual site was done via social media. Participants were randomized 1:1, stratified by site and symptom duration (<6 days vs ≥6 days), to receive either rivaroxaban (one 10-mg tablet) or placebo equivalent (multivitamin, 1 tablet) orally daily for 21 consecutive days. Participants received a box delivered to their home that contained the study drug (ie, either rivaroxaban or placebo), thermometer, pulse oximeter, nasal swab test kit and labels, and personal protective equipment. There were 12 telemedicine visits (days 1, 4, 6, 8, 10, 12, 14, 18, 21, 24, 28, and 35). At each visit, adverse events (AEs) and bleeding events based on standardized definitions [13] were recorded per the investigator. Through day 28, the investigator assessed COVID-19 signs and symptoms per checklist (Supplementary Materials 1), Gates Medical Research Institute (MRI) scale, and World Health Organization (WHO) scale (Supplementary Tables 2 and 3, respectively) as well as temperature and oxygen saturation measured by participants. Participants performed nasal swab sample collection on days 1, 4, 8, 14, 21, and 28. These samples (Viral Transport Medium & Flocked Swab, Ruhof, NY) were picked up by a courier and shipped to the laboratory for PCR testing (Cerba Research, New York, NY). Nasal sample PCR testing was performed sequentially starting with the day 1 sample until the last sample was tested or until viral clearance (2 consecutive negative PCR results).

The primary safety end point was the frequency of AEs, including grades 3 and 4, resulting in discontinuation, serious AEs and hypersensitivity, and major bleeding events through day 35. The primary efficacy end point was the proportion of participants who progressed to a moderate or severe disease category (Gates MRI scale 3 or higher) through day 28. The Gates MRI scale 3 included at least 1 of the following symptoms: shortness of breath, tachypnea (respiratory rate ≥20 breathes per minute), or hypoxemia. The Gates MRI scales 4 to 7 encompassed critically ill status to death. The key secondary efficacy end point was time to disease resolution. This included symptoms resolution (resolution of all symptoms per checklist) and symptoms resolution with viral clearance. Data regarding anosmia and ageusia were collected but not included in the time to symptoms resolution analysis, as they were expected to be long-lasting due to local infection of nonneuronal cells surrounding the olfactory and gustatory neurons. Other secondary end points were incidence of hospitalization and proportion of participants with disease progression, disease resolution, and distributions of the Gates MRI and WHO scale scores at days 8, 14, 21, and 28.

### Sample Size and Statistical Analyses

The target sample size of 600 was based on an assumed true control rate for progression to moderate or severe category or greater of at least 30%, with 80% power to detect a true study intervention effect to reduce the progression rate by 35%. This presumed effect size was guided by that of remdesivir in hospitalized patients [14]. A 2-stage group sequential design was used, with the first interim analysis occurring after the first 200 participants (33%) completed day 28. A binding futility boundary 1-sided *P* value of .755 was used based on the Hwang-Shih-DeCani spending function with a predefined gamma parameter of –4. At interim analysis (3 February 2021), the Independent Data Monitoring Committee recommended that the study be stopped because this futility boundary was crossed.

The final statistical analysis plan was amended to remove a second planned interim analysis after 67% of participants completed day 28, due to fast enrollment. The amendment also mitigated potential substantial underestimation of the treatment effect following early study termination and study drug termination of many of the enrolled participants at the time futility was declared. The final efficacy analysis population included participants who completed the study or discontinued the study prior to 4 February 2021 or who completed the treatment period or discontinued the study drug prior to 4 February 2021.

The intention-to-treat (ITT) population (all randomized participants) was used for the primary analysis after interactions with regulators. Sensitivity analyses were repeated for both the modified ITT (mITT) population (ie, ITT participants who received at least 1 dose of the study drug and had mild disease at day 1) and the per protocol population (ie, mITT participants who did not substantially deviate from protocol procedures). The safety analysis population included participants who had at least 1 dose of the study drug.

The absolute difference in the risk of progression of COVID-19 was assessed using a stratified Miettinen and Nurminen method [15] with Cochran-Mantel-Haenszel weights [16] stratified by number of days since onset of symptoms at the time of randomization (<6 or ≥6 days). Disease resolution end points were assessed using a stratified 1-sided log-rank test. A stratified Cox proportional hazards (PH) model was used to estimate the hazard ratios and corresponding confidence intervals (CIs) associated with the rate of disease resolution for the study intervention relative to control. No adjustments were made for multiple comparisons. The proportion of participants at key time points with disease progression, disease resolution, AEs, and hospitalizations was summarized by treatment group, with 95% exact CIs and 2-sided *P* values.

## RESULTS

**Figure 1.**
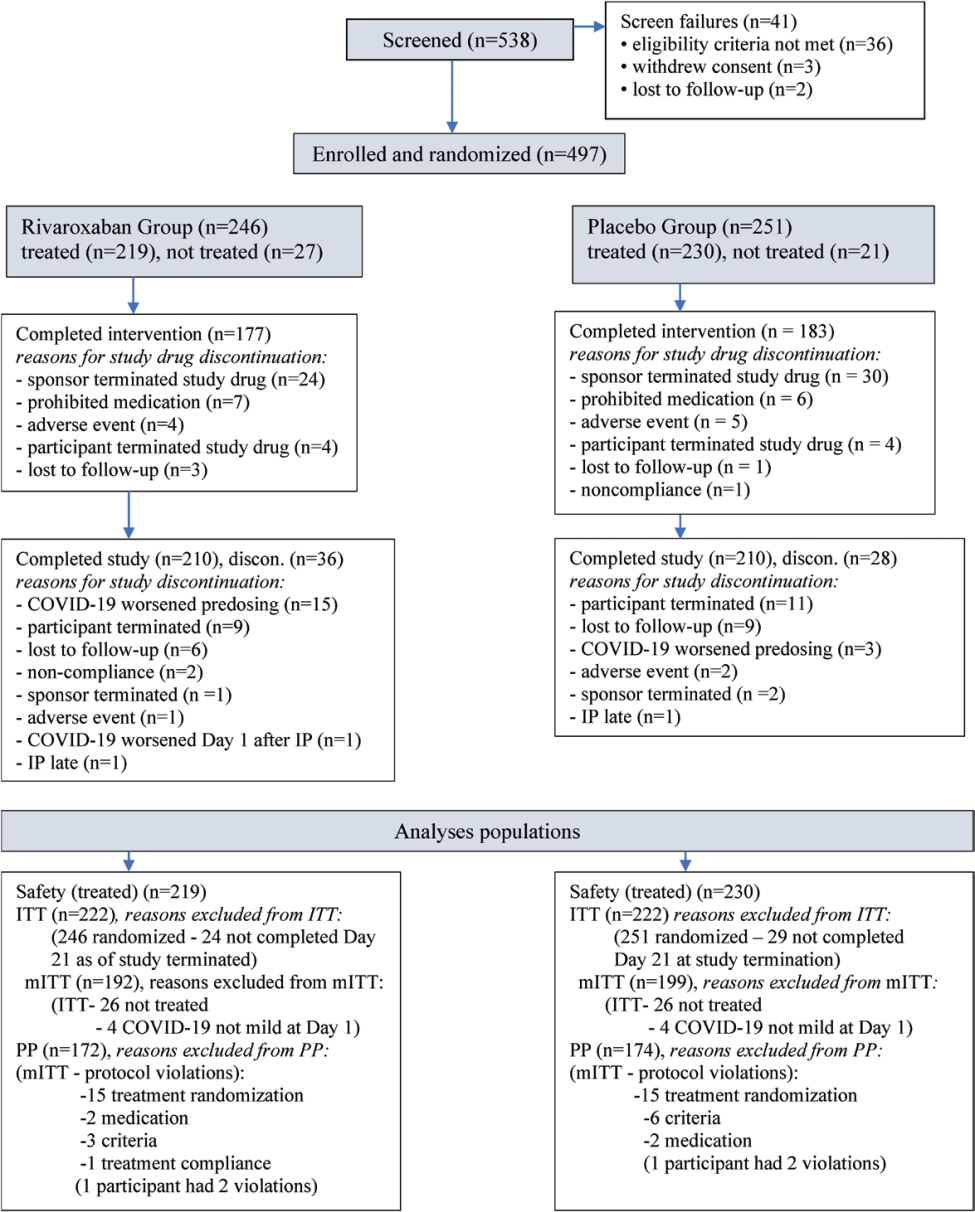
Participant flow diagram. COVID-19, coronavirus 2019; IP, investigational product/study drug; ITT, intention to treat; mITT, modified intention to treat; PP, per protocol.

Approximately 27 000 adults were prescreened using the Science 37 prescreening questionnaire (Supplementary Table 4) before entering the screening process. Between 16 August 2020 and 3 February 2021, 538 adults were screened and 497 were enrolled (246 in rivaroxaban and 251 in placebo). At study termination (4 February 2021), 321 (65%) had already completed the study. The numbers of participants in each analysis population were similar between treatment groups (Figure 1). Sixty-four participants discontinued the study and 89 discontinued the study drug (similar between groups). In both groups, the most common reasons for discontinuation were COVID-19 worsening before dosing, lost to follow-up, participant’s request, and early study termination by the sponsor.

Demographic and baseline characteristics were similar between groups (Table 1). The majority of participants were aged <65 years, female, and White. Sixty-nine percent had 2 or more comorbidities (risk factors for severe COVID-19), the most common being hypertension, obesity, diabetes, and other chronic diseases. Although a positive SARS-CoV-2 PCR and symptomatic status were required at screening, by day 1, 18% had a negative SARS-CoV-2 PCR, 0.7% were asymptomatic, and 4% had progressed to moderate or severe disease. During the study, participants had a mean study drug exposure of 18.6 days (vs 21 days for full exposure) and 82% had ≥75% compliance.

**Table 1.** Baseline Characteristics of the Intention-to-Treat Population

Characteristic at Day 1	Rivaroxaban (N = 222), n (%)	Placebo (N = 222), n (%)	Total (N = 444), n (%)
Median age, (min–max), years	49 (20–83)	49 (18–75)	49 (18–83)
<65	187 (84.2)	192 (86.5)	379 (85.4)
≥65	35 (15.8)	30 (13.5)	65 (14.6)
Male	96 (43.2)	81 (36.5)	177 (39.9)
Female	126 (56.8)	141 (63.5)	267 (60.1)
Race			
American Indian or Alaska Native	1 (.5)	2 (.9)	3 (.7)
Asian	0 (.0)	1 (.5)	1 (.2)
Black or African American	15 (6.8)	15 (6.8)	30 (6.8)
White	198 (89.2)	197 (88.7)	395 (89.0)
Mixed race	0 (.0)	0 (.0)	0 (.0)
Other	7 (3.2)	7 (3.2)	14 (3.2)
Unknown	1 (.5)	0 (.0)	1 (.2)
Ethnicity			
Hispanic or Latino	48 (21.6)	40 (18.0)	88 (19.8)
Not Hispanic or Latino	174 (78.4)	178 (80.2)	352 (79.3)
Not reported	0 (.0)	4 (1.8)	4 (.9)
Unknown	0 (.0)	0 (.0)	0 (.0)
Body mass index, kg/m^2^			
Median	35.2 (16.9–68.7)	33.2 (18.8–66.4)	34.7 (16.9–68.7)
<25	17 (7.7)	29 (13.1)	46 (10.4)
25 to < 35	88 (39.6)	96 (43.2)	184 (41.4)
≥35	117 (52.7)	97 (43.7)	214 (48.2)
Comorbidity factors			
Age ≥65 years	35 (15.8)	30 (13.5)	65 (14.6)
Presence of chronic pulmonary disease, chronic obstructive pulmonary disease, pulmonary hypertension	14 (6.3)	13 (5.9)	27 (6.1)
Diabetes mellitus (type 1 or type 2) requiring medication or insulin	57 (25.7)	66 (29.7)	123 (27.7)
Hypertension requiring at least 1 oral medication for intervention	106 (47.7)	124 (55.9)	230 (51.8)
Immunocompromised status due to disease	4 (1.8)	11 (5.0)	15 (3.4)
Participants with 2 or more comorbidity factors	154 (69.4)	151 (68.0)	305 (68.7)
Median duration of symptoms, days			
Median (min–max)	8.0 (2–14)	8.0 (2–12)	8.0 (2–14)
<6	24 (12.2)	35 (17.4)	59 (14.9)
≥6	172 (87.8)	166 (82.6)	338 (85.1)
Positive severe acute respiratory syndrome coronavirus 2 polymerase chain reaction test	158 (81.4)	163 (82.3)	321 (81.9)
Gates Medical Research Institute ordinal scale			
1 (asymptomatic)	2 (1.0)	1 (.5)	3 (.7)
2 (mild disease)	193 (93.2)	200 (97.6)	393 (95.4)
3 (moderate or severe disease)	12 (5.8)	4 (2.0)	16 (3.9)
World Health Organization ordinal scale			
0 (uninfected, no viral RNA detected)	2 (1.0)	0 (0)	2 (.5)
1 (asymptomatic, viral RNA detected)	0 (0)	1 (.5)	1 (.2)
2 (symptomatic, independent)	204 (98.6)	204 (99.5)	408 (99.0)
3 (symptomatic, assistance needed)	1 (.5)	0 (0)	1 (.2)

The primary end point showed approximately 20% (ITT population) experiencing disease progression, without a difference between groups (Table 2). There were also no differences between groups for the secondary end points in this population. The remaining results herein are focused on the mITT population, which is the most clinically relevant (Table 2).

**Table 2.** Proportion of Participants With Disease Progression, Symptoms Resolution, No Symptoms, and Hospitalization Through Day 28

	Intention-to-Treat Population			Modified Intention-to-Treat Population			Per Protocol Population		
Endpoint	Rivaroxaban (N = 222)	Placebo (N = 222)	Rivaroxaban vs Placebo Risk Difference [95% CI]	Rivaroxaban (N = 192)	Placebo (N = 199)	Rivaroxaban vs Placebo Risk Difference [95% CI]	Rivaroxaban (N = 172)	Placebo (N = 174)	Rivaroxaban vs Placebo Risk Difference [95% CI]
Proportion with disease progression, n (%) [CI]	46 (20.7) [15.8 to 26.4]	44 (19.8) [15.0 to 25.5]	1.0 [–6.4 to 8.4] *P* = .78	18 (9.4) [5.8 to 14.1]	23 (11.6) [7.7 to 16.6]	–2.2 [–8.4 to 4.0] *P* = .47	17 (9.9) [6.1 to 15.0]	16 (9.2) [5.5 to 14.2]	.6 [–5.7 to 7.1] *P* = .85
Proportion who met protocol-defined symptoms resolution,^a^ n (%) [CI]	132 (59.5) [52.9 to 65.8]	122 (55.0) [48.4 to 61.4]	4.5 [–4.7 to 13.6] *P* = .34	130 (67.7) [60.8 to 74.0]	119 (59.8) [52.9 to 66.4]	7.9 [–1.7 to 17.3] *P* = .106	118 (68.6) [61.4 to 75.2]	108 (62.1) [54.7 to 69.1]	6.5 [–3.6 to 16.4] *P* = .21
Proportion of asymptomatic participants^b^ (Gates MRI scale 1), n (%) [CI]	125 (56.3) [49.7 to 62.7]	108 (48.6) [42.1 to 55.2]	7.6 [–1.7 to 16.8] *P* = .11	123 (64.1) [57.1 to 70.6]	105(52.8) [45.8 to 59.6]	11.3 [1.5 to 20.9] *P* = .02	112 (65.1) [57.8 to 72.0]	97 (55.7) [48.3 to 63.0]	9.4 [–1.0 to 19.5] *P* = .08
Proportion with hospitalization, n (%) [CI]	3 (1.4) [.3 to 3.6]	7 (3.2) [1.4 to 6.1]	.43 [.11 to 1.65] *P* = .21	2 (1.0) [.2 to 3.4]	5 (2.5) [.9 to 5,5]	.41 [.08 to 2.07] *P* = .27	2 (1.2) [.2 to 3.8]	2 (1.1) [.2 to 3.7]	.99 [.14 to 6.85] *P* = .99

*P* = 2-sided *P* values. Risk difference is shown for all parameters except for proportion with hospitalization, for which relative risk is shown. Risk difference and relative risks are adjusted for the randomization stratification factor of days since onset of symptoms at time of randomization (<6 days vs ≥6 days).

Abbreviation: CI, confidence interval; MRI, Medical Research Institute.

^a^ Symptoms resolution is defined as disappearance or a return to baseline for all coronavirus disease 2019 (COVID-19)–related symptoms, except for anosmia and ageusia, through day 28.

^b^Asymptomatic is defined as lack of symptoms related to COVID-19 at day 28.

There were no differences in disease progression rates in the mITT population between groups overall and by subgroups (ie, by race, ethnicity, sex, duration of symptoms, age, types of comorbidities, and body mass index; Supplementary Figure 1). All progressions were to Gates MRI scale 3, except 1 placebo participant who progressed to Gates MRI scale 4.

**Figure 2.**
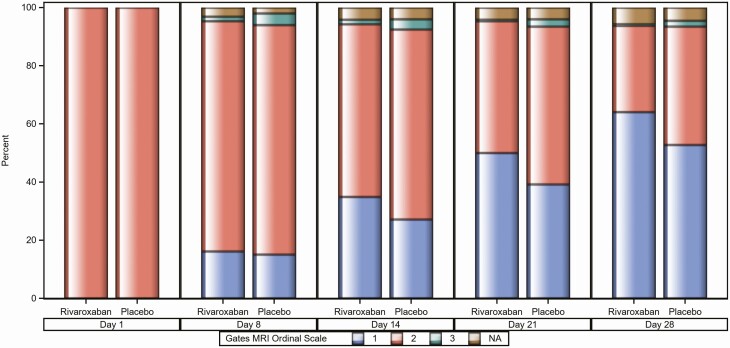
Proportion of participants within each Gates Medical Research Institute scale at days 1, 8, 14, 21, and 28 (modified intention-to-treat [mITT] population). Gates MRI ordinal scale: 1 = asymptomatic/symptoms similar to pre-coronavirus disease 2019 (COVID-19) status, 2 = mild, 3 = moderate or severe, 4 = critically ill, 5 = critically ill with invasive mechanical ventilation or extrapulmonary complication, 6 = critically ill with extracorporeal membrane oxygenation, and 7 = death. NA = Participants in mITT population who had no available Gates MRI scale value at post-baseline assessments.

**Figure 3.**
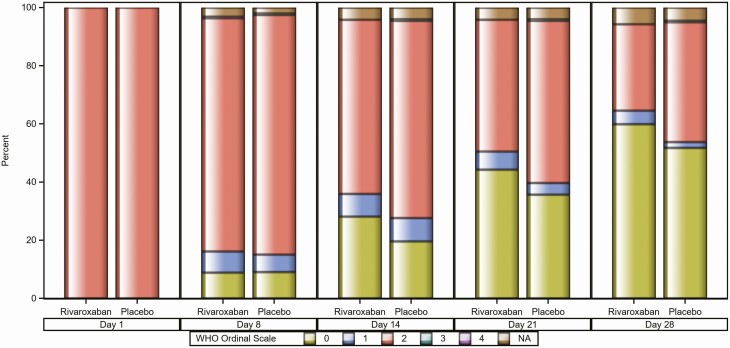
Proportion of participants within each WHO scale at days 1, 8, 14, 21, and 28 (modified intention-to-treat [mITT] population). WHO ordinal scale: 0 = uninfected; 1 = asymptomatic, viral RNA detected; 2 = symptomatic, independent; 3 = symptomatic, assistance needed; 4 = hospitalized, no oxygen therapy; 5 = hospitalized, oxygen by mask or nasal prongs; 6 = hospitalized, oxygen by NIV or high flow; 7 = intubation and mechanical ventilation, pO_2_/FiO_2_ ≥150 or SpO_2_/FiO_2_ ≥200; 8 = mechanical ventilation pO_2_/FiO_2_ <150 (SpO_2_/FiO_2_ <200) or vasopressors; 9 = mechanical ventilation pO_2_/FiO_2_ <150 and vasopressors, dialysis, or extracorporeal membrane oxygenation; and 10 = death. NA, participants in mITT Population who have no available WHO scale value at post-baseline assessments; NIV, noninvasive ventilation; WHO, World Health Organization.

There was a higher proportion of asymptomatic participants at day 28 in the rivaroxaban arm as defined by the Gates MRI scale 1 (*P* = .024; Table 2, Figure 2, and Supplementary Table 5), particularly among those with ≥6 days of symptoms at randomization. Similar findings were observed with the WHO ordinal scale (Figure 3).

At day 28, symptoms that persisted in ≥10% of participants were fatigue (16.9% vs 22.1%), cough (10.4% vs 15.8%), nasal congestion (12.6% vs 12.1%), anosmia (17.5% vs 22.1%), and ageusia (14.2% vs 16.3%) in the rivaroxaban and placebo groups, respectively. These symptoms occurred at a similar frequency between groups at baseline. The proportion of participants who had a SARS-CoV-2–negative test through day 28 did not differ between groups (Supplementary Table 6).

Nine participants were hospitalized due to COVID-19 pneumonia; however, 3 participants had not begun study intervention when hospitalized. During study treatment, 6 participants were hospitalized due to COVID-19 pneumonia, 2 in the rivaroxaban group (after 2 and 6 days of treatment) and 4 in the placebo group (after 1, 3, 3, and 8 days of treatment). Two additional placebo participants were hospitalized for pancreatitis and malignant hypertension/acute kidney injury, respectively.

The frequencies of any AEs and AEs resulting in study intervention discontinuation were similar between groups. In the rivaroxaban group, slightly more AEs related to study intervention but fewer serious AEs were reported (Table 3). There were no deaths during the study period. Two serious AEs that did not require hospitalization occurred in 2 placebo participants. One experienced DVT diagnosed 13 days after stopping study drug and 11 days after being discharged for a COVID-19 pneumonia hospitalization. The other participant was diagnosed with recurrent breast cancer after receiving 11 days of placebo.

**Table 3.** Summary of Adverse Events

	Rivaroxaban (N = 219)	Placebo (N = 230)
**Type of Event**	**n (%)**	**n (%)**
Any AEs	35 (16.0)	36 (15.7)
Related AEs	6 (2.7)	1 (.4)
AEs resulting in discontinuation of study intervention	4 (1.8)	5 (2.2)
Deaths	0 (.0)	0 (.0)
Grade 4 or grade 5 AE	0 (.0)	0 (.0)
Serious AEs^a^	2 (.9)	7 (3.0)
Severe hypersensitivity	0 (.0)	0 (.0)
Major bleeding	0 (.0)	0 (.0)
Clinically relevant nonmajor bleeding^b^	5 (2.3)	2 (.9)

A participant with multiple AEs with different grades was counted only once under the highest grade for the n and % values.

Abbreviation: AE, adverse event.

^a^ None of the AEs were determined to be related to the study drug, per investigator assessment.

^b^ Not clinically significant (*P* = .27).

As shown in Table 3, no participant experienced major bleeding; clinically relevant, nonmajor bleeding was rare and included 3 participants with hematuria, 2 with hemorrhoidal bleeding in the rivaroxaban group (2.3%), and 1 with rectal bleeding and 1 with blood in stool in the placebo group (.9%). One rivaroxaban participant had minimal bilateral subconjunctival hemorrhage. All participants discontinued the study drug due to the bleeding events except for 1 participant with hematuria reported 13 days after completing rivaroxaban.

## DISCUSSION

In adults with mild COVID-19 at high risk for severe disease, administration of rivaroxaban for 21 days did not reduce progression to moderate or severe disease compared with placebo. We did observe a higher proportion of asymptomatic participants at day 28 after rivaroxaban treatment. The incidence of hospitalization was low in both groups, but more cases of COVID-19 pneumonia and vascular events were observed in placebo compared with rivaroxaban recipients. Rivaroxaban was well tolerated with few AEs and minor bleeding events related to the drug.

We enrolled high-risk adults who presented with mild disease during the first week of their illness to intervene, as the disease is thought to transition from the active viral replication phase to the inflammatory phase, and before disease progressed [17]. Rivaroxaban does not possess direct antiviral or antiinflammatory properties but it may mitigate the consequences of SARS-CoV-2 replication and inflammation. We posited that rivaroxaban could prevent COVID-19–associated VTE and microthrombi in the lungs and other organs that have been theorized to cause COVID-19 symptoms and progression. This could potentially explain the observation that more participants achieved asymptomatic status. It appears that the observed differences between groups occurred after day 14 of the study and in participants who had longer time from onset of symptoms to randomization. We postulate that the persistence of symptoms after the second week of illness may be the result of microthrombi against which rivaroxaban is exerting a positive effect. It is possible that administration of rivaroxaban during mild disease and within the first 2 weeks of illness was too early, as a major shift to a proinflammatory and coagulopathy state occurs when disease transitions from mild to moderate [18]. We did not collect blood for biomarkers such as D-dimer in this community-based study. However, such examination should be considered in future studies to aid in potentially identifying a population that would most benefit from prophylactic anticoagulant. The daily dose of 10 mg rivaroxaban for 21 days may have been insufficient. The selection was based on the indication for VTE prophylaxis in acutely ill patients (10 mg daily for 31–39 days after hospital discharge) and a safety profile suitable for remote monitoring in our study. Fatigue, cough, anosmia, and ageusia appeared less frequently in the rivaroxaban group at day 28. Whether rivaroxaban could benefit long-term COVID-19 symptoms is unknown and should be further investigated.

Our study does not rule out a possible effect of rivaroxaban on disease progression and hospitalization. The sample size was too small to detect an effect size lower than 35% for disease progression, and hospitalizations were rare. Our primary end point of disease progression was based on the Gates MRI scale that, like other instruments, was not validated at the time of use. We established the scale to provide more granular definitions for mild disease. It may have inaccurately captured disease progression due to the subjective nature of shortness of breath that defined the majority of the progression cases. Moreover, we may not have enrolled people with the highest risks for severe COVID-19, as reflected by the low representations of the elderly, minorities, and people with significant comorbidities. The recruitment via social media and the virtual trial design posed challenges in engaging these groups. The need to ship supplies to participants’ homes delayed time to first dose of study drug by about 2 days, which may have contributed to a proportion of participants experiencing negative SARS-CoV-2 PCR and/or COVID-19 progression at day 1.

The National Institutes of Health COVID-19 guidelines do not recommend anticoagulants for nonhospitalized patients for the prevention of VTE or arterial thrombosis unless the patient has other indications for the therapy or is participating in a clinical trial [12]. A large nationwide registry during the COVID-19 pandemic in Sweden showed no association between outpatient direct oral anticoagulant (DOAC) use and risk of hospitalization for COVID-19 [19]. As of July 2020, there were at least 75 randomized clinical trials of antithrombotic therapy in COVID-19, including at least 7 studies of prophylactic DOAC in nonhospitalized patients, which vary in study populations and designs, type of drugs, and drug dosing [5]. Timing of administration and dose level of antithrombotics appear critical. Therapeutic rivaroxaban or enoxaparin given to hospitalized patients may not significantly improve outcomes and could increase bleeding events [20, 21].

There is limited capacity to treat severe disease in LMICs [22]. It is of high public health interest to identify mild disease treatments with greater potential for scale-up (eg, oral administration, stable at room temperature, and low cost), but none have gained regulatory approval. Trial results of oral drugs such as hydroxychloroquine have been disappointing [23]. Modest benefits were observed for colchicine in reducing the risk of death and hospitalization [24] and for inhaled budesonide in reducing urgent care visits and hastening symptoms resolution [25]. Monoclonal antibody (mAbs) treatments for people with mild to moderate COVID-19 and at risk for severe disease have been granted US Food and Drug Administration emergency use authorization, including bamlanivimab/etesevimab and casirivimab/imdevimab combinations. Their requirement for an intravenous or injectable administration during the first few days of symptoms poses an uptake challenge. The relatively high cost and cold chain requirements render mAbs unlikely to be readily available in LMICs.

Our study did not demonstrate an impact of rivaroxaban on disease progression in adults with mild COVID-19 and at high risk for severe COVID-19. The potential effect of rivaroxaban on symptoms resolution should be confirmed in larger studies. A number of trials are already ongoing to evaluate the effects of rivaroxaban [26] and apixaban [27] on thromboembolic events, hospitalizations, and deaths. There remains a critical global public health gap in identifying effective therapies that could be scaled up for at-risk people in the outpatient setting to accelerate disease resolution and to prevent disease progression, hospitalizations, and deaths.

## Supplementary Data

Supplementary materials are available at *Clinical Infectious Diseases* online. Consisting of data provided by the authors to benefit the reader, the posted materials are not copyedited and are the sole responsibility of the authors, so questions or comments should be addressed to the corresponding author.

ciab813_suppl_Supplemental_Table_S1

ciab813_suppl_Supplemental_Table_S2

ciab813_suppl_Supplemental_Table_S3

ciab813_suppl_Supplemental_Table_S4

ciab813_suppl_Supplemental_Table_S5

ciab813_suppl_Supplemental_Table_S6

ciab813_suppl_Supplemental_Figure_S1

ciab813_suppl_Supplementary_Data

## Notes

***Acknowledgments*.** The authors are grateful to the study participants for their contribution to this study. They thank the study investigators Shari DeSilva, Shirley Nagel, and Neal Warshoff for their contribution to this study. They thank the members of the Gates MRI RESPOND-1 Study Group: Russell Weiner, Jane Kramer, Ram Narayanan, Chantal Sarmanoukian, Micha Levi, Cari Tsinovoi, Maryann Livolsi, Sally Jennings, Alan Gibson, Alexander Schmidt, Charles Wells, W James Alexander, MD, MPH, Joel Alvarez, Karen Ward, Melissa Tice, Apurve Dhariwal, Laura Gibson, Lynn Agostinelli, Caroline Alix, and Paul Lithotomos. In addition, they thank their partners the contract research organization (CRO), PPD, particularly Irak Leon and Paul Carter for their contributions, along with many other PPD team members, and Kaila Bode-Shaw from Science37 and the rest of Science37 team members. They thank Debbie Daehnick for providing medical writing support, which was funded by the Gates MRI in accordance with Good Publication Practice (GPP3) guidelines (http://www.ismpp.org/gpp3). They also thank the regulatory authorities who reviewed this study, US Food and Drug Administration and UK Medicines & Healthcare Products Regulatory Agency, for their tireless work during the pandemic to provide thorough and expedient reviews of clinical trial applications.

***Financial support.*** This work was supported by a grant from Gates Philanthropy Partnerships and the Bill & Melinda Gates Foundation.

***Potential conflicts of interest.*** E. G. reports support from AstraZeneca, Genentech, Regeneron, TEVA, and DBV; consulting fees from P&T Ascellahealth; payment/honoraria from TEVA (Digihaler) and Genentech (Omalizumab); and serving on Regeneron Pediatric Asthma Advisory Board and ACAAI Patient Advocacy Board. All other authors report no potential conflicts. All authors have submitted the ICMJE Form for Disclosure of Potential Conflicts of Interest. Conflicts that the editors consider relevant to the content of the manuscript have been disclosed.
