# Supplementary material for: Randomized Study of Rivaroxaban vs Placebo on Disease Progression and Symptoms Resolution in High-Risk Adults With Mild Coronavirus Disease 2019
Source: Clin Infect Dis. 2021 Sep 15;75(1):e473–81. doi: 10.1093/cid/ciab813 (PMC8522357; doi:10.1093/cid/ciab813)
Supplement: ciab813_suppl_Supplemental_Table_S1 [file ciab813_suppl_supplemental_table_s1.docx]

**Supplemental Table 1: Eligibility Criteria**

| **Inclusion Criteria**  *participants were eligible to be included in the study only if all of the following criteria applied* |
| --- |
| **Age** |
| 1. ≥18 years of age at the time of informed consent |
| **Type of Participant** |
| 1. Participants must have been at high-risk for COVID-19 progression by fulfilling at least **one** of the following criteria at screening:  - Age ≥65 years - Presence of chronic pulmonary disease, chronic obstructive pulmonary disease (COPD), pulmonary hypertension - Diabetes mellitus (type 1 or type 2), requiring oral medication or insulin for treatment - Hypertension, requiring at least one oral medication for treatment - Immunocompromised status due to disease (e.g., those living with human immunodeficiency virus with a CD4 T-cell count of <200/mm^3^) - Immunocompromised status due to medication (e.g., taking 20 mg or more of prednisone equivalents a day, anti-inflammatory monoclonal antibody therapies, cancer therapies) - Any chronic disease that is associated with high risk for severe COVID disease in the opinion of the site investigator - Body mass index ≥35 kg/m^2^ (based on self-reported weight and height). |
| **COVID-19 Characteristics** |
| 1. Documented positive SARS-CoV-2 diagnostic testing with a sample collected ≤10 days of screening. 2. Symptomatic for COVID-19 for ≤7 days at time of randomization   (symptomatic was defined as having at least one of the following symptoms of COVID-19 that was of new onset or has worsened from baseline: fever, chills, myalgia, arthralgia, headache, fatigue, cough, sore throat, nasal congestion, nausea, vomiting, or diarrhea). |
| **Informed** **Consent** |
| 1. Capable of giving informed consent, which includes compliance with the requirements and restrictions listed in the ICF and in this protocol |
| **Sex**   1. Male or female |
| **Other Requirements:**   1. Agreed to participate in all remote, in-person or home visits as required in the protocol and to provide updated contact information as necessary |
| 1. Female of childbearing potential must agree to practice adequate contraception during the study |

| **Exclusion Criteria**  *participants were excluded from the study if any of the following criteria applied* |
| --- |
| **Medical Conditions or history** |
| 1. Currently hospitalized or under immediate consideration for hospitalization at screening and Day 1 2. Have new onset shortness of breath or increased shortness of breath from pre-COVID-19 (for people with known COPD) at screening and Day 1 3. Hypoxemia (oxygen saturation<94% in ambient air or oxygen saturation below pre-COVID-19 oxygen saturation [if known] for people with known COPD) at Day 1 4. Require supplemental oxygen (new requirement or increase in requirement from pre-COVID-19 condition) at screening and Day 1 5. Have a history of (in the past 3 months) or current active pathological bleeding 6. Have a history of hemorrhagic stroke or intracranial hemorrhage 7. Have a recent severe head trauma within 30 days which includes concussion, skull fracture or hospitalization for head injury 8. Have known intracranial neoplasm, cerebral metastases, arteriovenous malformation or aneurysm 9. Have history of pregnancy-related hemorrhage 10. Have active gastroduodenal ulcer or other gastrointestinal bleeding diagnosed in the past 3 months 11. Currently are in a hemodynamically unstable state 12. Currently require thrombolysis or pulmonary embolectomy 13. Have history of severe hypersensitivity reaction to Xarelto® 14. Currently have a prosthetic heart valve 15. Have known diagnosis of triple positive antiphospholipid syndrome 16. Have known diagnosis of chronic kidney disease (stage IV or receiving dialysis) 17. Have a history of thrombocytopenia or known platelet count < 100,000 cells/mm^3^ 18. Have a history of bronchiectasis and pulmonary cavitation 19. Have active cancer (eg, receiving chemotherapy or treatment for complication of the active cancer) |
| **Other:** |
| 1. Had epidural or neuraxial anesthesia or spinal puncture in the past 2 weeks or plan to undergo these procedures during the study 2. Had surgery in the past 4 weeks or plan to undergo surgery during the study 3. Currently is pregnant or plans to become pregnant 4. Currently is breastfeeding 5. Share household with an enrolled participant in this study 6. Co-enrollment in any clinical trial that includes prohibited procedures (spinal puncture or surgery) or that includes treatments within the same drug class as rivaroxaban or treatments for which co-administration with rivaroxaban were prohibited |
| **Medications:**   1. Currently using and plan to use the following medications during the study  - Rivaroxaban or drugs in the same class - Dual anti-platelets therapy - Other anticoagulants - Combined P-gp and CYP3A inhibitors and inducers |
