# Supplementary material for: Randomized Study of Rivaroxaban vs Placebo on Disease Progression and Symptoms Resolution in High-Risk Adults With Mild Coronavirus Disease 2019
Source: Clin Infect Dis. 2021 Sep 15;75(1):e473–81. doi: 10.1093/cid/ciab813 (PMC8522357; doi:10.1093/cid/ciab813)
Supplement: ciab813_suppl_Supplemental_Table_S2 [file ciab813_suppl_supplemental_table_s2.docx]

**Supplemental Table 2: Gates MRI COVID-19 Ordinal Scale Clinical Endpoint Definitions**

| **Scale** | **Category** | **Endpoint definition** |
| --- | --- | --- |
| 1 | Asymptomatic/symptoms similar to pre-COVID status | No symptoms and signs AND |
|  |  | No limitation of daily activities |
| 2 | Mild | Symptomatic^a^ AND |
|  |  | No shortness of breath AND |
|  |  | No hypoxemia (O2 saturation ≥94% in ambient air) |
| 3 | Moderate or severe | Symptomatic AND |
|  |  | Shortness of breath OR tachypnea (respiratory rate ≥ 20 min)^b^ OR hypoxemia (<94% in ambient air)^b^ |
| 4 | Critically ill | Symptomatic AND |
|  |  | Receiving high flow oxygen OR non-invasive mechanical ventilation |
| 5 | Critically ill with invasive mechanical ventilation or extrapulmonary complication | Symptomatic AND |
|  |  | Receiving invasive mechanical ventilation OR Life threatening or debilitating extrapulmonary complications |
| 6 | Critically ill with Extra-Corporeal Membrane Oxygenation (ECMO) | Symptomatic AND |
|  |  | Receiving ECMO |
| 7 | Death | Death |

This scale is aligned with other well-accepted scales (e.g., WHO scale, Supplemental Table 3).

^a^ Symptomatic is defined as having at least one of the following symptoms: defined as fever, chills, myalgia, arthralgia, headache, fatigue, cough, sore throat, nasal congestion, anosmia, ageusia, nausea, vomiting, or diarrhea.

^b^For known COPD participants, moderate or severe category required either worsening of shortness of breath, respiratory rate, or oxygen saturation, from pre-COVID-19 status.
