# Supplementary material for: Randomized Study of Rivaroxaban vs Placebo on Disease Progression and Symptoms Resolution in High-Risk Adults With Mild Coronavirus Disease 2019
Source: Clin Infect Dis. 2021 Sep 15;75(1):e473–81. doi: 10.1093/cid/ciab813 (PMC8522357; doi:10.1093/cid/ciab813)
Supplement: ciab813_suppl_Supplemental_Table_S4 [file ciab813_suppl_supplemental_table_s4.docx]

**Supplemental Table 4: Pre-Screening Questionnaire**

- - - 1. Are you over 18 years of age?
         - If YES - *Continue with script*
         - If NO - *Thank the respondent for their time*

*If the respondent isn’t over 18, they aren’t eligible for the trial. Go directly to the ineligible language below.*

- - - 1. Do you have any of the following - diabetes, high blood pressure, COPD, cancer, HIV/AIDS, obesity, an organ transplant, or any disease that puts you at increased risk from COVID-19?
         - If YES - *Continue with script*
         - If NO - *Thank the respondent for their time*
         - *DO NOT KNOW - Continue with the script*

*If the respondent doesn’t have any of these conditions, they aren’t eligible for the trial. Go directly to the ineligible language below.*

- - - 1. Have you tested positive for COVID-19?
         - If YES *- Ask when the test was performed.*
         - If NO *- Ask when they plan to be tested.*
         - *DO NOT KNOW - Continue with script*
      2. Are you taking Xarelto?
         - If YES - Thank the respondent for their time. If NO - *Continue with the script*
         - *DO NOT KNOW - Continue with the script*

*If the respondent is taking Xarelto, they aren’t eligible for the trial. Go directly to the ineligible language.*

- - - 1. Have you had any of the following symptoms for ≤72 hours *(defined as having at least two of the following symptoms of COVID-19 that are new, or that have worsened, and include -*
         - Fever
         - Chills
         - Muscle pain
         - Joint pain
         - Headache
         - Fatigue
         - Cough
         - Sore throat
         - Nasal congestion
         - Loss of smell
         - Loss of taste
         - Nausea
         - Vomiting, or
         - Diarrhea

*If the only symptoms the respondent is experiencing are the loss of smell and taste, they are ineligible for the study. Go directly to the ineligible language.
If the respondent doesn’t have any of the symptoms or only has one of the symptoms, they are ineligible to proceed with the screening process at this time. However, if the respondent is eligible otherwise (based on their responses to the questions in this script), and is still interested in participating in the study, they will have the option to sign the pre-screening ICF and have Science 37 contact them on a reoccurring bases to see if they develop any additional symptoms.*

- - - 1. Are you currently hospitalized or under immediate consideration for hospitalization?
         - If YES - *Thank them for their time after confirming*
         - If NO - *Continue with script*
      2. Do you require supplemental oxygen (new requirement or increase in requirement from pre-COVID-19 condition)
         - If YES - *Thank you for your time after confirming*
         - If NO - *Continue with script*
      3. Do you have a history of hemorrhagic stroke or intracranial hemorrhage?
         - *If YES - Thank you for your time after confirming*
         - *If NO - Continue with script*
      4. Have you had any recent head trauma (concussion, skull fracture, hospitalization for a head injury) in the past 30 days?
         - *If YES - Thank you for your time after confirming*
         - *If NO - Continue with script*
      5. Do you have any known intracranial neoplasm, cerebral metastases, arteriovenous malformation or aneurysms?
         - *If YES - Thank you for your time after confirming*
         - *If NO - Continue with script*
      6. Are you currently in a hemodynamically unstable state?
         - *If YES - Thank you for your time after confirming*
         - *If NO - Continue with script*
      7. Will you in the near future require thrombolysis or pulmonary embolectomy?
         - *If YES - Thank you for your time after confirming*
         - *If NO - Continue with script*
      8. Have you been diagnosed with triple positive antiphospholipid syndrome?
         - *If YES - Thank you for your time after confirming*
         - *If NO - Continue with script*
      9. Do you have a history of having a low platelet count (< 100cells/mm3)?
         - *If YES - Thank you for your time after confirming*
         - *If NO - Continue with script*
      10. Do you have a history of bronchiectasis and pulmonary cavitation?
          - *If YES - Thank you for your time after confirming*
          - *If NO - Continue with script*
      11. Are you currently receiving treatment for cancer?
          - *If YES - Thank you for your time after confirming*
          - *If NO - Continue with script*
      12. Are you currently using or plan to use the following medications during the study?
          - *Rivaroxaban (or a drug in the same class)*
          - *Dual antiplatelets therapy*
          - *Other anticoagulants*
          - *Combined P-gp and CYP3A inhibitors and inducers*

*If YES - Thank you for your time after confirming*

*If NO - Continue with script*

- - - 1. (For females of child bearing potential) Are you pregnant or breastfeeding?
         - If YES - Thank you for your time
         - If NO - Continue with script
      2. (For females of child bearing potential*)* Will you agree to practice adequate contraception during the study?
         - If YES - *Continue with script*
         - If NO - *Thank you for your time*
      3. (For females) Are you currently or have you had any pathological bleeding in the past three months?
         - If YES - *Thank you for your time*
         - If NO - *Continue with the script*
      4. (For females) Have you had a pregnancy-related hemorrhage (uncontrollable bleeding)?
         - If YES - *Thank you for your time*
         - If NO - *Continue with the script*
         - DO NOT KNOW - *Continue with script*
      5. Have you had an active gastroduodenal (stomach or intestinal) ulcer diagnosed in the past 3 months
         - If YES - *Thank them for their time after reconfirming.*
         - If NO - *Continue with the script*
         - *DO NOT KNOW- Continue with the script*
      6. Have you had history of severe hypersensitivity (allergic) reaction to Xarelto®
         - If YES - *Thank them for their time after reconfirming.*
         - If NO - *Continue with the script*
         - *DO NOT KNOW- Continue with the script*
      7. Do you currently have a prosthetic heart valve?
         - If YES - *Thank them for their time after reconfirming.*
         - If NO - *Continue with the script*
         - *DO NOT KNOW- Continue with the script*
      8. Have you been diagnosed chronic kidney disease? *(stage IV or receiving dialysis)*
         - If YES - *Thank them for their time after reconfirming.*
         - If NO - *Continue with the script*
         - *DO NOT KNOW- Continue with the script*
      9. Have you been diagnosed with cirrhosis (long-term damage to the liver) This is often caused by alcohol, hepatitis B, or hepatitis C?
         - If YES - *Thank them for their time after reconfirming.*
         - If NO - *Continue with the script*
         - *DO NOT KNOW- Continue with the script*
      10. Have you had an epidural or neuraxial anesthesia or spinal puncture in the past two weeks? Do you plan to undergo these procedures during the study?
          - If YES - *Thank them for their time after reconfirming.*
          - If NO - *Continue with the script*
          - *DO NOT KNOW- Continue with the script*
      11. Have you undergone surgery in the past four weeks, or do you plan to undergo surgery in the next four weeks or so?
          - If YES - *Thank them for their time after reconfirming.*
          - If NO - *Continue with the script*
      12. Is there anyone else in your household that is participating in this study?
          - If YES - *Thank them for their time after reconfirming.*
          - If NO - *Continue with the script*
      13. Are you currently enrolled in another clinical trial?
          - If YES - *Thank them for their time after reconfirming.*
          - If NO - *Continue with the script*
