# Supplementary material for: Randomized Study of Rivaroxaban vs Placebo on Disease Progression and Symptoms Resolution in High-Risk Adults With Mild Coronavirus Disease 2019
Source: Clin Infect Dis. 2021 Sep 15;75(1):e473–81. doi: 10.1093/cid/ciab813 (PMC8522357; doi:10.1093/cid/ciab813)
Supplement: ciab813_suppl_Supplemental_Table_S5 [file ciab813_suppl_supplemental_table_s5.docx]

**Supplemental Table 5: Proportion of Asymptomatic Participants (Gates MRI Scale 1) at Day 28 (mITT Population)**

|  | | | | Risk Difference ^a^ | | |
| --- | --- | --- | --- | --- | --- | --- |
|  | N | Proportion ^b^ n (%) | 95% CI ^b^ | Difference (%) | 95% CI | 2-sided P-value |
| **Adjusted analysis** | | | | | | |
| Rivaroxaban | 192 | 123 (64.1) | (57.1, 70.6) | 11.3 | 1.5, 20.9 | 0.02 |
| Placebo | 199 | 105 (52.8) | (45.8, 59.6) |  |  |  |
| **Days since onset of symptoms (<6 days)** | | | | | | |
| Rivaroxaban | 90 | 58 (64.4) | (54.2, 73.8) | 9.0 | -5.3, 22.9 | 0.22 |
| Placebo | 92 | 51 (55.4) | (45.2, 65.3) |  |  |  |
| **Days since onset of symptoms (≥6 days)** | | | | | | |
| Rivaroxaban | 102 | 65 (63.7) | (54.1, 72.6) | 13.3 | -0.2, 26.2 | 0.05 |
| Placebo | 107 | 54 (50.5) | (41.0, 59.9) |  |  |  |

^a^ Adjusted for the randomization stratification factor of days since onset of symptoms at time of randomization (=6 days)

^b^ Proportion=n/N, 95% CIs are based on the conditional binomial Clopper-Pearson method with mid-p correction
