# Supplementary material for: Randomized Study of Rivaroxaban vs Placebo on Disease Progression and Symptoms Resolution in High-Risk Adults With Mild Coronavirus Disease 2019
Source: Clin Infect Dis. 2021 Sep 15;75(1):e473–81. doi: 10.1093/cid/ciab813 (PMC8522357; doi:10.1093/cid/ciab813)
Supplement: ciab813_suppl_Supplemental_Table_S6 [file ciab813_suppl_supplemental_table_s6.docx]

**Supplemental Table 6: Proportion of Participants With SARS-CoV-2 Negative Diagnostic Test Through Day 28 (mITT Population)**

|  | **N** | **Proportion^b^ n (%)** | **2-sided 95% CI ^b^** | **Risk difference ^a^ (%)** | **2-sided 95% CI** | **2-sided P-value** |
| --- | --- | --- | --- | --- | --- | --- |
| **Adjusted analysis** | | | | | | |
| Rivaroxaban | 192 | 167 (87.0) | [81.6, 91.2] | -1.9 | (-8.5, 4.6) | 0.56 |
| Placebo | 199 | 177 (88.9) | [84.0, 92.8] |  |  |  |
| **Days since onset of symptoms (<6 days)** | | | | | | |
| Rivaroxaban | 90 | 73 (81.1) | [72.0, 88.2] | -1.5 | (-12.9, 9.9) | 0.79 |
| Placebo | 92 | 76 (82.6) | [73.8, 89.4] |  |  |  |
| **Days since onset of symptoms (≥6 days)** | | | | | | |
| Rivaroxaban | 102 | 94 (92.2) | [85.7, 96.3] | -2.2 | (-9.9, 5.0) | 0.52 |
| Placebo | 107 | 101 (94.4) | [88.7, 97.7] |  |  |  |

^a^ Adjusted for the randomization stratification factor of days since onset of symptoms at time of randomization (<6 days vs. ≥6 days).

^b^ Proportion=n/N, 95% CIs are based on the conditional binomial Clopper-Pearson method with mid-p correction.
