# Supplementary figures and images for: Randomized Study of Rivaroxaban vs Placebo on Disease Progression and Symptoms Resolution in High-Risk Adults With Mild Coronavirus Disease 2019
Source: Clin Infect Dis. 2021 Sep 15;75(1):e473–81. doi: 10.1093/cid/ciab813 (PMC8522357; doi:10.1093/cid/ciab813)

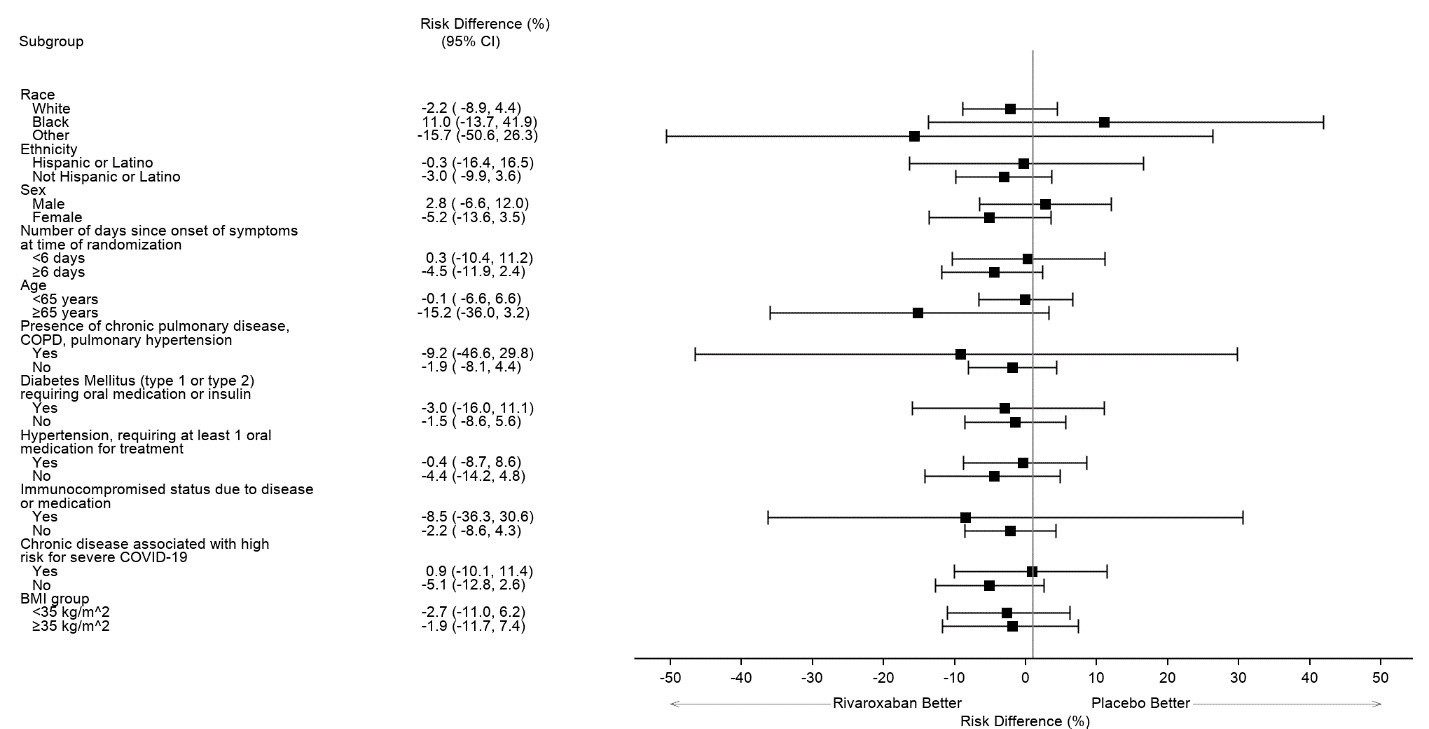

Supplement: ciab813_suppl_Supplemental_Figure_S1 [file ciab813_suppl_supplemental_figure_s1.jpeg]
