# Supplementary material for: Randomized Study of Rivaroxaban vs Placebo on Disease Progression and Symptoms Resolution in High-Risk Adults With Mild Coronavirus Disease 2019
Source: Clin Infect Dis. 2021 Sep 15;75(1):e473–81. doi: 10.1093/cid/ciab813 (PMC8522357; doi:10.1093/cid/ciab813)
Supplement: ciab813_suppl_Supplementary_Data [file ciab813_suppl_supplementary_data.docx]

**Protocol Title**: A randomized, controlled, Phase 2b study to evaluate safety and efficacy of rivaroxaban (Xarelto®) for high risk people with mild COVID-19

**Short Title:** A randomized, controlled, Phase 2b trial to evaluate safety and efficacy of rivaroxaban

Protocol Number: Gates MRI- COD-01-T01-01 Amendment 2

Compound: Xarelto® (rivaroxaban), approved for use in the US since 2011

Study Phase: 2b

**Sponsor Name:** Bill & Melinda Gates Medical Research Institute (Gates MRI)

**Legal Registered Address:**

| One Kendall Square |
| --- |
| Building 600, Suite 6-301 |
| Cambridge, MA 02139 |

**Regulatory Agency Identifier (Investigational New Drug [IND]) Number:** 151445

Medical Monitor/s name/s and contact information will be provided separately.

**Approval Date:** Version 4, Amendment 2, 27 January 2021

Refer to Section 13 for version history and amendment revisions.

**Version 4, Amendment 2 Sponsor Signatory**

*See appended electronic signature page*

| Jintanat Ananworanich, M.D., Ph.D.  Clinical Development Leader  Bill & Melinda Gates Medical Research Institute |  | **Date** |
| --- | --- | --- |

Table of Contents

[List of Abbreviations and Terms 7](#_Toc62640042)

[1. Synopsis 8](#_Toc62640043)

[2. Study Schedule of Procedures 11](#_Toc62640044)

[3. Background 12](#_Toc62640045)

[3.1. Severe Acute Respiratory Syndrome Coronavirus-2 12](#_Toc62640046)

[3.1.1. SARS-CoV-2 Epidemiology and Disease 12](#_Toc62640047)

[3.1.2. Therapeutic Approaches for COVID-19 13](#_Toc62640048)

[3.1.3. SARS-CoV-2 Immunothrombosis and Thromboembolic Events 13](#_Toc62640049)

[3.2. Rivaroxaban 14](#_Toc62640050)

[3.2.1. Use in Specific Populations 16](#_Toc62640051)

[3.2.2. Rationale for the Use of Rivaroxaban for Treatment of Mild COVID-19 16](#_Toc62640052)

[3.2.2.1. Rationale for a Limit of 7 Days of Mild COVID-19 Symptoms Prior to Randomization 16](#_Toc62640053)

[3.2.3. Benefit/Risk Assessment 17](#_Toc62640054)

[3.2.3.1. Benefit/Risk Assessment of Rivaroxaban 17](#_Toc62640055)

[3.2.4. Rivaroxaban Mechanism of Action 17](#_Toc62640056)

[3.2.5. Rivaroxaban Pharmacokinetics 18](#_Toc62640057)

[4. Objectives and Endpoints 19](#_Toc62640058)

[5. Study Design 20](#_Toc62640059)

[5.1. Overall Design 20](#_Toc62640060)

[5.2. Study Population 20](#_Toc62640061)

[5.3. Recruitment 20](#_Toc62640062)

[5.4. Pre-Screening for the Study 21](#_Toc62640063)

[5.4.1. Pre-Screening Inclusion Criteria 22](#_Toc62640064)

[5.4.2. Pre-Screening Procedures 23](#_Toc62640065)

[5.5. Screening for the Study 24](#_Toc62640066)

[5.5.1. Inclusion and Exclusion Criteria 24](#_Toc62640067)

[5.5.2. Exclusion Criteria 25](#_Toc62640068)

[6. Study Intervention- Rivaroxaban or Placebo 26](#_Toc62640069)

[6.1. Rationale for Study Intervention Selection 27](#_Toc62640070)

[6.2. Management of Participants to Limit Risks of SARS-CoV-2 Transmission 27](#_Toc62640071)

[6.3. Guidance on COVID-19 to Ensure Participant and Healthcare Worker Safety 27](#_Toc62640072)

[6.4. Dose Modification and Toxicity Management 27](#_Toc62640073)

[6.4.1. Definitions of Bleeding Severity 28](#_Toc62640074)

[6.4.1.1. Definition of Major Bleeding 28](#_Toc62640075)

[6.4.1.2. Definition of Clinically Relevant Non-Major Bleeding 28](#_Toc62640076)

[6.4.1.3. Definition of Minimal Bleeding 29](#_Toc62640077)

[6.5. Safety Monitoring 29](#_Toc62640078)

[6.6. Study Pausing Guidelines 29](#_Toc62640079)

[6.7. Masking 30](#_Toc62640080)

[6.8. Preparation/Handling/Storage/Accountability 30](#_Toc62640081)

[6.9. Study Intervention Compliance 30](#_Toc62640082)

[6.10. Concomitant Therapy 30](#_Toc62640083)

[6.10.1. Prohibited Medications 31](#_Toc62640084)

[6.10.2. Precautionary Medications 32](#_Toc62640085)

[6.11. Hospitalization 32](#_Toc62640086)

[6.12. Treatment after the End of the Study 32](#_Toc62640087)

[7. Discontinuation/Withdrawal Criteria and Study Completion 32](#_Toc62640088)

[7.1. Discontinuation of Study Intervention 32](#_Toc62640089)

[7.2. Withdrawal from the Study 33](#_Toc62640090)

[7.3. Lost to Follow-up 33](#_Toc62640091)

[7.4. Study Completion 34](#_Toc62640092)

[8. Study Encounters 34](#_Toc62640093)

[8.1. Screening Process and Procedures 34](#_Toc62640094)

[9. Study Assessments and Procedures 35](#_Toc62640095)

[9.1. Day 1 (first day of study) 35](#_Toc62640096)

[9.2. Procedures at Visits from Day 4 through Day 28 36](#_Toc62640097)

[9.3. Procedures at Visit Day 35 37](#_Toc62640098)

[9.4. Efficacy Assessments 37](#_Toc62640099)

[9.4.1. Nasal Sampling 37](#_Toc62640100)

[9.5. Safety Assessments 37](#_Toc62640101)

[9.5.1. Adverse Event Definition 38](#_Toc62640102)

[9.5.2. Adverse Events of Special Interest Definition 38](#_Toc62640103)

[9.5.3. Serious Adverse Event Definition 38](#_Toc62640104)

[9.5.4. Toxicity Grading 39](#_Toc62640105)

[9.5.5. Study Intervention-Related AEs and SAEs 39](#_Toc62640106)

[9.6. Assessment of COVID-19 Signs and Symptoms 39](#_Toc62640107)

[9.7. Overdose of Study Intervention 41](#_Toc62640108)

[9.8. Pregnancy 41](#_Toc62640109)

[9.8.1. Contraceptive Guidance 42](#_Toc62640110)

[9.9. Biohazard Containment 42](#_Toc62640111)

[10. Statistical Considerations 43](#_Toc62640112)

[10.1. Sample Size Determination 43](#_Toc62640113)

[10.2. Statistical Hypothesis 43](#_Toc62640114)

[10.3. Populations for Analyses 43](#_Toc62640115)

[10.3.1. Assigning Shared Controls across Multiple Intervention-Specific Analysis Populations 44](#_Toc62640116)

[10.4. Primary and Key Secondary Endpoints 46](#_Toc62640117)

[10.5. Statistical Methods 47](#_Toc62640118)

[10.5.1. Efficacy Analyses 47](#_Toc62640119)

[10.5.2. Safety Analyses 48](#_Toc62640120)

[10.6. Interim Analysis 48](#_Toc62640121)

[11. Operational Considerations 48](#_Toc62640122)

[11.1. Independent Data Monitoring Committee 48](#_Toc62640123)

[11.2. Informed Consent Process 49](#_Toc62640124)

[11.2.1. Informed Consent Forms 49](#_Toc62640125)

[11.3. Data Protection 50](#_Toc62640126)

[11.4. Sample Retention and Future Use of Collected Biospecimen 50](#_Toc62640127)

[11.5. Dissemination of Clinical Study Data 50](#_Toc62640128)

[11.6. Data Quality Assurance 51](#_Toc62640129)

[11.7. Source Documents 51](#_Toc62640130)

[11.8. Record Retention and Future Use of Medical Data 51](#_Toc62640131)

[11.9. Study Closure 52](#_Toc62640132)

[11.10. Publication Policy 52](#_Toc62640133)

[12. References 53](#_Toc62640134)

[13. Version History 56](#_Toc62640135)

[13.1. Amendment 1 dated 09 Sept 2020 56](#_Toc62640136)

[13.2. Amendment 2 dated 27 January 2021 63](#_Toc62640137)

List of Tables, Figures and Appendix

[Table 1: Schedule of Activities 11](#_Toc62640147)

[Table 2: Bleeding events in MAGELLAN study 16](#_Toc62640148)

[Table 3: Objectives and Endpoints 19](#_Toc62640149)

[Table 4: Pre-screening Schedule of Activities 23](#_Toc62640150)

[Table 5: Rivaroxaban prohibited medications/foods/supplements 31](#_Toc62640151)

[Table 6: COVID-19 Signs and Symptoms to be Recorded in the CRF/eCRF 40](#_Toc62640152)

[Table 7: Gates MRI COVID-19 Ordinal Scale Clinical Endpoint Definitions 40](#_Toc62640153)

[Table 8: WHO Ordinal Scale for Assessment of Clinical Status of COVID-19 Patients 41](#_Toc62640154)

[Table 9: Populations for Analysis 43](#_Toc62640155)

[Figure 1: Study Screening Process 9](#_Toc62640156)

[Figure 2: Pre-Screening for the Study 21](#_Toc62640157)

[Figure 3: Randomization strategy for sites where 3 interventions are being studied 45](#_Toc62640158)

[Figure 4: Randomization strategy for sites where 2 interventions are being studied 46](#_Toc62640159)

[Figure 5: Randomization strategy for sites where only 1 intervention is being studied 46](#_Toc62640160)

Appendix 1: Platform Core Protocol

List of Abbreviations and Terms

| **Term** | **Definition** |
| --- | --- |
| AE | Adverse Event |
| AESI | Adverse Event of Special Interest |
| AUC | Area Under the Curve |
| BMI | Body Mass Index |
| CI | Confidence Interval |
| Cmax | Maximum Concentration |
| COVID-19 | Coronavirus Disease 2019 |
| CRF/eCRF | (Electronic) Case Report Form |
| CRO | Contract research organization |
| DOAC | Direct Oral Anticoagulant |
| DVT | Deep Vein Thrombosis |
| Eligible | Qualified for enrollment into the study based upon adherence to inclusion/exclusion criteria |
| Gates MRI | Bill & Melinda Gates Medical Research Institute |
| GCP | Good Clinical Practices |
| IDMC | Independent Data Monitoring Committee |
| IEC | Independent Ethics Committee |
| IP | Investigational Product |
| IRB | Institutional Review Board |
| ITT | Intention to Treat |
| LRTI | Lower Respiratory Tract Infection |
| mITT | Modified Intention to Treat |
| Participant(s) | Term used throughout the protocol to denote the enrolled individual(s) |
| PD | Pharmacodynamic |
| PE | Pulmonary embolism |
| P-gp | P-glycoprotein |
| PK | Pharmacokinetic |
| PP | Per Protocol |
| SAE | Serious Adverse Event |
| SAP | Statistical Analysis Plan |
| SARS-CoV-2 | Severe Acute Respiratory Syndrome Coronavirus 2 |
| SoA | Schedule of Activities |
| US | United States |
| VTE | Venous Thromboembolism |
| WHO | World Health Organization |

#

# Synopsis

**Protocol Title**: A randomized, controlled, Phase 2b study to evaluate safety and efficacy of rivaroxaban **(**Xarelto®) for high risk people with mild COVID-19

**Short Title:** A randomized, controlled, Phase 2b trial to evaluate safety and efficacy of rivaroxaban in high risk people

**Study Intervention and Rationale:**

Rivaroxaban is an approved, direct oral anticoagulant (DOAC) factor Xa inhibitor that is indicated for treatment and prophylaxis of thromboembolism. SARS-CoV-2 infection can trigger systemic inflammatory response, hypercoagulability, and a prothrombotic state in people whose coronavirus disease 2019 (COVID-19) progresses to severe disease [Bikdeli 2020].

Thromboembolic events are common in patients hospitalized with COVID-19. High levels of blood-clotting parameters are associated with severe COVID-19 and mortality [Wu 2020]. Widespread thrombosis with microangiopathy and alveolar capillary thrombi are prevalent in patients who die from COVID-19 [Ackermann 2020]. Anticoagulant therapy may decrease mortality in patients with severe COVID-19 with associated coagulopathy [Tang 2020]. There is evidence to suggest that abnormal coagulation and thrombosis are occurring in people with non-severe COVID-19 and may place them at greater risk of severe disease [Fu 2020, Micco 2020, Nauka 2020].

Rivaroxaban is selected as a candidate treatment modality to reduce disease progression in people with mild COVID-19 who are at high risk for moderate or severe disease due to age, body mass index (BMI) and comorbidities, many of which are also thrombotic risks.

Our hypothesis is that rivaroxaban will reduce COVID-19 progression from mild to moderate or severe disease category or higher.

Rivaroxaban is a once-daily, oral medication with a well characterized safety profile, with no cold chain requirement. If proven to be efficacious in preventing COVID-19 progression, it has global scalability potential.

**Primary purpose:** To assess the safety and clinical efficacy of rivaroxaban in reducing the proportion of participants who have progressed to moderate or severe disease category or higher in people with mild COVID-19 who are at increased risk of disease progression.

**Design**: This is a randomized, Phase 2b, controlled study. People who are symptomatic and have, or are suspected to have, COVID-19, and are at increased risk for COVID-19 progression based on age, BMI or comorbidity will be enrolled (refer to Section 5.5 for eligibility criteria).

This study is performed as part of the overall COVID-19 platform core protocol (refer to **Appendix 1**) that intends to provide data to:

1. support decision and development plans for Phase 3 in consultation with key stakeholders.
2. support recommendations by regulators and policy makers for use in treating COVID-19.

**Interventional groups, randomization, and dosing**: Participants will be randomly assigned, in a 1:1 allocation, to either rivaroxaban or placebo, in parallel. Randomization will be stratified by site and by number of days since onset of symptoms (<6 days vs. ≥6 days).

Participants randomized to the rivaroxaban group will receive 10 mg (1 tablet) Xarelto® orally daily for 21 days and participants randomized to the placebo group will receive (1 tablet) placebo-equivalent orally daily for 21 days.

**Number of Participants and Population:**

The screening process is summarized in Figure 1.

There is an opportunity for high risk participants with COVID-19 who are currently asymptomatic to be pre-screened for this study (refer to Section 5.4).

Figure 1: Study Screening Process

**Sample Size:** The total number of participants enrolled will be approximately 600: 300 per group. If the true control rate for disease progression is 30%, there is approximately 80% power to detect a treatment effect of 35%.

**Total duration of study participation:** The study duration is 35 days, (Day 1 through Day 35) after the screening process, for each participant.

**Study sites**: Participants from multiple sites in multiple countries may be included.

**Masking:** Every effort will be made to mask the assigned treatment groups. The laboratories performing the SARS-CoV-2 diagnostic testing will be blinded to the treatment groups. The participants will not be told of the assigned treatment groups. Although the participants will not be informed that active and control treatment do not look identical, due to features such as embossing on the rivaroxaban tablets, the participants may attempt to identify the intervention they receive via computer image search. The active and placebo tablets will not be identical in appearance; but will be similar in size, shape and color. Moreover, the bottles will be identical.

The study staff evaluating the participants will not be given the assigned treatment groups, but could possibly find out if the participant’s tablets become visible to them. Such occurrences will be documented. It is recognized that there is some risk of bias related to the outcome assessments by the participant and/or study staff, particularly for subjective measurements.

**Safety and efficacy monitoring**: An independent data monitoring committee (IDMC) will be convened for this study (Section 11.1.1). Unblinded safety data review by the IDMC will occur monthly, and ad hoc as necessary. The IDMC will also review unblinded efficacy data at a pre-specified interim analysis. Pausing guidelines will apply (refer to Section 6.6).

**Adverse events (**AEs): All AEs must be recorded on the eCRFs if any of the following criteria have been met:

- AEs meeting SAE definition
- AEs leading to discontinuation of study intervention
- Grade 3 and grade 4 AEs
- Adverse events of special interest (AESI) including major bleeding (refer to Section 6.4.1) and severe hypersensitivity to rivaroxaban.

AEs will be recorded in the eCRF from the time of informed consent through Day 35 (14 days after the last dose of investigational product or IP).

**Analysis:** The primary analysis will occur after all participants complete Day 35. There will be ***1*** interim ***analysis*** to assess ***futility.*** The interim analysis will occur after the first approximately 200 participants complete Day 28.

If the trial does not cross the pre-specified boundaries to declare futility at the interim ***analysis***, a final efficacy and safety analysis will occur after all participants complete Day 35.

# Study Schedule of Procedures

Table 1: Schedule of Activities

| **Visits** | **Screening**  **(≤5 days of Day 1)** | **Day**  **1** | **Day**  **4** | **Day**  **6** | **Day**  **8** | **Day**  **10** | **Day**  **12** | **Day**  **14** | **Day 18** | **Day 21** | **Day 24** | **Day**  **28** | **Day 35** |
| --- | --- | --- | --- | --- | --- | --- | --- | --- | --- | --- | --- | --- | --- |
| Obtain consent | X |  |  |  |  |  |  |  |  |  |  |  |  |
| Demographics, past and current medical history including known pregnancy/lactation status, and medication history | X |  |  |  |  |  |  |  |  |  |  |  |  |
| Lab-confirmed SARS-CoV-2 positive diagnostic testing^1^ | X |  |  |  |  |  |  |  |  |  |  |  |  |
| Inclusion and exclusion criteria | X |  |  |  |  |  |  |  |  |  |  |  |  |
| Concomitant medications | X | X | X | X | X | X | X | X | X | X | X | X | X |
| Randomization^2^ | X |  |  |  |  |  |  |  |  |  |  |  |  |
| Study intervention dose  (Day 1 Day 21) and record adherence to dosing |  | X | X | X | X | X | X | X | X | X |  |  |  |
| Clinical status assessment using ordinal scales for Gates MRI and WHO |  | X | X | X | X | X | X | X | X | X | X | X |  |
| COVID-19 signs and symptoms, temperature, oxygen saturation |  | X | X | X | X | X | X | X | X | X | X | X |  |
| AEs assessment | X | X | X | X | X | X | X | X | X | X | X | X | X |
| Bleeding events and severity assessment |  | X | X | X | X | X | X | X | X | X | X | X | X |
| Self-collection of nasal or oropharynx SARS-CoV-2 sample for diagnostic testing^3^ | (X)^1^ | X | X |  | X |  |  | X |  | X |  | X |  |

Note that visits will preferably take place remotely (refer to Section 8).

SARS-CoV-2= Severe acute respiratory syndrome coronavirus 2

Gates MRI= Bill & Melinda Gates Medical Research Institute

WHO = World Health Organization

^1^ If there is documented positive SARS-CoV-2 diagnostic testing performed with a sample collected ≤10 days prior to screening, the test does not need to be performed. If no documentation, the potential participant will be asked to collect a nasal or oropharynx swab sample to send for testing.

^2^ If documentation of a previous positive SARS-CoV-2 test is not available and a test is performed at screening, randomization will occur after a positive result is obtained.

^3^ Participant will be asked to collect a sample per kit instructions. Discontinue collection during hospitalization and/or per physician’s discretion when two consecutive negative results are available at any time during the study.

Note that an unscheduled visit may be required. Additional collection of a nasal swab sample may be performed based on the clinical judgement of the PI.

# Background

## Severe Acute Respiratory Syndrome Coronavirus-2

Severe acute respiratory syndrome coronavirus 2 (SARS-CoV-2) is a novel beta coronavirus of zoonotic origin, similar to SARS-CoV and Middle East respiratory syndrome coronavirus (MERS-CoV). The case fatality rate for SARS-CoV-2 varies between age groups and people with or without risks as well as regions of the world. Based on current evidence, the overall case fatality risk is lower than that of SARS-CoV (~10%) and MERS‑CoV (~40%) [Chen 2020]. However, SARS-CoV-2 has potentially higher transmissibility (R0: 1.4 to 5.5) than both SARS-CoV (R0: 2 to 5) and MERS-CoV (R0: <1).

Our understanding of the viral pathogenesis of SARS-CoV-2 remains limited. However, it appears that the virus cell entry depends on the binding of the viral spike (S) proteins to cellular receptors and on S protein priming by host cell proteases. SARS-CoV-2, like SARS-CoV, uses the angiotensin converting enzyme 2 (ACE2) receptor on pulmonary epithelial cells for entry and the transmembrane serine protease 2 for S protein priming [Hoffmann 2020]. The receptor binding domain of lineage B beta coronaviruses S protein is a single, continuous domain that contains all of the structural information necessary to interact with the host receptor. Fusion is mediated at the cell membrane, delivering the viral nucleocapsid inside the cell for subsequent replication. ACE2 expression is found in the lung epithelial cells, vascular endothelium, renal tubular epithelium, and epithelia of the small intestine. Viral shedding has been localized primarily to respiratory droplets and fecal samples [Zhu 2020].

### SARS-CoV-2 Epidemiology and Disease

Since the first reported case in December 2019, SARS-CoV-2 infection has spread globally [Chen 2020, Huang 2020, Zhu 2020]. WHO declared COVID-19 a Public Health Emergency of International Concern on 30 January 2020 [WHOa 2020] and a pandemic on 11 March 2020. United States declared a national emergency on 13 March 2020 [WHPR 2020]. As of September 2020, approximately 26 million confirmed cases were reported globally, with over 859,000 deaths [JHU 2020].

SARS-CoV-2 infection is estimated to cause mild to moderate disease in about 80% of people but can cause severe lower respiratory tract infection (LRTI) in approximately 15-20%, particularly among those in high-risk groups, defined by advanced age (≥65 years), presence of comorbidities (e.g., cardiopulmonary disease, diabetes mellitus) or obesity [CDC-2020].

Progression to LRTI appears to frequently result in hospitalization for supplemental oxygen therapy and may lead to need for ventilator respiratory support and ultimately death. Given the rapid spread of the SARS-CoV-2 pandemic, interventions that avert adverse outcomes and accelerate symptom resolution are needed. In addition, prolonged viral shedding has been noted after infection; therapeutic strategies that can effectively reduce viral shedding, and potentially onward transmission, have the potential to shift the trajectory of the pandemic. Intervening early in an outpatient setting before disease progression occurs is important to mitigate human suffering and healthcare utilization.

Most deaths and severe pulmonary and extra-pulmonary diseases have occurred particularly in adults over 65 years of age and in persons with underlying pulmonary or cardiac comorbidities, obesity or diabetes. In healthy adults, including pregnant women, it can cause a febrile, self-limited pneumonia. Severe disease is seen less frequently in children and younger adults [Beigel 2020, Cai 2020].

Manifestations of COVID‑19 in persons with comorbidities, such as chronic malaria, tuberculosis, or HIV infection, are not well-characterized. Nevertheless, the burden of this pandemic to the global health and economic systems is substantial.

As with many infectious epidemics, household contacts, first responders, caregivers, and medical personnel attending to persons with COVID-19 are at high risk of infection. The incubation time requires 10-14 days of quarantine for exposed individuals not wearing personal protective equipment [Linton 2020], and on 03 March 2020, WHO declared a global shortage of personal protective equipment, leaving doctors, nurses, and other frontline workers dangerously ill-equipped to care for COVID-19 participants [WHO(b) 2020]. Extensive absences from the care network and health system will degrade the ability to care not only for those with COVID-19 but also for routine healthcare issues as well. At the height of local epidemic, the health care system becomes overburdened with individuals with respiratory illness. To date, rigorous self-isolation and lockdown have been required to contain the SARS-CoV-2 outbreak, leaving entire societies to abruptly stop normal life. Interventions are urgently needed to stop viral spread and to decrease the morbidity and mortality caused by the infection. The ability to stop viral replication to prevent transmission of the virus and to prevent LRTI, which is associated with need for hospitalization and possibly mechanical ventilatory support, will be of benefit to the individual, the hospital system, and the health of the public.

### Therapeutic Approaches for COVID-19

As of September 2020, no standard of care for treatment of outpatients with COVID-19 has been established. Already-approved and available medications are ideal for immediate evaluation for SARS-CoV-2 treatment.

There is an urgent need for controlled clinical studies for early treatment in people with mild COVID-19 to guide both policy makers and other clinical researchers on prioritized drugs, including a path toward future evaluations as data becomes available.

This study is performed as part of an adaptive, randomized platform core protocol for treatment of early and mildly symptomatic individuals in out-patient settings with SARS-CoV-2 infection (Appendix 1). The platform protocol includes the contents of the adaptive, platform trial (e.g., background information and rationale, the trial design and conduct). The platform design is intended to support investigations of a broad array of therapeutic approaches such as antiviral therapies, host-directed therapies, monoclonal antibodies, and hyperimmunoglobulin.

### SARS-CoV-2 Immunothrombosis and Thromboembolic Events

SARS-CoV-2 infection may be a trigger for venous thromboembolism (VTE), and several pathogenetic mechanisms are involved, including endothelial dysfunction, characterized by increased levels of von Willebrand factor; systemic inflammation, by Toll-like receptor activation; and a procoagulatory state, by tissue factor pathway activation. Infection with SARS-CoV-2 in severe patients progresses from a viral infection to a complex inflammatory condition characterized by sudden decline of patients due to hyperinflammation and thrombotic events [Bikdeli 2020]. In the absence of severe immune complications, SARS-CoV-2 infection is self-limiting and typically mild.

Hemostatic abnormalities including elevated levels of D-dimer and other thrombotic markers (e.g., platelets) are frequently seen at hospital admission for COVID-19; levels have been linked to disease severity in multiple studies. Increased D-dimer concentrations (indicating pulmonary vascular bed thrombosis with fibrinolysis) and elevated cardiac enzyme concentrations (indicating emergent ventricular stress induced by pulmonary hypertension) are key early features of severe pulmonary intravascular coagulopathy related to COVID-19 [McGonagle 2020].

Thrombosis (mostly venous) and abnormal coagulation are seen in at least 30% of ICU patients. Pulmonary embolism (PE), deep vein thrombosis (DVT), ischemic stroke, myocardial infarction or systemic arterial embolism have been observed [2020]. DVT has also been reported in mild COVID cases [Nauka 2020].

Autopsies of patients who died of COVID-19 revealed PE and DVT (including in people not suspected to have DVT before death). Histomorphological findings were distinctive vascular features with severe endothelial injury, and diffuse alveolar capillary microthrombi that was 9 times more prevalent in patients who died from COVID-19 than influenza [Ackermann 2020, Wichmann 2003]. Microthrombi in the pulmonary vasculature may explain the rapid deterioration and pulmonary collapse observed in patients who suddenly progress to acute respiratory distress syndrome.

Hospitalized patients acutely ill from medical conditions such as infections and pneumonia are at increased risk of VTE, for whom anticoagulant could be indicated. Risk assessment modeling studies are reporting 40% VTE risk among hospitalized COVID-19 patients [Wang 2020]. The World Health Organization (WHO) interim guidance recommends in-hospital prophylactic daily low-molecular weight heparin or twice daily subcutaneous unfractionated heparin [WHO(c) 2020]. Use of DOACs like rivaroxaban in the hospitalized population is limited by drug interactions with antiviral and other agents for treatment of COVID-19 or its complications. However, rivaroxaban may benefit high risk people with non-severe disease in the outpatient setting who are less likely to need those treatments with significant drug interaction with rivaroxaban.

## Rivaroxaban

Xarelto® (rivaroxaban) is manufactured by Janssen Pharmaceutical Companies of Johnson & Johnson and was first approved by the FDA in 2011. Xarelto® is currently approved for the following indications [Xarelto® 2020]:

- reduce risk of stroke and systemic embolism in nonvalvular atrial fibrillation (15 or 20mg daily)
- treatment of DVT (20mg daily after initial treatment of 15mg twice daily for 21 days)
- treatment of PE (20mg daily after initial treatment of 15mg twice daily for 21 days)
- reduce risk of recurrence of DVT or PE (10mg daily after at least 6 months of standard anticoagulation treatment)
- prophylaxis of DVT, which may lead to PE in patients undergoing knee or hip replacement surgery (10mg once daily)
- prophylaxis of VTE in acutely ill medical patients (10mg once daily during hospitalization and 31-39 days after hospital discharge)
- reduce risk of major cardiovascular events in patients with chronic coronary artery disease or peripheral artery disease (2.5mg twice daily in combination with daily aspirin).

During clinical development for these approved indications, Xarelto® was evaluated in approximately 30,000 patients. The contraindications for Xarelto are active pathological bleeding and severe hypersensitivity reaction to Xarelto®. They will be followed as AESIs in this study.

The use of rivaroxaban for mild COVID-19 will follow usage guidelines for the prophylaxis of VTE in acutely ill medical patients who are at risk for thromboembolic complications due to moderate or severe restricted mobility and other risk factors for VTE and not at high risk of bleeding. The recommended dosage for this use is 10mg once daily, in hospital and after hospital discharge, for a total recommended duration of 31 to 39 days. The dosing in this study of 10mg daily for 21 days is in line with the indicated dose. Renal considerations for dosage include CrCl≥15mL/min. This study will exclude people with severe chronic kidney disease (stage IV or receiving dialysis).

According to the package insert, Xarelto® increases the risk of bleeding, and, although rare, can cause serious or fatal bleeding. Concomitant use of other drugs that impair hemostasis increases the risk of bleeding. Xarelto® is not for use for primary VTE prophylaxis in these hospitalized, acutely ill medical patients at high risk of bleeding.

The efficacy and safety of Xarelto® for prophylaxis of VTE was evaluated in a multicenter, randomized, parallel group efficacy and safety study for the prevention of venous thromboembolism in hospitalized medically ill patients comparing rivaroxaban with enoxaparin, the MAGELLAN study, [NCT00571649]). MAGELLAN was a multicenter, randomized, double-blind, parallel-group efficacy and safety study comparing Xarelto® to enoxaparin, in the prevention of VTE in hospitalized acutely ill medical patients during the in-hospital and post-hospital discharge period. Eligible patients included adults who were at least 40 years of age, hospitalized for an acute medical illness, at risk of VTE due to moderate or severe immobility, and had additional risk factors for VTE. Patients were randomized to receive either Xarelto® 10 mg once daily for 35 ±4 days starting in hospital and continuing post hospital discharge (n=4050) or enoxaparin 40 mg once daily for 10 ±4 days starting in hospital followed by placebo post-discharge (n=4051) [Cohen 2013].

Approximately 67% were 65 years and over and about 37% were >75 years. A principal safety outcome event occurred in 111 of 3997 patients (2.8%) in the rivaroxaban group [Cohen 2013]. Safety analysis of bleeding is shown in Table 2.

Over the total study period, alanine aminotransferase elevations greater than three times the upper limit of the normal range with a concurrent elevation in the bilirubin level that was greater than two times the upper limit of the normal range occurred in 7 of 3364 patients (0.2%) in the rivaroxaban group.

Table 2: Bleeding events in MAGELLAN study

| **Events** | **XARELTO 10 mg**  **N=3218**  **n (%)** |
| --- | --- |
| Major bleeding ^‡†^ | 22 (0.7) |
| Critical site bleeding | 7 (0.2) |
| Fatal bleeding § | 3 (<0.1) |
| Clinically relevant non-major bleeding events | 93 (2.9) |

Safety Analysis Set- On Treatment Plus 2 Days

† Major bleeding events within each subcategory were counted once per patient, but patients may have contributed events to multiple subcategories. These events occurred during treatment or within 2 days of stopping treatment.

‡ Defined as clinically overt bleeding associated with a drop in hemoglobin of ≥2 g/dL, a transfusion of ≥2 units of packed red blood cells or whole blood, bleeding at a critical site, or with a fatal outcome.

§ Fatal bleeding is adjudicated death with the primary cause of death from bleeding.

### Use in Specific Populations

***Pregnancy***: Xarelto® dosing in pregnancy has not been studied. The limited available data on Xarelto® in pregnant women are insufficient to inform a drug-associated risk of adverse developmental outcomes. There is a risk of pregnancy-related hemorrhage due to the potential for obstetric hemorrhage and/or emergent delivery. Rivaroxaban should only be used if benefits outweigh risk. Pregnancy is a known risk factor for VTE but not for COVID-19. This study will exclude pregnant women.

***Lactation***: There is insufficient data to determine the effect of rivaroxaban on the breastfed child or on milk production. This study will exclude lactating women.

### Rationale for the Use of Rivaroxaban for Treatment of Mild COVID-19

COVID-19 progression requiring hospitalization generally occurs from the second week of illness and is characterized by intense systemic inflammation and hypercoagulability. People who present with mild COVID-19 during the first week are likely transitioning from the active viral replication phase to the inflammatory phase of the disease [Siddiqi 2020]. This offers a unique opportunity to intervene early and prevent further progression of disease by evaluating a thromboprophylactic drug, rivaroxaban, in people at risk for COVID-19 progression.

There is precedent for the use of approved drugs with a well-characterized and favorable safety profile to be considered for treatment of COVID-19. For example, remdesivir, although not approved as a treatment for COVID-19, has received FDA Emergency Use Authorization for hospitalized COVID-19 patients [FDA 2020].

#### Rationale for a Limit of 7 Days of Mild COVID-19 Symptoms Prior to Randomization

The incubation period from exposure to early manifestations of COVID-19 ranges from 2 to 14 days. During the first 3 days of illness, COVID-19 symptoms may be non-specific and include cough, fever, headache, lethargy, and/or diarrhea. During the next 3-5 days some patients develop worsened symptoms but some patients may show signs of recovery. The second week of illness, Days 8-12, is when progression to signs and symptoms indicative of a hyperinflammatory phase is more likely to occur. These may include dyspnea, abnormal imaging of the lungs, respiratory failure, ARDS, and/or cytokine storm syndrome. In a large series of 410 patients with COVID-19 hospitalized in Italy [Ciceri 2020], the median (IQR) days from COVID-19 symptoms onset to hospitalization was 8 days (5-11) days. Approximately 50% of these patients were aged >65 years and approximately 60% had one or more comorbidities.

The primary endpoint of this study is the time to progression of COVID-19 from Scale 2 to Scale 3 or higher – a situation that would likely require hospitalization of the participant. The data from the cited Italian study can inform the choice of the interval between onset of symptoms and the expected time of initiation of study drug therapy. Because the interval from onset of symptoms to randomization should be no more than 7 days resulting in the worst-case scenario of an interval of around 9 days from onset of symptoms to first dose of study treatment (assuming two days for shipment of IP and supplies). Nine days falls within the IQR reported by Ciceri, et al [Ciceri 2020].

### Benefit/Risk Assessment

The elderly (≥65 years of age) and those with medical comorbidities (e.g., cardiopulmonary disease, diabetes mellitus) or obesity are at high risk of poor outcomes from COVID-19 [Chen 2020, Huang 2020, Richardson 2020, Zhu 2020]. In a large case series of COVID-19 patients in New York City, the risks for hospitalization were 10-times and 40-times higher in people 65 to 74 years old and over 75 years old compared to those younger than 45 years old. People with high BMI and comorbidities are 2 to 5 times more likely to be hospitalized compared to those without risk. Similarly, mortality rates are higher in people with these risk factors [Petrilli 2020].

It is estimated that 20% to 30% of people with risk factors will progress to moderate or severe COVID-19 [Williams 2020]. The benefit-to-risk ratio for testing treatment is favorable in this population.

#### Benefit/Risk Assessment of Rivaroxaban

Without any approved treatment for outpatients with COVID-19, there is a favorable benefit-risk ratio for using rivaroxaban in the target population at increased risk for progressing to moderate and severe COVID-19.

Rivaroxaban is an approved drug with a well-characterized and favorable safety profile.

The dose selected for this study (10mg/day) is the same as the licensed prophylactic dose for VTE in acutely ill patients and fits the purpose of preventing thromboembolic events in people with acute COVID-19 illness. This study will exclude people who may be at increased risk for adverse events or drug interactions due to rivaroxaban as well as those with severe renal and liver impairment, and pregnant and breastfeeding women.

### Rivaroxaban Mechanism of Action

Rivaroxaban is a selective inhibitor of factor Xa, the activated form of coagulation factor X. It does not require a cofactor (such as anti-thrombin III) for activity. Rivaroxaban inhibits free factor Xa and prothrombinase activity. Rivaroxaban has no direct effect on platelet aggregation, but indirectly inhibits platelet aggregation induced by thrombin. By inhibiting factor Xa, rivaroxaban decreases thrombin generation.

### Rivaroxaban Pharmacokinetics

***Absorption***

The absolute bioavailability of rivaroxaban is dose-dependent. For the 2.5 mg and 10 mg dose, it is estimated to be 80% to 100% and is not affected by food. Rivaroxaban 2.5 mg and 10 mg tablets can be taken with or without food. For the 20 mg dose in the fasted state, the absolute bioavailability is approximately 66%.

Coadministration of rivaroxaban with food increases the bioavailability of the 20 mg dose (mean area under the curve (AUC) and maximum concentrations (Cmax), increasing by 39% and 76% respectively, with food).

The Cmax of rivaroxaban appears 2 to 4 hours after tablet intake.

***Distribution***

Plasma protein binding of rivaroxaban in human plasma is approximately 92% to 95%, with albumin being the main binding component. The steady-state volume of distribution in healthy subjects is approximately 50 L.

***Metabolism***

Approximately 51% of an orally administered [^14^C]-rivaroxaban dose was recovered as inactive metabolites in urine (30%) and feces (21%). Oxidative degradation catalyzed by CYP3A4/5 and CYP2J2 and hydrolysis are the major sites of biotransformation. Unchanged rivaroxaban was the predominant moiety in plasma with no major or active circulating metabolites.

***Excretion***

In a Phase 1 study, following the administration of [^14^C]-rivaroxaban, approximately one third (36%) was recovered as unchanged drug in the urine and 7% was recovered as unchanged drug in feces. Unchanged drug is excreted into urine, mainly via active tubular secretion and to a lesser extent via glomerular filtration (approximate 5:1 ratio). Rivaroxaban is a substrate of the efflux transporter proteins, P-glycoprotein (P-gp) and ABCG2 (also abbreviated Bcrp). Rivaroxaban’s affinity for influx transporter proteins is unknown.

Rivaroxaban is a low-clearance drug, with a systemic clearance of approximately 10 L/hr in healthy volunteers following intravenous administration. The terminal elimination half-life of rivaroxaban is 5 to 9 hours in healthy subjects aged 20 to 45 years, and 11 to 13 hours in the elder participants aged 60 to 76 years

Pharmacokinetics (PK) and pharmacodynamics (PD) of rivaroxaban were not influenced by gender, but by race (higher exposure in healthy Japanese) and severe renal and liver impairment (which are exclusion criteria in this study). Please refer to package insert for more information.

Refer to Section 6.4 for dose modification and toxicity management.

# Objectives and Endpoints

Objectives and endpoints are provided in Table 3.

Table 3: Objectives and Endpoints

| **Objectives** | **Endpoints** |
| --- | --- |
| ***Primary*** | |
| To characterize safety of study intervention | Through Day 35   - Frequencies of grade 3 AEs and grade 4 AEs - AEs resulting in study intervention discontinuation - All SAEs |
| To assess efficacy of study intervention | - Proportion of participants who progress to moderate or severe disease category or higher (Gates MRI ordinal scale ≥3) through Day 28 |
| ***Secondary*** | |
| - To assess clinical efficacy of study intervention | - Time to disease resolution, defined as BOTH viral clearance (two consecutive negative diagnostic tests) and symptoms resolution (new onset COVID-19 symptoms resolved, and pre-existing symptoms returned to baseline*) through Day 28 - Time to disease resolution, defined as symptoms resolution only (new onset COVID-19 symptoms resolved, and pre-existing symptoms returned to baseline*) through Day 28 |
|  | - Proportion of participants who progress to moderate or severe disease category or higher (Gates MRI ordinal scale ≥3) at Days 8, 14 and 21 - Proportion of participants who achieve disease resolution at Days 8, 14, 21 and 28 - Gates MRI scale score at Days 8, 14, 21 and 28 - WHO ordinal scale score at Days 8, 14, 21 and 28 - Incidence and number of days of hospitalization at Days 8, 14, 21 and 28 |
| ***Exploratory objectives*** | |
| To assess virological efficacy | - Quantity (and change from baseline) of SARS-CoV-2 virus at Days 8, 14, 21 and 28 |
| To assess viral sequence | - Phylogenetic relationships of SARS-CoV-2 viruses sequenced from positive nasal swab samples |

*Baseline refers to health status prior to contracting COVID-19.

# Study Design

## Overall Design

This is a randomized, controlled study for treatment of mild COVID-19 in people at high risk for disease progression.

Mild COVID-19 is determined by Gates MRI mild COVID-19 clinical endpoint definition (refer to Table 7).

High risk is defined based on age, BMI or presence of a comorbidity.

***Primary purpose***: To assess the safety and clinical efficacy of rivaroxaban in reducing the proportion of participants who have progressed to moderate or severe disease category or higher in people with mild COVID-19 who are at increased risk of disease progression.

***Interventional model:*** Participants will be randomized 1:1 to either the rivaroxaban or the placebo group, in parallel for the duration of the study. Randomization will be stratified by site and by number of days since onset of symptoms (<6 days vs. ≥6 days).

***Intervention groups***:

- Study intervention group: Rivaroxaban, 10 mg (1 tablet) orally daily for 21 days.
- Control group: placebo-equivalent (multi-vitamin supplement) 1 tablet orally daily for 21 days.

***Masking***: Every effort will be made to mask the assigned treatment groups. Participants and study staff evaluating them will not be told of the assigned treatment groups. The laboratories performing the SARS-CoV-2 diagnostic testing will be blinded. Refer to Section 6.7 for details.

***Study visits:*** Every effort will be made to conduct the study protocol visits virtually (defined in Section 8) with the study participants. A web-based video conferencing tool will be employed to allow for virtual (remote) interactions to take place between the study staff and the participant, for screening and all study visits.

***Safety and efficacy monitoring***: Refer to Section 6.5 for safety monitoring. An IDMC will be convened for this study for safety and efficacy with expertise in COVID-19 or respiratory viruses and emerging epidemics as well as biostatistics. Refer to Section 11.1.1 for details regarding the IDMC.

## Study Population

The total number of participants enrolled will be approximately 600, with 300 participants per group.

Refer to Section 5.5.1 for eligibility criteria.

## Recruitment

Study screening processes will occur for participants at high risk for severe COVID-19 who are mildly symptomatic and have or suspected to have COVID-19 (refer to Section 5.4 for pre-screening and to Section 5.5 for screening). There is an opportunity for high risk participants with COVID-19 who are currently asymptomatic to be pre-screened for this study; however, pre-screening is not a pre-requisite to entering the study.

## Pre-Screening for the Study

The purpose of pre-screening is to facilitate enrollment of participants with mild COVID-19 into the study as soon as they become symptomatic. Persons who are in the high risk category for COVID-19 progression and are asymptomatic may enroll in the pre-screening phase provided that they are willing to be contacted by the study team and are interested in enrolling in the COVID-19 treatment trial if they become symptomatic. They must have a confirmed positive SARS-CoV-2 diagnostic testing within the past 7 days at Day 1 of pre-screening.

Participants who do not yet have a documented positive SARS-CoV-2 diagnostic test may have the testing done at the pre-screening eligibility check visit provided that they have had a high risk **exposure** to SARS-CoV-2 within the past 14 days.

High risk exposure is defined as:

1. being an immediate household contact of a confirmed COVID-19 case and/or
2. having occupational exposure to a confirmed COVID-19 case (for example, healthcare worker or first responder).

Figure 2: Pre-Screening for the Study

### Pre-Screening Inclusion Criteria

**Age**

1. ≥18 years of age at the time of informed consent

**Type of Participant**

1. Participants must be at high-risk for COVID-19 progression by fulfilling at least **one** of the following criteria at screening:

- Age ≥65 years
- Presence of a chronic disease requiring daily treatment (such as diabetes, lung disease, heart disease, hypertension or cancer)
- Self-reported obesity
- Any other high-risk criteria per national guidelines.

**COVID-19 characteristics**

1. Documented positive SARS-CoV-2 diagnostic testing with sample collected ≤7 days at Day 1 of pre-screening
2. No COVID-19 symptoms (defined as fever, chills, myalgia, arthralgia, headache, fatigue, cough, sore throat, nasal congestion, anosmia, ageusia, nausea, vomiting, or diarrhea, that are either new onset or have worsened from baseline*).

*Baseline refers to health status prior to contracting COVID-19.

**Informed** **Consent**

1. Capable of giving informed consent, which includes compliance with the requirements and restrictions listed in the Pre-screening informed consent form (ICF)

**Sex**

1. Male or female

**Others**

1. Willing to be contacted by study team and to notify study team if any COVID-19 symptom occurs
2. Interested in participating in a treatment trial if symptoms of COVID-19 occur.

### Pre-Screening Procedures

The pre-screening Schedule of Activities is shown in Table 4.

Table 4: Pre-screening Schedule of Activities

| **Pre-screening Visits^1^** | **Eligibility check**  **(≤72 hrs)** | **Pre-screening**  **(Day 1 to Pre-screening**  **Day 10)** |
| --- | --- | --- |
| Obtain informed consent | X |  |
| Eligibility criteria | X |  |
| SARS-CoV-2 diagnostic testing^1^ | X |  |
| Daily contact with study team to check if symptomatic for COVID-19^2^ |  | X |
| No AEs will be collected | | |

^1^If documented positive SARS-CoV-2 testing is not available at the eligibility check visit, testing may be performed provided that the potential participant has had a high risk exposure to SARS-CoV-2, defined as being an immediate household contact of a confirmed COVID-19 case and/or having occupational exposure to a confirmed COVID-19 case (for example, healthcare worker or first responder).

^2^Participant will exit the pre-screening phase if he/she remains asymptomatic at day 10 after diagnosis or day 10 of the pre-screening phase, whichever comes first.

After the pre-screening ICF is signed and eligibility is confirmed, participants are asked to notify the study staff immediately if any COVID-19 symptom occurs. Study staff will also contact participants daily by phone or text or electronically to inquire about symptoms. If a participant does not have any COVID-19 symptoms at day 10 from SARS-CoV-2 diagnosis or day 10 of the pre-screening follow-up, the participant will be asked to exit the pre-screening phase.

Note that no data during the pre-screening stage will be recorded in the database or in a case report form.

## Screening for the Study

Refer to Section 8.1 for screening procedures.

### Inclusion and Exclusion Criteria

Participants are eligible to be included in the study only if all of the following criteria apply:

**Age**

1. ≥18 years of age at the time of informed consent

**Type of Participant**

1. Participants must be at high-risk for COVID-19 progression by fulfilling at least **one** of the following criteria at screening:

- Age ≥65 years
- Presence of chronic pulmonary disease, chronic obstructive pulmonary disease (COPD), pulmonary hypertension
- Diabetes mellitus (type 1 or type 2), requiring oral medication or insulin for treatment
- Hypertension, requiring at least one oral medication for treatment
- Immunocompromised status due to disease (e.g., those living with human immunodeficiency virus with a CD4 T-cell count of <200/mm^3^)
- Immunocompromised status due to medication (e.g., taking 20 mg or more of prednisone equivalents a day, anti-inflammatory monoclonal antibody therapies, cancer therapies)
- Any chronic disease that is associated with high risk for severe COVID disease in the opinion of the site investigator
- Body mass index ≥35 kg/m^2^ (based on self-reported weight and height).

**COVID-19 Characteristics**

1. Documented positive SARS-CoV-2 diagnostic testing with a sample collected ≤10 days of screening.
2. Symptomatic for COVID-19 for ≤7 days at time of randomization

(symptomatic is defined as having at least one of the following symptoms of COVID-19 that is of new onset or has worsened from baseline: fever, chills, myalgia, arthralgia, headache, fatigue, cough, sore throat, nasal congestion, nausea, vomiting, or diarrhea).

**Informed** **Consent**

1. Capable of giving informed consent, which includes compliance with the requirements and restrictions listed in the ICF and in this protocol

**Sex**

1. Male or female

**Other Requirements:**

1. Agree to participate in all remote, in-person or home visits as required in the protocol and provide updated contact information as necessary.
2. Female of childbearing potential must agree to practice adequate contraception during the study (Section 9.8.1).

### Exclusion Criteria

Participants are excluded from the study if any of the following criteria apply:

**Medical Conditions or history**

1. Currently hospitalized or under immediate consideration for hospitalization at screening and Day 1
2. Have new onset shortness of breath or increased shortness of breath from pre-COVID-19 (for people with known COPD) at screening and Day 1
3. Hypoxemia (oxygen saturation<94% in ambient air or oxygen saturation below pre-COVID-19 oxygen saturation [if known] for people with known COPD) at Day 1
4. Require supplemental oxygen (new requirement or increase in requirement from pre-COVID-19 condition) at screening and Day 1
5. Have a history of (in the past 3 months) or current active pathological bleeding
6. Have a history of hemorrhagic stroke or intracranial hemorrhage
7. Have a recent severe head trauma within 30 days which includes concussion, skull fracture or hospitalization for head injury
8. Have known intracranial neoplasm, cerebral metastases, arteriovenous malformation or aneurysm
9. Have history of pregnancy-related hemorrhage
10. Have active gastroduodenal ulcer or other gastrointestinal bleeding diagnosed in the past 3 months
11. Currently are in a hemodynamically unstable state
12. Currently require thrombolysis or pulmonary embolectomy
13. Have history of severe hypersensitivity reaction to Xarelto®
14. Currently have a prosthetic heart valve
15. Have known diagnosis of triple positive antiphospholipid syndrome
16. Have known diagnosis of chronic kidney disease (stage IV or receiving dialysis)
17. Have a history of thrombocytopenia or known platelet count < 100,000 cells/mm^3^
18. Have a history of bronchiectasis and pulmonary cavitation
19. Have active cancer (eg, receiving chemotherapy or treatment for complication of the active cancer)

**Other:**

1. Had epidural or neuraxial anesthesia or spinal puncture in the past 2 weeks or plan to undergo these procedures during the study
2. Had surgery in the past 4 weeks or plan to undergo surgery during the study
3. Currently is pregnant or plans to become pregnant (refer to Section 9.8)
4. Currently is breastfeeding
5. Share household with an enrolled participant in this study
6. Co-enrollment in any clinical trial that includes prohibited procedures (spinal puncture or surgery) or that includes treatments within the same drug class as rivaroxaban or treatments for which co-administration with rivaroxaban are prohibited*

*Note that any co-enrollment other than this requires approval by the Sponsor. For any co-enrolled study, the total volume of blood samples collected across the studies should not exceed 275 mL in 4 weeks.

**Medications:**

1. Currently using and plan to use the following medications during the study

- Rivaroxaban or drugs in the same class
- Dual anti-platelets therapy
- Other anticoagulants (Table 5)
- Combined P-gp and CYP3A inhibitors and inducers (Table 5).

# Study Intervention- Rivaroxaban or Placebo

Participants will be randomly assigned to either the rivaroxaban group or placebo, in parallel to receive:

- Rivaroxaban group: 10mg (1 tablet) taken orally each day for 21 days.
- Placebo group: 1 tablet taken orally each day for 21 days.

The rivaroxaban (10mg) used in the active group will be Xarelto® 10mg tablet commercially marketed in the US.

For the placebo group, a placebo-equivalent will be used. This will be an over the counter dietary multi-vitamin supplement tablet, commercially marketed with their Product # 501-01 in the US by Geri-Care Pharmaceuticals Corp, based on its suitability to maintain masking to the extent possible.

The active and placebo tablets will not be identical in appearance; but will be similar in size, shape and color. Moreover, the bottles will be identical.

The bottle of medication the participants receive will not identify the treatment allocation. The active or placebo bottles will be labeled for investigational use with a randomized number to maintain blinding.

Refer to Section 6.8 for preparation, handling, storage and accountability of the study IPs.

## Rationale for Study Intervention Selection

Refer to Section 3.2.2 for the rationale for use of rivaroxaban for treatment of mild COVID-19 and to Section 3.2.3.1 for benefits and risks of rivaroxaban.

## Management of Participants to Limit Risks of SARS-CoV-2 Transmission

To limit the transmission of SARS-CoV-2, remote monitoring will be done as much as it is feasible via video conference, text message, telephone or other methods, as appropriate. In-person or home visits may be required for administration or monitoring.

For any in-person contact, local guidance for COVID-19-related safety measures will be followed.

## Guidance on COVID-19 to Ensure Participant and Healthcare Worker Safety

COVID-19 specific guidance from public health authorities in the country in which the study will be conducted should be followed.

As an example, the FDA Guidance for Industry on COVID-19 provides general considerations to assist the Sponsor in assuring the safety of trial participants, maintaining compliance with good clinical practice (GCP), and minimizing risks to trial integrity for the duration of the COVID-19 public health emergency [FDA 2020]. For example, the Sponsor should consider each circumstance, focusing on the potential impact on the safety of trial participants, and modify study conduct accordingly. Sponsors, in consultation with clinical principal investigators (PIs) and Institutional Review Boards (IRBs)/Independent Ethics Committees (IECs), may determine that the protection of a participant’s safety, welfare, and rights is best served by continuing a study participant in the trial as per the protocol or by discontinuing the administration or use of the investigational product or even participation in the trial. Such decisions will depend on specific circumstances, including the nature of the investigational product, the ability to conduct appropriate safety monitoring, etc.

Changes in study visit schedules, missed visits, or patient discontinuations may lead to missing information (e.g., for protocol-specified procedures). It will be important to capture specific information in the case report form that explains the basis of the missing data, including the relationship to COVID-19 for missing protocol-specified information (e.g., from missed study visits or study discontinuations due to COVID-19).

For instances where efficacy endpoints are not collected, the reasons for failing to obtain the efficacy assessment should be documented (e.g., identifying the specific limitation imposed by COVID-19 leading to the inability to perform the protocol-specified assessment).

## Dose Modification and Toxicity Management

There will be no dose modification.

Participants who experience major bleeding or clinically relevant non-major bleeding will have the study intervention discontinued (see Section 7.1) and managed according to standard of care.

Participants who experience other AEs due to the study medication may discontinue the study medication per the clinical judgment of the investigator.

At each visit, the participant will be asked if any bleeding events have occurred. Major bleeding events will be recorded as AESIs and non-major bleeding events will be recorded as AEs, in the eCRF. The following definitions will apply.

### Definitions of Bleeding Severity

The following definitions are based on the guidance for non-surgical patients by the International Society on Thrombosis and Hemostasis (ISTH)/Scientific and Standardization Committee (SCC).

#### Definition of Major Bleeding

- Fatal bleeding AND/OR
- Symptomatic bleeding in a critical area or organ, such as intracranial, intraspinal, intraocular, retroperitoneal, intra-articular or pericardial, or intramuscular with compartment syndrome. AND/OR
- Bleeding causing a fall in hemoglobin level of 2 g/dL (1.24 mmol/L) or more or leading to transfusion of two or more units of whole blood or red cells.

#### Definition of Clinically Relevant Non-Major Bleeding

A clinically relevant non-major bleed is an acute or subacute clinically overt bleed that does not meet the criteria for a major bleed but prompts a clinical response, in that it leads to at least one of the following:

- A hospital admission for bleeding, OR
- A physician guided medical or surgical treatment for bleeding, OR
- A change in antithrombotic therapy (including interruption or discontinuation of study drug).

In addition, bleeding events that lead to participant’s discomfort, and impairment of activities of daily life are considered clinically relevant non-major bleeding.

Examples of clinically relevant non-major bleeding are:

- Epistaxis if it lasts for more than 5 minutes, if it is repetitive (i.e., 2 or more episodes of true bleeding, i.e., not just spots on a tissue, within 24 hours), or leads to an intervention (packing, electrocoagulation, etc)
- Gingival bleeding if it occurs spontaneously (i.e., unrelated to tooth brushing or eating), or if it lasts for more than 5 minutes
- Hematuria if it is macroscopic, and either spontaneous or lasts for more than 24 hours after instrumentation (e.g., catheter placement or surgery) of the urogenital tract
- Macroscopic gastrointestinal hemorrhage: at least 1 episode of melena/hematemesis, if clinically apparent
- Rectal blood loss, if more than a few spots
- Hemoptysis, if more than a few speckles in the sputum
- Intramuscular hematoma
- Large subcutaneous hematoma (more than 25 cm^2^)
- Multiple source bleeding.

#### Definition of Minimal Bleeding

Other bleeding events that do not meet the criteria for either major bleeding or clinically relevant non-major bleeding are considered minimal bleeding.

## Safety Monitoring

Safety monitoring will take place once the informed consent has been signed. On Day 1, prior to IP, the participant will be asked about his or her health and about any bleeding issues prior to taking the first dose. The participant will then be in frequent contact with the study staff, with the final visit being 14 days after the last medication dose.

The participant will have a contact number to call the staff at any time should he or she have a non-emergency study related health issue that they need to discuss in between visits. As previously mentioned, if needed, additional contact with the study clinician or staff will be conducted at the request of the participant (e.g., if developing concerning symptoms or an AE). Refer to Section 8 for more information.

If it is a health emergency, they will be asked to call the country nationwide health emergency number (like 911 in the USA). Refer to Section 8 for more information regarding study procedures for safety.

## Study Pausing Guidelines

Pausing guidelines are put in place to address medical events necessitating a pause in enrollment and in participant dosing, and trigger IDMC reviews. Therefore, these guidelines are in effect during the active enrollment and dosing period. Any of the below conditions, if identified either by the study staff, investigator, the Sponsor or the IDMC, will trigger a pause of the enrollment and pause of administration of study intervention until the IDMC has reviewed the safety data and made a recommendation on how to proceed:

1. One report of anaphylaxis with or without bronchospasm within 4 hours of taking study intervention indicative of an immediate hypersensitivity reaction to the study intervention
2. One report of major bleeding judged as related to study intervention by the investigator.

## Masking

Every effort will be made to mask the assigned treatment groups. The participants will not be told of the assigned treatment groups and will not be informed that active and control treatment do not look identical. However, the participant could possibly find out this information because of features like embossment on the rivaroxaban tablets, which are not present on the placebo-equivalent tablets.

The study staff evaluating the participants will not be given the assigned treatment groups but could possibly find out if the participant’s tablets become visible to them. Such occurrences will be documented. It is recognized that they could potentially bias the outcome assessments by the participant and study staff particularly for subjective measurements.

In order to preserve masking, household members of an enrolled participant will be excluded from participating in this study.

## Preparation/Handling/Storage/Accountability

Each participant will receive a bottle containing twenty-two (22) tablets of active or placebo based on the randomization. Twenty-one (21) tablets are meant for each participant’s 21 days of daily dosing. One extra tablet is provided as overage.

Xarelto®, per US Commercial Package Insert, is to be stored at 25°C (77°F) or room temperature with excursions permitted to 15° to 30°C (59° to 86°F). The same conditions will apply to the placebo-equivalent. The temperature conditions will be printed on the investigational label applied on the bottle.

Documentation of receipt, dosing, and disposal of unused medication to be maintained as per study procedures.

Study medication may be dispensed by a qualified study representative, according to dispensing instructions. Study medication may also be shipped directly to the participant’s home depending on evolving circumstances related to COVID-19. Instructions will be included with the drug shipment to the participants.

Date of IP dispensing and adherence to IP administration will be recorded.

## Study Intervention Compliance

The IP will be self-administered at home, and the participants will be contacted to ensure that they have received and appropriately stored the IP. Study participants will be asked about adherence to the IP.

## Concomitant Therapy

The prohibited medications in this study are other anticoagulants, and combined P-gp and CYP3A inhibitors and inducers because of their significant PK and PD interactions with rivaroxaban. Should administration of any prohibited drug (Table 5) be indicated for participants’ clinical care, the study medication must be discontinued. Other drugs may have PK and PD interactions with rivaroxaban and thus are considered precautionary medications.

Refer to Section 6.10.1 for prohibited medications and to Section 6.10.2 for precautionary medications.

Whether the study medication should be discontinued or allowed to be used with these precautionary medications will be under the clinical judgement of the treating physician (i.e., either the PI, or the physician responsible for the patient’s care if the participant is hospitalized).

***Pharmacodynamic Interactions***

The concurrent use of rivaroxaban with **other anticoagulants, antiplatelet agents, and nonsteroidal anti-inflammatory** agents is expected to increase the risk of bleeding in comparison to use of rivaroxaban alone (refer to Table 5).

***Pharmacokinetic Interactions***

The absorption of rivaroxaban is mediated by P-gp. P-gp inhibitors can increase the absorption of rivaroxaban, increasing both AUC and Cmax. Conversely, P-gp inducers can reduce the absorption of rivaroxaban, decreasing AUC and Cmax.

The metabolism of rivaroxaban is mediated by CYP3A4. CYP3A4 inhibitors can decrease the metabolism of rivaroxaban, increasing both AUC and Cmax. Conversely, CYP3A4 inducers can increase the metabolism of rivaroxaban, decreasing AUC and Cmax.

Agents that interfere with both P-gp and CYP3A4 are likely to cause more significant interactions with rivaroxaban than agents that interfere with P-gp or CYP3A4 alone.

### Prohibited Medications

Table 5: Rivaroxaban prohibited medications/foods/supplements

| **Drug Class** | **Examples (based on human *in vivo* data^1^)** | **Known or Probable Effect** |
| --- | --- | --- |
| Anticoagulants | warfarin sodium (Coumadin®, Jantoven®), any medicine that contains heparin, including low molecular weight heparins, apixaban, Pradaxa [dabigatran], Savaysa [edoxaban], Bevyxxa [betrixaban], non-study Xarelto [rivaroxaban] | Increased risk of bleeding |
| Combined P-gp inhibitors and *strong* CYP3A4 inhibitors | cobicistat, conivaptan, indinavir,  itraconazole, ketoconazole, posaconazole, ritonavir, saquinavir, telaprevir, telithromycin | Significant increase in rivaroxaban concentration |
| Combined P-gp inhibitors and moderate CYP3A4 inhibitors OR strong CYP3A4 inhibitors alone | amiodarone, azithromycin, clarithromycin, cyclosporine, diltiazem, dronedarone, erythromycin, fluconazole, grapefruit, lapatinib, mifepristone, nefazodone, nicardipine, ranolazine, tamoxifen, ticagrelor, verapamil, voriconazole | Moderate increase in rivaroxaban concentration in patients with normal renal function.  Significant increase in rivaroxaban concentrations in patients with renal impairment |
| Combined P-gp inducers and *strong* CYP3A4 inducers | carbamazepine, dexamethasone, rifampin, St John's wort | Significant reduction in rivaroxaban concentration  Effect may persist for several weeks following discontinuation of strong inducers of P-gp and/or CYP3A4 |
| Inducers of P-gp | tipranavir | Significant reduction in rivaroxaban concentration  Effect may persist for several weeks following discontinuation of strong inducers of P-gp and/or CYP3A4 |
| *Strong* inducers of CYP3A4 | bosentan, efavirenz, etravirine, fosphenytoin, nafcillin, nevirapine, phenobarbital, phenytoin, primidone, rifabutin, rifapentine | Significant reduction in rivaroxaban concentration  Effect may persist for several weeks following discontinuation of strong inducers of P-gp and/or CYP3A4 |

Modified from [UW 2020]

### Precautionary Medications

Participants are discouraged from using the following medications that may increase the risk of bleeding during the study period.

- aspirin or aspirin-containing products
- long-term (chronic) use of non-steroidal anti-inflammatory drugs (NSAIDs)
- clopidogrel (Plavix®)
- selective serotonin reuptake inhibitors (SSRIs) or serotonin norepinephrine reuptake inhibitors (SNRIs)
- other medicines to prevent or treat blood clots.

## Hospitalization

If a participant clinically worsens, such as requiring hospitalization, it is expected that standard clinical care will be provided. Hospitalized participants may continue to receive study intervention if maintained on the originally randomized study-intervention regimen.

Accommodations will be made to facilitate these encounters within this specific setting. Clinical outcomes in hospitalized participants will be confirmed through the electronic health record, if possible.

## Treatment after the End of the Study

No additional treatment or study intervention will be provided at the end of the study.

# Discontinuation/Withdrawal Criteria and Study Completion

## Discontinuation of Study Intervention

Study intervention will be discontinued for the following reasons:

- Hospitalization, if the in-patient provider decides continuation of study treatment is contraindicated.
- Requirement for prohibited concomitant medications or other contraindication to study product
- Occurrence of an AE requiring discontinuation of study intervention (i.e., severe hypersensitivity, major bleeding or clinically relevant non-major bleeding)
- Request by participant to terminate study intervention
- Clinical reasons believed to be life-threatening by the physician, even if not addressed in this protocol
- Pregnancy.

Participants who stop study intervention should continue study participation while off of the study intervention, with continued evaluations as per the SoA. The reason for study intervention discontinuation should be recorded.

## Withdrawal from the Study

A participant may withdraw from the study at any time at the participant’s request or may be withdrawn at any time for the following reasons:

- At the request of the primary care provider if being in the study is no longer in the best interest of the participant
- Participant is judged by the PI to be at significant risk of failing to comply with the provisions of the protocol as to cause harm to self or seriously interfere with the validity of the study results
- At the discretion of the IRB/IEC or government agencies as part of their duties, PI, or Sponsor.
- The participant progressed to moderate or severe COVID-19 (or worse) prior to initiation of IP dosing on Day 1

If the participant withdraws consent for disclosure of future information, the Sponsor may retain and continue to use any data collected before such withdrawal of consent.

Upon withdrawal from the study, the participant may request destruction of any biological samples taken and not tested, and the PI or designee must document this in the site study records.

## Lost to Follow-up

A participant will be considered lost to follow-up if the participant is unable to be contacted by the study site.

The following actions must be taken if a participant fails to comply with required study procedures:

- The site must attempt to contact the participant as soon as possible and counsel the participant on the importance of maintaining the assigned procedure schedule and ascertain whether or not the participant wishes to and/or should continue in the study.
- Before a participant is deemed lost to follow-up, the PI or designee must make every effort to regain contact with the participant. These contact attempts should be documented in the participant’s medical record.
- Should the participant continue to be unreachable, the participant will be considered to have withdrawn from the study with a primary reason of lost to follow-up.

## Study Completion

A participant is considered to have completed the study after completion of the last scheduled procedure shown in the SoA.

This study will be considered completed when all participants have completed the study.

Pregnant and lactating females can remain in the study but must discontinue the study medication (refer to Section 9.8).

# Study Encounters

Study encounters will be done remotely, via web-based video conference, text message, telephone or other methods, as appropriate to limit the transmission of SARS-CoV-2. In-person and home visits are not anticipated, but may be needed on an ad hoc basis for some participants when remote monitoring is inadequate to fulfill the requirement of the study, or if deemed necessary for the safety of the participant based on the clinical judgement of the study staff.

Maintenance of participant’s privacy is important for all types of study encounters. For each encounter, the study investigators or designees and the trial participants should confirm their respective identities with one another before engaging in the visit.

Participants will be instructed to seek clinical care should they manifest any signs or symptoms of COVID-19 progression requiring medical intervention and notify the treating physician about trial participation.

## Screening Process and Procedures

The following procedures must occur ≤5 days prior to the Day 1 visit. All screening evaluations must be completed and reviewed to confirm that potential participants meet all eligibility criteria.

The PI/study staff will maintain a screening log to record details of all participants screened and to confirm eligibility or record reasons for screening failure, as applicable. This does not apply to any pre-screening procedures.

Procedures conducted as part of the participant’s routine clinical management and obtained before signing the informed consent form may be utilized for screening purposes provided that the procedures met the protocol-specified criteria and were performed within the time frame defined in the SoA.

The study staff conducting the Screening visit will:

- Introduce themselves and their role to the participant, and confirm the identity of the participant.
- Confirm that the means of contact is adequate to conduct the contact/visit
- Obtain informed consent (refer to Section 11.1.2 for details)
- Collect demographic information -age, sex, race and ethnicity and personal information including name and address
- Collect information in past and current medical conditions, including known pregnancy and/or lactation status
- Collect concomitant medication information (refer to Section 6.10 for details).
- Collect documented positive SARS-CoV-2 diagnostic testing performed with a sample collected ≤10 days of screening. If no document is available, SARS-CoV-2 diagnostic testing will be performed at screening.
- Check inclusion and exclusion criteria.

Eligible participants who meet all criteria will be enrolled and randomized to a study group and will receive supplies and study IP, delivered to the participant at home, or if deemed necessary, it may be picked up at study site or other methods may be used as appropriate.

Eligible participants will receive the following:

- Study IP with dosing direction and storage information
- An oximeter and a thermometer, with user instructions
- Nasal test kit, with user instructions
- Personal protective equipment (face masks, shields and gloves).

# Study Assessments and Procedures

Refer to the Section 2, Table 1, for the Schedule of Activities (SoA). Note that unscheduled visits may be required. Additional collection of a swab sample may be performed based on the clinical judgement of the PI or designee.

Immediate safety concerns should be addressed by the PI as soon as he or she becomes aware of the concern to determine if the participant should continue or discontinue study IP. The PI will inform the Sponsor immediately upon occurrence or awareness.

Adherence to the study design requirements, including those specified in the SoA, is essential and required for study conduct.

## Day 1 (first day of study)

The study staff conducting the visit will:

- Introduce themselves and their role to the participant and confirm the identity of the participant
- Confirm that the means of contact is adequate to conduct the contact/visit
- Confirm that the supplies and the study IP were received in good condition, review the contents received and instructions.
- Remind the participant not to show the study IP tablets if it is a video enabled contact or describe the tablets detailed appearance to maintain the masking of the study staff.
- Collect information on COVID-19 symptoms and signs
- Assess the Gates MRI and WHO ordinal scales and confirm that participant continues to meet criteria for Scale 2 of Gates MRI COVID-19 Ordinal Scale (Table 7)
- Collect information on AEs (note that AEs will be collected from the time the participant signs the informed consent).
- Collect information on concomitant medications
- Collect information on adherence to study IP and any dosing error (if applicable)
- Ask participant to collect nasal sample, temperature, and oxygen saturation during the encounter (study staff will document temperature and oxygen saturation).
- Inquire about pregnancy, as appropriate.

## Procedures at Visits from Day 4 through Day 28

At each of these visits on Days 4, 6, 8, 10, 12, 14, 18, 21, 24, 28, the study staff conducting the visit will:

- Introduce themselves, explain their role to the participant, and confirm the identity of the participant
- Confirm that the means of contact is adequate to conduct the contact/visit
- Collect information on COVID-19 symptoms and signs
- Assess the Gates MRI and WHO ordinal scales
- Collect information on AEs
- Collect information on bleeding events and severity
- Collect information on concomitant medications
- Remind the participant to take the dose daily through Day 21, and not to show the study IP tablets or describe the tablets detailed appearance, to maintain the masking of the study staff.
- Collect information on adherence to study IP and any dosing error (if applicable)
- Ask participant to collect temperature, and oxygen saturation during the encounter (study staff will document temperature and oxygen saturation)
- Ask participant to collect nasal sample during the encounter for Days 4, 8, 14, 21 and 28
- Inquire about pregnancy, as appropriate.

As needed, additional contact with the study clinician or staff will be conducted at the request of the participant (e.g., if developing concerning symptoms or an AE).

## Procedures at Visit Day 35

This will be the last visit. The study staff conducting the contact/visit will:

- Introduce themselves and their role to the participant and confirm the identity of the participant
- Confirm that the means of contact is adequate to conduct the contact/visit
- Collect information on AEs
- Collect information on bleeding events and severity
- Collect information on concomitant medications
- Inquire about pregnancy, as appropriate.

## Efficacy Assessments

The proportion of participants who progress to moderate or severe category or higher (Gates MRI ordinal scale ≥3) will be assessed at study visits indicated in the SoA.

### Nasal Sampling

Nasal sampling for SARS-CoV-2 diagnostic testing will follow study instruction document from the testing laboratory. Nasal sampling collection will be discontinued during hospitalization and/or, per physician’s discretion, when two consecutive negative results are available at any time during the study.

Instructions on handling, storage and shipment or delivery of nasal swabs that are collected by the participant will be provided to the participant and explained during the visit.

Additional nasal swab collection may be performed based on the clinical judgement of the PI or designee.

## Safety Assessments

Safety will be assessed at study encounters by the study investigator or designee. Note that unscheduled visits may be required.

Participants will be interviewed by study staff for AEs.

AEs will be collected from the time informed consent is obtained through Day 35 (14 days after the last dose of IP).

SAEs and AESIs (major bleeding and severe hypersensitivity to rivaroxaban) will be reported to the Sponsor or designee immediately (within less than 24 hours), as also any follow-up information.

All AEs must be recorded on the eCRFs if any of the following criteria have been met:

- AEs meeting SAE definition
- AEs leading to discontinuation of study intervention
- Grade 3 and grade 4 AEs
- Adverse events of special interest (AESI) including major bleeding (refer to Section 6.4.1) and severe hypersensitivity to rivaroxaban.

AEs will be assessed at the time of the collection by the investigator as related or not related to the study.

The Sponsor will be responsible for the expectedness assessment according to the available reference safety information and for complying to applicable expedited and periodic AE reporting obligations to regulatory authorities, investigators and other entities.

All AEs will be followed until resolution, stabilization, lost to follow-up, or until the end of the study (Day 35).

### Adverse Event Definition

An AE is any untoward medical occurrence that occurs in a participant administered an IP. An AE can be any unfavorable and unintended sign (including abnormal laboratory findings), symptom, or disease temporally associated with the use of an IP, whether or not considered related to the product.

AEs may include the onset of new illness and the exacerbation of pre-existing medical conditions. An AE can include an undesirable medical condition occurring at any time, including baseline, even if no study intervention has been administered.

### Adverse Events of Special Interest Definition

An adverse event of special interest (serious or non-serious) is one of scientific and medical concern specific to the Sponsor’s product or program, for which ongoing monitoring and rapid communication by the investigator to the Sponsor can be appropriate.

### Serious Adverse Event Definition

An SAE is defined as any untoward medical occurrence that at any dose:

- Results in death
- Is life-threatening
- Requires in-patient hospitalization or prolongation of existing hospitalization
- Results in persistent or significant disability/incapacity
- Is an important medical event that may not be immediately life-threatening or result in death or hospitalization but may jeopardize the participant or may require intervention to prevent one of the other outcomes listed in the definition above.

### Toxicity Grading

All AEs that are recorded must include an intensity grade. To grade AEs, sites should refer to the specific toxicity grading by Division of Acquired Immunodeficiency Syndrome (DAIDS) AE Grading Table [DAIDS 2017].

### Study Intervention-Related AEs and SAEs

A study intervention-related AE is defined as any new AE that begins, or any preexisting condition that worsens in severity, after at least 1 dose of study intervention has been administered and is considered by the PI to be related to the study intervention.

All AEs and SAEs should have attribution recorded as study IP-related or not study IP-related, in the judgment of the site investigator.

All SAEs, regardless of whether related or not to the IP, will be followed until resolution, stabilization, lost to follow-up, or until the end of the study (Day 35).

## Assessment of COVID-19 Signs and Symptoms

COVID-19 signs and symptoms reported to the study staff to be recorded in the CRF/eCRF are shown in Table 6**.** Other symptoms or signs considered by the PI to be related to COVID-19 can be added as necessary. Of note, anosmia and ageusia will be collected but not included in the symptom endpoint analysis as they may be long-lasting despite resolution of other symptoms (possibly due to direct viral damage of the olfactory and gustatory receptors). Therefore, their continued presence post therapy may not be indicative of a poor response to study intervention(s).

The PI or designee will query the participants about the symptoms listed below and determine symptom resolution (yes or no) since their last visit.

Table 6: COVID-19 Signs and Symptoms to be Recorded in the CRF/eCRF

| Temperature (fever >100.4°F or >38°C) |
| --- |
| Oxygen saturation |
| Chills |
| Muscle pain |
| Joint pain |
| Headache |
| Fatigue |
| Cough |
| Sore throat |
| Nose congestion |
| Nausea |
| Vomiting |
| Diarrhea |
| Shortness of breath |
| Chest pain |
| Loss of sense of smell (anosmia) |
| Loss of sense of taste (ageusia) |
| Other symptoms and signs may be added |

The Gates MRI COVID-19 Ordinal Scale (Table 7) and the WHO Ordinal Scale for Clinical Status (Table 8) will be completed by the PI or a medically qualified designee.

Table 7: Gates MRI COVID-19 Ordinal Scale Clinical Endpoint Definitions

| **Scale** | **Category** | **Endpoint definition** |
| --- | --- | --- |
| 1 | Asymptomatic/symptoms similar to pre-COVID status | - No symptoms and signs AND - No limitation of daily activities |
| 2 | Mild | - Symptomatic AND - No shortness of breath AND - No hypoxemia (O_2_ saturation ≥94% in ambient air) |
| 3 | Moderate or severe | - Symptomatic AND - Shortness of breath* OR tachypnea (respiratory rate ≥ 20 min)* OR hypoxemia (<94% in ambient air)* |
| 4 | Critically ill | - Symptomatic AND - Receiving high flow oxygen OR non-invasive mechanical ventilation |
| 5 | Critically ill with invasive mechanical ventilation or extrapulmonary complication | - Symptomatic AND - Receiving invasive mechanical ventilation OR Life threatening or debilitating extrapulmonary complications |
| 6 | Critically ill with Extra-Corporeal Membrane Oxygenation (ECMO) | - Symptomatic AND - Receiving ECMO |
| 7 | Death | - Death |

*For known COPD participants, moderate or severe category requires worsening of shortness of breath or respiratory rate or oxygen saturation from pre-COVID-19 status

Table 8: WHO Ordinal Scale for Assessment of Clinical Status of COVID-19 Patients

| **Participant Status** | **Descriptor** | **Score** |
| --- | --- | --- |
| Uninfected | Uninfected; no viral RNA detected | 0 |
| Ambulatory mild disease | Asymptomatic; viral RNA detected | 1 |
|  | Symptomatic; independent | 2 |
|  | Symptomatic; assistance needed | 3 |
| Hospitalized moderate disease | Hospitalized; no oxygen therapy* | 4 |
|  | Hospitalized; oxygen by mask or nasal prongs | 5 |
| Hospitalized severe disease | Hospitalized; oxygen by NIV or high flow | 6 |
|  | Intubation and mechanical ventilation, pO2/FiO2 ≥150 or SpO2/FiO2 ≥200 | 7 |
|  | Mechanical ventilation pO2/FiO2 <150 (SpO2/FiO2 <200) or vasopressors | 8 |
|  | Mechanical ventilation pO2/FiO2 <150 and vasopressors, dialysis, or ECMO | 9 |
| Dead | Dead | 10 |

WHO clinical progression scale, WHO Working group [WHO(c**)** 2020]

ECMO=extracorporeal membrane oxygenation. FiO2=fraction of inspired oxygen.

NIV=non-invasive ventilation. pO2=partial pressure of oxygen. SpO2=oxygen saturation.

*****If hospitalized for isolation only, record status as for ambulatory patient

## Overdose of Study Intervention

Study medication overdose (rivaroxaban and placebo) including misuse or abuse of the product and medication errors, should be reported to the study investigator immediately and reported in the clinician notes and the eCRF.

An approved reversal agent for rivaroxaban is andexanet alfa. Its use will be based on the clinical judgement of the treating physician.

## Pregnancy

The investigator or designee will be required to inquire about pregnancy at screening and every study visit but pregnancy testing will not be required.

Pregnancies occurring in participants enrolled in this study must be reported, and details collected including outcome of the pregnancy.

If pregnancy is reported, the participant should be withdrawn from study treatment but continue to be followed for safety. Details of the pregnancy as well as outcome should be followed and recorded.

Pregnancy alone is not regarded as an AE unless there is a possibility that the study IP may have interfered with the effectiveness of a contraceptive medication. Elective abortions without complications should not be considered AEs unless they were therapeutic abortions. Hospitalization for normal delivery of a healthy newborn should not be considered an SAE. Pregnancy is not considered an SAE unless there is an associated abnormal pregnancy outcome (e.g. spontaneous abortion, fetal death, stillbirth, congenital anomalies, ectopic pregnancy).

Any abnormal pregnancy outcomes during the study (e.g. spontaneous abortion, fetal death, stillbirth, congenital anomalies, ectopic pregnancy) should be reported as an SAE.

### Contraceptive Guidance

Women with history of hysterectomy, bilateral salpingectomy or bilateral oophorectomy are not considered females of childbearing potential.

Women physically capable of pregnancy must agree to use adequate contraception during the study. Examples include:

- abstinence from penile-vaginal intercourse, when this is their preferred and usual lifestyle
- oral contraceptives, either combined or progestogen alone
- injectable progestogen
- implants of etonogestrel or levonorgestrel
- estrogenic vaginal ring
- percutaneous contraceptive patches
- intrauterine device or intrauterine system
- male partner sterilization prior to the female participant’s entry into the study, and this male is the sole partner for that participant
- male condom combined with a vaginal spermicide (foam, gel, film, cream or suppository),
- male condom combined with a female diaphragm, either with or without a vaginal spermicide (foam, gel, film, cream, or suppository).

Adequate contraception does not apply to participants of child-bearing potential with same sex partners, when this is their preferred and usual lifestyle.

## Biohazard Containment

All dangerous goods and materials, including diagnostic specimens and infectious substances, will be handled and transported based on requirements of relevant local regulatory authorities.

# Statistical Considerations

## Sample Size Determination

Efficacy

The total number of participants planned to be enrolled is approximately 600 (300 per group). If the true control rate for progression to moderate or severe category or greater is at least 30%, 294 participants per group will result in 80% power when the true Study Intervention effect is 35%. This sample size assumes a drop-out rate of 10% and a 1-sided Type I error rate of 2.5%.

Safety

With 300 participants in the rivaroxaban intervention group, there is 80% probability to observe at least one SAE related to rivaroxaban when the true SAE rate is at least 0.54%.

## Statistical Hypothesis

Primary Hypothesis: Treatment with the DOAC rivaroxaban will reduce progression to moderate or severe disease category or higher (Gates MRI ordinal scale ≥3) relative to placebo in high risk participants with mild COVID-19.

## Populations for Analyses

For the purposes of analysis, populations are defined in Table 9.

Table 9: Populations for Analysis

| Population | Description |
| --- | --- |
| Intention to treat (ITT) population | All participants randomly assigned to study intervention. Participants will be analyzed according to the intervention to which they were randomized. |
| Modified intention to treat (mITT) populations | All participants randomly assigned to study intervention, who received the study intervention and have mild disease at study entry. Participants will be analyzed according to the intervention they actually received. |
| Per Protocol (PP) population | All participants randomly assigned to study intervention, who received the study interventions as planned, have mild disease at study entry, and did not substantially deviate from the protocol procedures.  Participants will be analyzed according to the intervention they actually received. |
| Safety population | All participants randomly assigned to study intervention, who received the study intervention. Participants will be analyzed according to the intervention they actually received. |

### Assigning Shared Controls across Multiple Intervention-Specific Analysis Populations

Each intervention may be initiated and stopped at different time frames, and within different sites. Depending on the number of interventions that a participant can be eligible to receive, a participant randomized to a control arm may be part of multiple intervention-specific analysis populations for the purposes of comparing a single intervention to control. We note the following core principles associated with sharing controls across intervention-specific analysis populations:

- Controls will only be shared across multiple intervention-specific populations when there is randomization across those interventions and the control. All participants will only be randomized once.
- A participant who is eligible for multiple interventions at a site will be randomized equally to receive one of the interventions for which they are eligible vs control. Participants randomized to control will be equally likely to receive the matched control of each intervention for which they are eligible. If the participant is randomized to control, they will be assigned to each of the intervention-specific analysis populations included in the randomization. A participant randomized to control will not be assigned to an intervention-specific analysis population that was not included in the randomization.
- Controls will only be shared contemporaneously. A participant can only be used in the intervention-specific population(s) that were available and included at the time of their randomization.
- A control does not have to receive the intervention-specific matched control to be eligible for an intervention-specific population. For example, a participant randomized 1:1:1 to receive two available interventions vs control would serve as a control for each of the interventions even if randomized to the matched control of the first interventions. The primary comparison will utilize eligible participants in the control arm with all control formulations.

The randomization strategy for up to 3 contemporaneously available interventions is shown in Figure 3. Strategies for 2 interventions and for 1 intervention are shown in Figure 4 and Figure 5, respectively.

This randomization strategy can easily be generalized beyond 3 contemporaneously available interventions and will ensure approximately equal randomization of each intervention to control, while helping to maintain blinding and minimize possible bias.

Participants randomized to rivaroxaban or control under the principles listed above will be assigned to the rivaroxaban-specific analysis population.

Figure 3: Randomization strategy for sites where 3 interventions are being studied


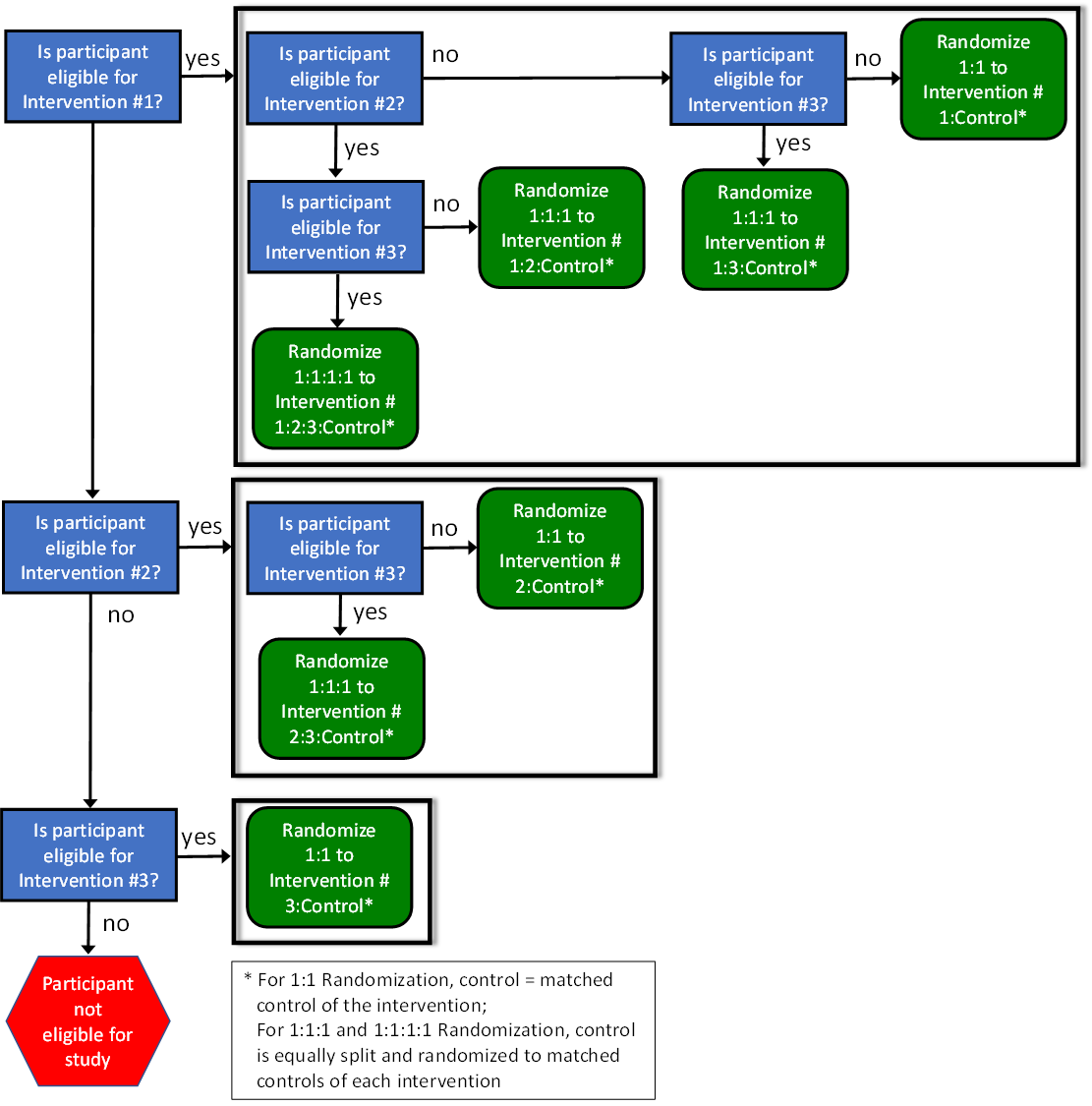


Figure 4: Randomization strategy for sites where 2 interventions are being studied


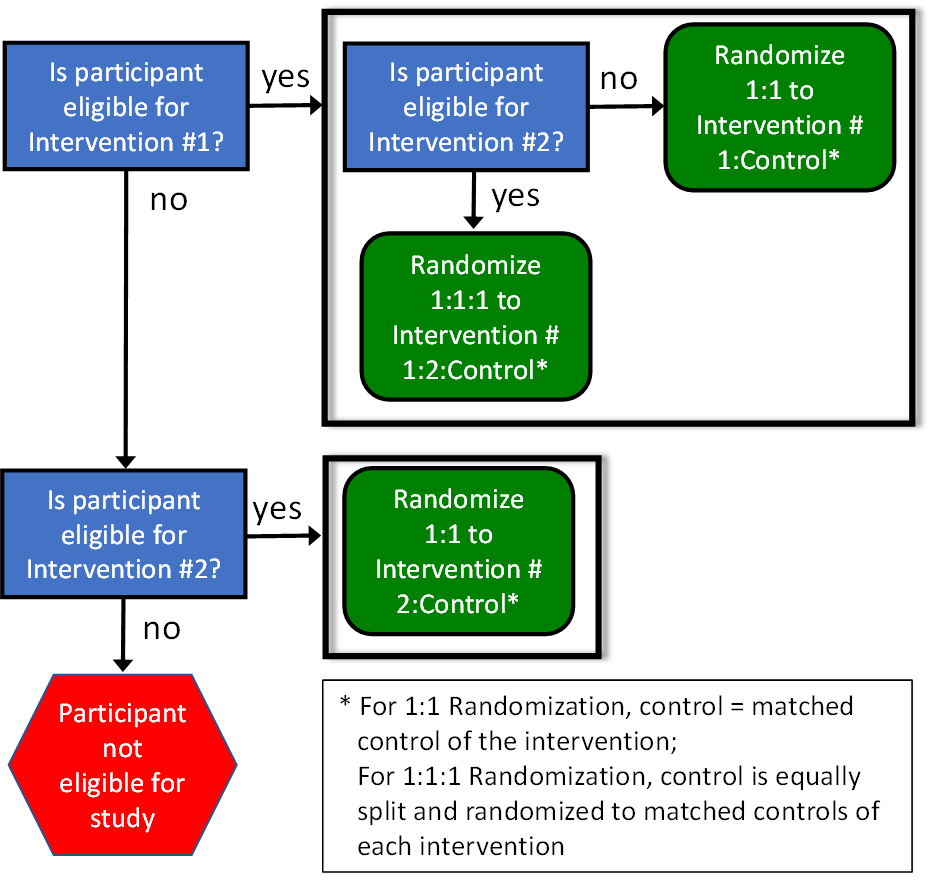


Figure 5: Randomization strategy for sites where only 1 intervention is being studied


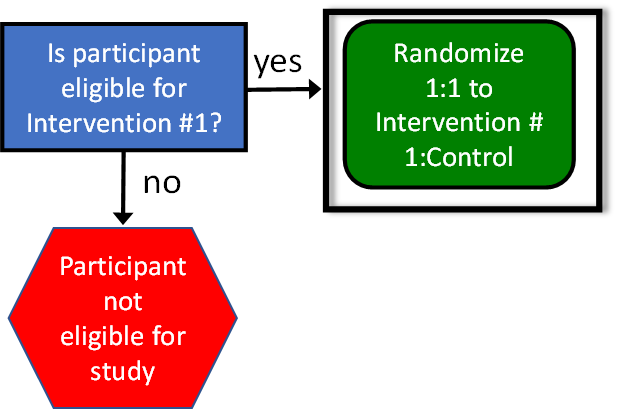


## Primary and Key Secondary Endpoints

The primary efficacy endpoint is the proportion of participants who progress to moderate or severe disease category or higher (Gates MRI ordinal scale ≥3) through Day 28.

Secondary efficacy endpoints include time to disease resolution, defined as symptoms resolution with or without viral clearance, proportion of participants with disease resolution, Gates MRI and WHO ordinal scales, and incidence and duration of hospitalization.

Primary safety endpoints include the frequency of Grade 3 and Grade 4 AEs, AEs resulting in study intervention discontinuation and SAEs.

## Statistical Methods

The statistical analysis plan will be developed and finalized before database lock and will describe the detailed analytical plans and procedures for the interim and final analyses, and accounting for missing data.

### Efficacy Analyses

**Primary efficacy endpoints**

The primary efficacy analysis will be conducted in the ITT population. Within the rivaroxaban analysis population as defined in Section 10.3.1, participants randomized to rivaroxaban will be compared to participants randomized to placebo/standard of care.

The proportion of participants who progress to moderate or severe disease category or greater through Day 28 will be summarized by treatment group. The absolute difference in the risk of progression to moderate or severe disease category or greater will be assessed using the stratified Miettinen and Nurminen method to compare the difference between two rates [Miettinen 1985].

Sensitivity analyses will be repeated as described above using the mITT and PP populations, to assess the robustness of the primary results.

**Secondary endpoints**

Secondary analyses will primarily be conducted in the ITT population, with sensitivity analyses performed in the mITT and PP populations. Specific details of each analysis will be provided in the SAP.

For time to disease resolution endpoints, a stratified Log-rank test will be used. A stratified Cox PH model will be used to estimate the hazard ratios and corresponding CIs associated with the rate of disease resolution for the study intervention relative to control. Due to anticipated heterogeneity in the rate of disease resolution, pre-specified baseline variables, including age, BMI, and days of symptoms at time of enrollment may be included in the model to increase precision. The appropriateness of the proportional hazard ratio assumption will be assessed.

The proportion of participants who achieve disease resolution at each time point through Day 28 will be summarized by treatment group with 95% exact CIs. Differences in the proportion of participants achieving disease resolution between each intervention and control will be assessed using the stratified Miettinen and Nurminen method to compare the difference between two rates [Miettinen 1985].

The distributions of the Gates MRI scale score and WHO ordinal score will be summarized by day and treatment group. The overall burden of disease for an individual participant may be calculated as the area under the curve over time. Differences in disease scores will be assessed using a linear model assessing the impact of treatment on disease burden, while controlling for baseline disease burden, age, BMI, and days of symptoms at time of enrollment. Differences in disease severity at key timepoints (e.g., Day 14) will also be assessed using a proportional odds model.

The proportion of hospitalized participants will be summarized by treatment group with 95% exact CIs. The relative risk of hospitalization for patients randomized to each intervention relative to control will be assessed using a stratified analysis for the comparison of two rates.

**Exploratory endpoints**

SARS-CoV-2 viral load measures will be assessed using a linear mixed effects model, with fixed effects of time, baseline viral load, age, BMI, and days of symptoms at time of enrollment, and a random effect for participant. The measures may be transformed to satisfy the normality assumption needed for the linear mixed effects model fit. Mean viral concentrations will be summarized by treatment group and compared over time based on the model fit.

Viral sequencing may be used to assess the phylogenetic relationships between SARS-CoV-2 viruses obtained from positive nasal swab samples. Phylogenetic trees will be generated to determine whether participants were part of a transmission cluster.

### Safety Analyses

Safety analyses will be conducted in the safety population.

The proportion of participants with Grade 3 AEs, Grade 4 AEs, AEs resulting in study intervention discontinuation, and SAEs will be summarized by treatment group, with 95% exact CIs.

In addition, reported AESIs will be summarized overall and by group.

## Interim Analysis

The primary analysis will occur after all participants complete Day 35. There will be 1 prespecified interim analysis to assess futility. If the trial does not cross the pre-specified boundaries to declare futility at the interim analysis, a final efficacy and safety analysis will occur after all participants complete the study Day 35 of follow-up.

A group sequential design strategy will be used to assess futility at the interim analysis. The interim analysis will occur after approximately 200 participants have completed Day 28 of follow-up. A Hwang-Shih-DeCani beta spending approach will be used [Jennison 2000]. The gamma parameter used will be -4, which is similar to the boundaries from the O’Brien-Fleming method [O’Brien 1979]. The lower bound for futility will be binding.

# Operational Considerations

## Independent Data Monitoring Committee

An IDMC will be convened for this study with expertise in COVID-19 or respiratory viruses and emerging epidemics as well as biostatistics.

The purpose of the IDMC is to monitor the study for safety and efficacy. The IDMC may request additional information, or a pause in recruitment and dosing, while safety data are being evaluated.

The IDMC will operate according to a charter and the structure, participants, meeting information and other details will be provided in the charter. The charter will be available prior to study start.

The IDMC will meet monthly or ad hoc, if necessary, to review safety data, and may request additional information, or a pause, while safety data are being evaluated.

The IDMC will also review unblinded efficacy data at a pre-specified interim analysis. Pausing guidelines will apply (refer to Section 6.6).

All procedures associated with this review will be documented.

The IDMC will make a formal recommendation as needed.

The recommendations of the IDMC, along with the Sponsor’s decision, will be communicated to the PIs and the IRBs/IECs and the national regulatory authorities as required. The Sponsor or its designee agrees to abide by any directives issued by the national regulatory authority or the IRB/IEC.

The IDMC will review the Pausing Guidelines (Section 6.6 Study Pausing Guidelines).

## Informed Consent Process

The PI or designee will explain the study to the participant and answer all questions regarding the study. The PI or designee will conduct the consent discussions on an individual basis with each participant. Adequate time will be allowed for all questions to be addressed. Potential participants will be interviewed to ensure that they meet all entry criteria relating to history.

Participants must be informed that their participation is voluntary. Participants will be required to sign a statement of informed consent that meets the requirements of FDA Code of Regulations (CFR) 21 CFR 50, local regulations, International Council for Harmonization of Technical Requirements for Pharmaceuticals for Human Use (ICH) guidelines, and the IRB/IEC or study center.

Written informed consent must obtained before the participant is enrolled in the study, and the date the written consent was obtained must be documented. The authorized person obtaining the informed consent must also sign the ICF (see Section 11.1.2.1).

A copy of the ICF must be provided to the participant.

The intention for this study is that the consenting process will be conducted through remote/virtual contact and may use electronic consent functionality to obtain the participant’s signature and date. Alternatively, an in-person visit may be conducted for consent if needed.

### Informed Consent Forms

The study ICF contains a section that addresses the use of remaining mandatory samples for research not described in the protocol, e.g., assay development and assay quality control.

Participants will be told that they are free to refuse to participate and may withdraw their consent at any time and for any reason during the storage period. Any test results from the samples collected before withdrawing consent may still be used for the study.

## Data Protection

Participants will be assigned a unique identifier by the Sponsor. Any participant record or dataset that is transferred to the Sponsor will contain the identifier only; participant names or any information which would make the participant identifiable will not be transferred to the Sponsor.

The participant must be informed that the participant’s study-related data will be used by the Sponsor in accordance with local data protection law. The level of data disclosure must also be explained to the participant.

The participant must be informed that the participant’s medical records may be examined by Clinical Quality Assurance auditors or other authorized personnel appointed by the Sponsor, by appropriate IRB/IEC members, and by inspectors from regulatory authorities.

## Sample Retention and Future Use of Collected Biospecimen

Biospecimens collected during the study will be retained securely for up to 10 years.

Collected biospecimens may also be used for future research. In addition, and only with the explicit and optional consent of study participants, samples or partial sample volumes that remain once a protocol-defined assay has been completed, may be stored and used for purposes other than protocol-related endpoints. Such “future research studies” would include, but not be limited to, assay development, assay quality control and development of methods related to COVID-19 research.

Biospecimens may be shared with research partners for scientific research purposes. Samples will not be sold, loaned, or given to any other independent groups for their own use. Research partners working with the Sponsor are not allowed to share samples with anyone who is not authorized by the Sponsor. The Sponsor will control what is done with all biospecimens.

## Dissemination of Clinical Study Data

Study information from this protocol will be posted on publicly available clinical trials registers (for example, clinicaltrials.gov) before enrollment of participants begins. Summaries of the results of the study will also be posted on the same website.

The final CSR will include all available data through the final study visit. The database will be locked prior to preparation of the final CSR when all of the above data have been entered, reviewed, and all queries related to the data have been addressed.

Modifications or additions to the analyses will be included in the relevant SAP. Any decisions to deviate from the planned analyses described in the protocol and in the SAP will be described in detail in the final CSR. The CSR will be reviewed and approved by the Sponsor signatory and the lead PI.

## Data Quality Assurance

All participant data relating to the study will be recorded on a printed CRF or, by an electronic CRF (eCRF) using an Electronic Data Capture (EDC) system or transmitted to the Sponsor or designee electronically (e.g., laboratory data). The PI is responsible for verifying that data entries are accurate and correct by physically signing or electronically signing the CRF.

The PI must maintain accurate documentation that supports the information entered in the CRF/eCRF.

The study will be monitored regularly by the Sponsor or its designee throughout the study period. The PI must permit study-related monitoring, audits, IRB/IEC review, and regulatory agency inspections and provide direct access to source data documents.

Monitoring details describing strategy (e.g., risk-based initiatives in operations and quality such as Risk Management and Mitigation Strategies and Analytical Risk-Based Monitoring), methods, responsibilities and requirements, including handling of noncompliance issues and monitoring techniques (central, remote, or on-site monitoring) are provided in the Monitoring Plan. Remote monitoring is preferred in this study.

The Sponsor or designee is responsible checking the accuracy and completeness of the data reported in the CRFs and the consistency with the source documents. The Sponsor assumes accountability for actions delegated to other individuals (e.g., contract research organization [CRO]).

Study monitors will perform ongoing source data verification remotely to confirm that data entered into the CRF by authorized site personnel are accurate, complete, and verifiable from electronic source documents provided; that the safety and rights of participants are being protected; and that the study is being conducted in accordance with the currently approved protocol and any other study agreements, ICH GCP, and all applicable regulatory requirements.

## Source Documents

Source documentation, either paper or electronic, consists of existing medical records and/or study records developed and maintained by the PI. Source documents provide evidence for the existence of the participant and substantiate the integrity of the data collected.

Data recorded on source documents will be transcribed onto CRFs either paper, or electronically using an EDC system.

Data entered in the CRF that are transcribed from source documents must be consistent with the source documents or the discrepancies must be explained. The PI may need to request previous medical records or transfer records, depending on the study.

## Record Retention and Future Use of Medical Data

Records and documents pertaining to the conduct of this study must be retained by the PI for a minimum of 10 years after study completion unless local regulations or institutional policies require a longer retention period.

No records may be destroyed during the retention period without the written approval of the Sponsor. No records may be transferred to another location or party without written notification to the Sponsor.

In addition, medical data collected from this study may be used for future use and may be shared with research partners for scientific research purposes. The information may be analyzed, used, or combined with other results, information, and data from other studies for the purpose of this study, in research registries or databases, for future research purposes that may or may not be related to this study for scientific publications or presentations, for submission to governmental authorities, for legal or regulatory purposes, or to seek approval of a study drug or study treatment for marketing from governmental authorities.  If the results of the study are published, your identity will remain confidential.

## Study Closure

The Sponsor reserves the right to close the study or terminate the study at any time for any reason at the sole discretion of the Sponsor.

Reasons for the early closure of a study site by the Sponsor may include but are not limited to:

- Failure of the PI to comply with the protocol, the requirements of the IRB/IEC or local health authorities, the Sponsor's procedures, or GCP guidelines
- Inadequate recruitment of participants by the PI
- Discontinuation of further study intervention development.

## Publication Policy

The results of this study may be published or presented at scientific meetings. If this is foreseen, the PI agrees to submit all manuscripts or abstracts to the Sponsor before submission. This allows the Sponsor to protect proprietary information and to provide comments.

The Sponsor will comply with the requirements for publication of study results. In accordance with standard editorial and ethical practice, the Sponsor will generally support publication of multicenter studies only in their entirety and not as individual site data. In this case, a coordinating PI will be designated by mutual agreement.

Authorship will be determined by mutual agreement and in line with International Committee of Medical Journal Editors authorship requirements.

# References

Ackermann M. Verleden S, Kuehnel M. Pulmonary vascular endothelialitis, thrombosis, and angiogenesis in COVID-19. NEJM. 2020. DOI: 10.1056/NEJMoa2015432

Bikdeli B, Madhavan M, Jimenez D, et al. J Am Col Cardio. 2020;75(3):2950-73. COVID-19 and thrombotic or thromboembolic disease: implications for prevention, antithrombotic therapy, and follow-Up. https://dio.org/10.1016/jacc.2020.04.031.

Beigel H, Tomashek K, Dodd L. et al. Remdesivir for the treatment of COVID-19 - Preliminary Report. NEJM. 2020 May 22: DOI: 10.1056/NEJMoa2007764

Cai J, Xu J, Lin D, et al. A case series of children with 2019 novel coronavirus infection: clinical and epidemiological features. Clin Infect Dis. 2020 Feb. pii: ciaa198.

Chen N, Zhou M, Dong X, et al. Epidemiological and clinical characteristics of 99 cases of 2019 novel coronavirus pneumonia in Wuhan, China: a descriptive study. Lancet. 2020 Feb;395(10223):507-513.

Ciceri F, Castagna A, Rovee-Querini P, De Cobelli F, et al. Early predictors of clinical outcomes of COVID-19 outbreak in Milan, Italy. Clin Immunol 2020;217:108509 https://doi.org/10.1016/j.clim.2020.108509

Cohen A, Spiro T, Büller H, et al. Rivaroxaban for Thromboprophylaxis in Acutely Ill Medical Patients. N Engl J Med. 2013;368:513-523. DOI: 10.1056/NEJMoa1111096.

Division of AIDS, National Institute of Allergy and Infectious Diseases, National Institutes of Health US Department of Health and Human Services (DAIDS) Regulatory Support Center. 2017. Table for grading the severity of adult and pediatric adverse events. Corrected version 2.1. <https://rsc.tech-res.com/docs/default-source/safety/division-of-aids-(daids)-table-for-grading-the-severity-of-adult-and-pediatric-adverse-events-corrected-v-2-1.pdf>.

FDA Guidance on Conduct of Clinical Trials of Medical Products during COVID-19 Public Health Emergency Guidance for Industry, PIs, and Institutional Review Boards March 2020. Updated on May 14, 2020. <https://www.fda.gov/media/136238/download>.

FDA. Letter of Authorization for Emergency Use of remdesivir for the treatment of COVID-19. May 1, 2020. <https://www.fda.gov/media/137564/download>.

Fu J. Kong J, Wang W, et al. The clinical implication of dynamic neutrophil to lymphocyte ratio and D-dimer in COVID-19: A retrospective study in Suzhou China. Thromb Res. 2020;192:3-8. [doi.org/10.1016/j.thromres.2020.05.006](https://doi.org/10.1016/j.thromres.2020.05.006).

Hoffmann M, Kleine-Weber H, Schroeder S, et al. SARS-CoV-2 cell entry depends on ACE2 and TMPRSS2 and is blocked by a clinically proven protease inhibitor. 2020 Mar; Cell. pii: S0092-8674(20)30229-4 (pre-publication).

Huang C, Wang Y, Li X, et al. Clinical features of participants infected with 2019 novel coronavirus in Wuhan, China. Lancet. 2020 Feb;395(10223):497-506.

Jennison C, Turnbull BW. Group sequential methods with applications to clinical trials. 2000. Boca Raton: Chapman and Hall.

Johns Hopkins University (JHU) and Medicine. 2020. Coronavirus resource center. [https://coronavirus.jhu.edu/us-map. Accessed 02 September 2020](https://coronavirus.jhu.edu/us-map.%20Accessed%2002%20September%202020).

Lee JS, Adhikari NKJ, Kwon HY, et al. [Anti-ebola therapy for participants with Ebola virus disease: a systematic review.](https://www.ncbi.nlm.nih.gov/pubmed/31046707) BMC Infect Dis. 2019 May;19(1):376.

Li CC, Wang XJ, Wang HR. Repurposing host-based therapeutics to control coronavirus and influenza virus. Drug Discov Today. 2019 Mar;24(3):726-736.

Linton NM, Kobayashi T, Yang Y, et al. Incubation period and other epidemiological characteristics of 2019 novel coronavirus infections with right truncation: a statistical analysis of publicly available case data. J Clin Med. 2020 Feb;9(2). pii: E538.

McGonagle D, O’Donnell J, Sharif K, et al. Immune mechanisms of pulmonary intravascular coagulopathy in COVID-19 pneumonia. Lancet Rheumatol. 2020 Published Online May 7, 2020. https://doi.org/10.1016/S2665-9913(20)30121-1.

[Miettinen](https://onlinelibrary.wiley.com/action/doSearch?ContribAuthorStored=Miettinen%2C+Olli) O, [Nurminen](https://onlinelibrary.wiley.com/action/doSearch?ContribAuthorStored=Nurminen%2C+Markku) M. Comparative analysis of two rates.1985;4(2):213-226. <https://doi.org/10.1002/sim.4780040211>.

Micco P, Russo V, Carannante N, et al. Clotting Factors in COVID-19: Epidemiological association and prognostic values in different clinical presentations in an Italian cohort. J. Clin. Med. 2020;9:1371. DOI:10.3390/jcm9051371.

Nauka P, Oran E, Chekuri S. Deep venous thrombosis in a non-critically ill patient with novel COVID-19 infection. Thromb Res. 2020;192:27-8. <https://www.thrombosisresearch.com/article/S0049-3848(20)30187-0/pdf>.

O’Brien, PC, Fleming, TR. A Multiple Testing Procedure for Clinical Trials. Biometrics. 1979;35:549–56.

Petrilli C, Jones SA, Yang J, et al. Factors associated with hospital admission and critical illness among 5279 people with coronavirus disease 2019 in New York City: prospective cohort study. BMJ 2020;369:m1966.

Richardson S, Hirsch J, Narasimhan, M, et al. Presenting characteristics, comorbidities, and outcomes among 5700 patients hospitalized with COVID-19 in the New York City Area. JAMA. 2020;323(20):2052-2059.

Siddiqi H, Mehra MR. COVID-19 illness in native and immunosuppressed states: A clinical−therapeutic staging proposal. J Heart and Lung Trans. 2020. https://doi.org/10.1016/j.healun.2020.03.012.

Tang N, Bai H, Chen X, et al. Anticoagulant treatment is associated with decreased mortality in severe coronavirus disease 2019 patients with coagulopathy. J Thromb Haemost. 2020;18:1094–1099. <https://doi.org/10.1111/jth.14817>.

University of Washington (UW) Medicine Pharmacy Services. Anticoagulation Services. Rivaroxaban drug interaction potential. Modified on 23 April 2020. <https://depts.washington.edu/anticoag/home/content/rivaroxaban-drug-interaction-potential>.

US Center for Disease Control and Prevention (CDC). 2020. Coronavirus disease 2019. www.cdc.gov Accessed 26 May 2020.

Wang D, Hu, Bo, Hu Chang, et al. Clinical characteristics of 138 hospitalized patients with 2019 novel coronavirus–infected pneumonia in Wuhan, China. JAMA. 2020;323(11):1061-1069. doi:10.1001/jama.2020.1585. Published online February 7, 2020. Corrected on February 20, 2020.

Wang T, Chen R, Liu C. Attention should be paid to venous thromboembolism prophylaxis in the management of COVID-19. www.thelancet.com/haematology Vol 7 May 2020 https://doi.org/10.1016/ S2352-3026(20)30109-5.

White House Press Release (WHPR). [https://www.whitehouse.gov/presidential-actions/proclamation-declaring-national-emergency-concerning-novel-coronavirus-disease-covid-19-outbreak. 2020 Mar 13](https://www.whitehouse.gov/presidential-actions/proclamation-declaring-national-emergency-concerning-novel-coronavirus-disease-covid-19-outbreak.%202020 Mar%2013).

Wichmann D, Sperhake J-P, Lütgehetmann M, et al. Autopsy findings and venous thromboembolism in patients with COVID-19. Ann Intern Med. 2003. doi:10.7326/M20-2003.

Williams RD, Markus AF, Yang C, et al. Seek Cover: Development and validation of a personalized risk calculator for COVID-19 outcomes in an international network. medRxiv May 29, 2020. [https://doi.org/10.1101/2020.05.26.20112649](https://doi.org/10.1101/2020.05.26.20112649di).

World Health Organization (WHOa). News Release. Statement on the second meeting of the International Health Regulations (2005) Emergency Committee regarding the outbreak of novel coronavirus (2019-nCoV). 2020 Jan 30.

WHO(b) News Release. Shortage of personal protective equipment endangering health workers worldwide. 2020 Mar 03.

WHO(c). Clinical management of severe acute respiratory infection when novel coronavirus (‎‎‎‎‎‎2019-nCoV)‎‎‎‎‎‎ infection is suspected: interim guidance, 28 January 2020. World Health Organization. <https://apps.who.int/iris/handle/10665/330893>.

WHO(d) Working Group on the Clinical Characterization and Management of COVID-19 infection. A minimal common outcome measure set for COVID-19 clinical research. Lancet Infect Dis 2020. Published Online June 12, 2020 https://doi.org/10.1016/S1473-3099(20)30483-7.

Wu C, Chen X, Cai Y, et al. Risk factors associated with acute respiratory distress syndrome and death in patients with coronavirus disease 2019 pneumonia in Wuhan, China. JAMA Intern Med. doi:10.1001/jamainternmed.2020.0994. Published online March 13, 2020. Corrected on May 11, 2020.

Xarelto® [rivaroxaban] tablets, for oral use, Package Insert, revised Mar 2020

Zhou F, Yu Ting, Du Ronghui, et al. Clinical course and risk factors for mortality of adult inpatients with COVID-19 in Wuhan, China: a retrospective cohort study. Lancet 2020;395:1054–62.

Zhu N, Zhang D, Wang W, et al. A novel coronavirus from participants with pneumonia in China, 2019. N Engl J Med 2020 Feb;382(8):727-7.

# Version History

Version 1 (30 June 2020) and Version 2 (02 July 2020) were in-house versions shared with CRO for reviews and prepared prior to FDA review.

Version 3 (15 July 2020) was submitted to FDA for review.

Version 4 (01 August 2020) incorporated FDA suggestions and is the version used for initiation of the study.

Amendment 1 (09 September 2020) is the first amendment to the protocol after study start.

Amendment 2 (27 January 2021) is the second amendment to the protocol after study start.

## Amendment 1 dated 09 Sept 2020

**Rationale for Amendment 1:** The protocol required an amendment after study start to address emergent and important issues at sites pertaining to screening and enrollment. The requirement for screening to occur within 72 hours of symptom onset resulted in many potential participants who had symptoms onset before 72 hours being excluded from consideration for enrollment. In addition, the requirement for Day 1 to occur no later than 72 hours after screening did not take into account the time required to obtain SARS-CoV-2 test results (for participants not having documentation of a prior positive test) nor the time needed to provide study materials to participants once randomization had occurred. The time constraints described above restricted enrollment severely and the study team decided that both constraints needed to be relaxed to enable efficient enrollment.

Therefore, changes to the Inclusion criteria #3 and #4 (Section 5.5.1) are:

1. Documented positive SARS-CoV-2 diagnostic testing with a sample collected **≤10** days of screening.
2. Symptomatic for COVID-19 for **≤7** days at time of **randomization**

(symptomatic is defined as having at least **one** of the following symptoms of COVID-19 that is of new onset or has worsened from baseline***:*** ~~and include~~ fever, chills, myalgia, arthralgia, headache, fatigue, cough, sore throat, nasal congestion, nausea, vomiting, or diarrhea***)***.

The change to Section 8.1 is:

- Required procedures must occur ≤**5 days** ***prior to*** the Day 1 visit.

Because the putative effect of rivaroxaban to prevent thrombotic complications of COVID-19 would be present during the whole of the treatment period, the expansion of the intervals as detailed above are not anticipated to significantly impact the results of the study.

**A complete list of changes from protocol Version 4 to protocol Amendment 1 are shown as follows.**

Title page: Added text: Refer to Section 13 for version history and amendment revisions.

Changed header throughout document from Version 4 to Amendment 1.

Changed footer throughout document from 01 August to 09 September

Changed COVID-19 disease to COVID-19 throughout

Sponsor signatory page: added: Amendment 1

List of abbreviations: added: CRO contract research organization

Section 1 Synopsis and Section 5.1: Added under Interventional groups, randomization, and dosing: ***Randomization will be stratified by site and by number of days since onset of symptoms*** (***<6 days vs. ≥6 days).***

Synopsis: Figure 1: Study Screening Process

- Positive SARS-CoV-2 ***diagnostic testing*** ~~≤ 7 days at screening~~ ***performed ≤10 days*** prior to screening
- Mild COVID-19 symptoms ~~≤ 72 hours at screening~~ ***present ≤7 days at the time of randomization*** ~~≤ 72 hours~~
- ~~≤ 72 hours~~ ***≤5 days*** from screening to Day 1
- ~~Have mild COVID-19 symptoms~~

If eligibility is confirmed, enroll and randomize ~~(intervention or control) at screening~~.

Section 2. Schedule of Activities:

Changed screening from ~~≤ 72 hours~~ to ***≤5 days***

Deleted X in the Day 35 column to remove clinical status assessment using ordinal scales for Gates MRI and WHO from this time point.

Added: Self-collection of nasal ***or oropharynx*** SARS-CoV-2 sample for diagnostic testing^3^

Added an (X)^1^ under screening for Self-collection of nasal SARS-CoV-2 sample for diagnostic testing

SOA footnotes:

^1^~~If there is documentation of results of a positive diagnostic testing within the past 7 days, this test does not need to be repeated~~ ***If there is documented positive SARS-CoV-2 diagnostic testing performed with a sample collected ≤10 days prior to screening, the test does not need to be performed. If no documentation, the potential participant will be asked to collect a nasal or oropharynx swab sample to send for testing.***

***^2^If documentation of a previous positive SARS-CoV-2 test is not available and a test is performed at screening, randomization will occur after a positive result is obtained.***

***^~~2~~^* ^3^** ***Participant will be asked to collect a sample per kit instructions.*** Discontinue collection during hospitalization and/or, ***per physician’s discretion,*** when two consecutive negative results are available at any time during the study.

Section 3.1.1 SARS-CoV-2 Epidemiology and Disease, changed: As of ~~June~~ ***September*** 2020, approximately ***26*** ~~10~~ million confirmed cases were reported globally, with over ~~500~~ ***859***,000 deaths JHU 2020].

Section 3.1.2. Therapeutic Approaches for COVID-19, changed: As of ~~June~~***September*** 2020

Section 3.2.2 COVID-19 progression requiring hospitalization generally occurs from the ***second week*** of illness ….

Section 3.2. Table 2 moved text in title to footnote: Safety Analysis Set- On Treatment Plus 2 Days

Section 3.2.2.1 new section added: Rationale for a Limit of 7 Days of Mild COVID-19 Symptoms Prior to Randomization

The incubation period from exposure to early manifestations of COVID-19 ranges from 2 to 14 days. During the first 3 days of illness, COVID-19 symptoms may be non-specific and include cough, fever, headache, lethargy, and/or diarrhea. During the next 3-5 days some patients develop worsened symptoms but some patients may show signs of recovery. The second week of illness, Days 8-12, is when progression to signs and symptoms indicative of a hyperinflammatory phase is more likely to occur. These may include dyspnea, abnormal imaging of the lungs, respiratory failure, ARDS, and/or cytokine storm syndrome. In a large series of 410 patients with COVID-19 hospitalized in Italy [Ciceri 2020], the median (IQR) days from COVID-19 symptoms onset to hospitalization was 8 days (5-11) days. Approximately 50% of these patients were aged >65 years and approximately 60% had one or more comorbidities.

The primary endpoint of this study is the time to progression of COVID-19 from Scale 2 to Scale 3 or higher – a situation that would likely require hospitalization of the participant. The data from the cited Italian study can inform the choice of the interval between onset of symptoms and the expected time of initiation of study drug therapy. Because the interval from onset of symptoms to randomization should be no more than 7 days resulting in the worst-case scenario of an interval of around 9 days from onset of symptoms to first dose of study treatment (assuming two days for shipment of IP and supplies). Nine days falls within the IQR reported by Ciceri, et al [Ciceri 2020].

Section 4 Objectives and Endpoints Table 3:

- Added ***Objectives and*** to the title of Table 3. Modified first secondary endpoint: Time to disease resolution, ***defined as BOTH viral clearance*** (two consecutive negative diagnostic tests) and symptoms resolution (new onset COVID-19 symptoms resolved, and pre-existing symptoms returned to baseline*) ~~with viral clearance~~ through Day 28

Section 5.4. Pre-Screening for the Study

~~Persons with positive SARS-CoV-2 diagnostic test who have not exhibited symptoms and are at high risk for COVID-19 disease progression may enroll in the pre-screening phase provided that they are willing to be contacted by the study team and are interested in enrolling in the COVID-19 treatment trial if they become symptomatic.~~

***The purpose of pre-screening is to facilitate enrollment of participants with mild COVID-19 into the study as soon as they become symptomatic. Persons who are in the high risk category for COVID-19 progression and are asymptomatic may enroll in the pre-screening phase provided that they are willing to be contacted by the study team and are interested in enrolling in the COVID-19 treatment trial if they become symptomatic. They must have a confirmed positive SARS-CoV-2 diagnostic testing within the past 7 days at Day 1 of pre-screening.***

***Participants who do not yet have a documented positive SARS-CoV-2 diagnostic test may have the testing done at the pre-screening eligibility check visit provided that they have had a high risk exposure to SARS-CoV-2 within the past 14 days.***

***High risk exposure is defined as:***

1. ***being an immediate household contact of a confirmed COVID-19 case and/or***
2. ***having occupational exposure to a confirmed COVID-19 case (for example, healthcare worker or first responder).***

Section 5.4 Figure 2

Fulfill key eligibility criteria

- Age ≥18 yrs old
- At high risk for severe COVID-19 ~~disease~~
- Positive SARS-CoV-2 test ≤~~5~~***7*** days ***at day 1 of pre-screening***
- No COVID-19 symptoms
- ***Laboratory tests obtained and reviewed***
- Willing to be contacted by study team
- Interested in participating in study

Box below: Day 1 to ~~Day 7~~ ***Day 10***

Last box: Exit pre-screening if no symptoms at Day 10 after diagnosis or Day 10 of pre-screening, ~~if no symptoms at Day 7 after diagnosis or Day 7 of pre-screening,~~ whichever comes first**.**

Section 5.4.1. Pre-Screening Inclusion Criteria

COVID-19 disease characteristics

3. ~~Documented SARS-CoV-2 positive diagnostic test of ≤5 days at time of informed consent~~

***Documented positive SARS-CoV-2 diagnostic testing with sample collected ≤7 days at Day 1 of pre-screening***

No COVID-19 symptoms (***defined as*** ~~no~~ fever, chills, myalgia, arthralgia, headache,…

Section 5.4.2, Table 4: Pre-screening Schedule of Activities

Eligibility check

(≤~~24~~ ***72*** hrs)

Added a row at end of table

| ***No AEs will be collected*** |
| --- |

***Added: Note that no*** data during the pre-screening stage will be recorded in the database or in a case report form.

**Footnote:**

^~~1~~^~~If there is documentation of results within the past 5 days of a positive diagnostic test, this test does not need to be repeated.~~

^~~2~~^~~Participant will exit the pre-screening phase if he/she remains asymptomatic at day 7 after diagnosis or day 7 of the pre-screening phase, whichever comes first.~~

***^1^If documented positive SARS-CoV-2 testing is not available at the eligibility check visit, testing may be performed provided that the potential participant has had a high risk exposure to SARS-CoV-2, defined as being an immediate household contact of a confirmed COVID-19 case and/or having occupational exposure to a confirmed COVID-19 case (for example, healthcare worker or first responder).***

***^2^Participant will exit the pre-screening phase if he/she remains asymptomatic at day 10 after diagnosis or day 10 of the pre-screening phase, whichever comes first.***

Section 5.5.1 Inclusion and Exclusion Criteria

COVID-19 Characteristics

1. Documented positive SARS-CoV-2 diagnostic testing with a sample collected **≤10** days of screening.
2. Symptomatic for COVID-19 for **≤7**~~2 hours~~ days at time of ~~screening~~ **randomization**

(symptomatic is defined as having at least ~~two~~ ***one*** of the following symptoms of COVID-19 that is of new onset or has worsened from baseline, and include fever, chills, myalgia, arthralgia, headache, fatigue, cough, sore throat, nasal congestion, ~~anosmia, ageusia~~, nausea, vomiting, or diarrhea.

~~Must have at least one symptom other than~~ **~~anosmia or ageusia~~**~~.~~

Section 5.5.2 Exclusion Criteria under Medical Conditions or history

3. Hypoxemia (oxygen saturation<94% in ambient air or oxygen saturation below pre-COVID-19 ~~level~~ ***oxygen saturation [if known]*** for people with known COPD) at Day 1

**6.11. Hospitalization**

If a participant clinically worsens, such as requiring hospitalization, it is expected that standard clinical care will be provided. ~~and participation in another study intervention trial will be permitted~~. ***Hospitalized participants may continue to receive study intervention if maintained on the originally randomized study-intervention regimen.***

~~The treating physician will be contacted by the study investigator or designee, to get approval for continuing study encounters to the extent possible, in the hospital setting.~~

Section 7.1 Discontinuation of Study Intervention:

Study intervention will be discontinued for the following reasons:

~~Hospitalization, at the discretion of the in-patient provider: hospitalized participants may continue to receive study intervention if maintained on the originally randomized Study Intervention regimen)~~ ***Hospitalization, if the in-patient provider decides continuation of study treatment is contraindicated.***

Section 7.2 Withdrawal from the Study: Added bullet:***The participant progressed to moderate or severe COVID-19 (or worse) prior to initiation of IP dosing on Day 1***

Section 8 Study Encounters: edited: Participants will be instructed to seek clinical care should they manifest any signs or symptoms of COVID-19 ~~disease~~ progression requiring medical intervention and notify ~~their~~ ***the treating*** physician about trial participation.

Section 8.1 Screening Process and Procedures: edited:

The following procedures must occur ≤***5 days prior to*** ~~72 hours of the~~ Day 1 visit.

The study staff conducting the ***Screening*** visit will:

~~Collect laboratory documentation of positive SARS-CoV-2 diagnostic test performed ≤7 days of screening~~. ***Collect documented positive SARS-CoV-2 diagnostic testing performed with a sample collected ≤10 days of screening.*** If no ***~~such~~*** document is available, SARS-CoV-2 diagnostic testing will be performed at screening.

~~Enrolled eligible participants and randomized to a study group.~~

~~Collect information on AEs in eligible participants.~~

~~If eligible, participants will receive supplies and study IP, delivered to the participant at home, or if deemed necessarily it may be picked up at study site or other methods may be used as appropriate.~~

***Eligible participants who meet all criteria will be enrolled and randomized to a study group and will receive supplies and study IP, delivered to the participant at home, or if deemed necessary, it may be picked up at study site or other methods may be used as appropriate.***

Eligible participants will receive the following:

- ~~Nasal~~ ***Diagnostic*** test kit, with user instructions

Section 9.1: Day 1 (first day of study)

- Assess the Gates MRI and WHO ordinal scales ***and confirm that participant continues to meet criteria for Scale 2 of Gates MRI COVID-19 Ordinal Scale*** ***(Table 7)***
- Collect information on AEs (***note that AEs will be collected from the time the participant signs the informed consent).***

Deleted bullet: ~~Collect information on bleeding events and severity~~

Section 9.4 Efficacy Assessments

Edited: ~~The primary efficacy endpoint is the proportion of participants who progress to moderate or severe disease category or higher (Gates MRI ordinal scale ≥3).~~

***The proportion of participants who progress to moderate or severe category or higher (Gates MRI ordinal scale ≥3) will be assessed at study visits indicated in the SoA.***

Section 9.4.1 Nasal Sampling: edited: ~~Nasal sampling for SARS-CoV-2 diagnostic test will follow study instruction document from the testing laboratory. Nasal sampling collection will be discontinued during hospitalization and/or when two consecutive negative results are available at any time during the study.~~

***Nasal sampling for SARS-CoV-2 diagnostic testing will follow study instruction document from the testing laboratory. Nasal sampling collection will be discontinued during hospitalization and/or, per physician’s discretion, when two consecutive negative results are available at any time during the study.***

Section 9.6 Table 6 title: ***COVID-19*** Signs and Symptoms to be Recorded in the CRF/eCRF

Section 10.2 Primary Hypothesis: ~~Prophylactic treatment~~ ***Treatment*** with

Section 10.5: ***All AEs will be followed until resolution, stabilization, lost to follow-up, or until the end of the study (Day 35).*** ~~All SAEs will be followed until resolution, stabilization, or the participant is lost to follow-up, until the end of the study (Day 35).~~

Section 10.5.5. ~~All SAEs, regardless of whether related or not to the IP, will be followed until resolution, stabilization, or the participant is lost to follow-up, until the end of the study (Day 35).~~***All SAEs, regardless of whether related or not to the IP, will be followed until resolution, stabilization, lost to follow-up, or until the end of the study (Day 35).***

Section 12 References: added Ciceri F, Castagna A, Rovee-Querini P, De Cobelli F, et al. Early predictors of clinical outcomes of COVID-19 outbreak in Milan, Italy. Clin Immunol 2020;217:108509 https://doi.org/10.1016/j.clim.2020.108509

Minor typographical errors and formatting errors were corrected throughout the document.

## Amendment 2 dated 27 January 2021

**Rationale for Amendment 2**

The pace and trajectory of study enrollment suggests that the targeted number of total study participants (600) will be enrolled by the time of the proposed data cut for the second interim analysis that was previously planned. The majority of study participants would have completed the study by the time the IDMC meeting would take place. Therefore, any decision stemming from the proposed second interim analysis would unlikely affect the study conduct.

Therefore, based on these circumstances the protocol is amended to reduce the number of interim analyses from 3 to 1 interim analysis and to remove the sample size re-estimation. Additional changes included modify the first interim analysis from an evaluation of futility and efficacy to be only for futility, and to remove any additional pre-specified interim analyses.

A complete list of changes is provided below.

**List of Changes**

Header: Changed from Amendment 1 to Amendment 2

Footer: Changed from 09 September 2020 to 27 Jan January 2021.

Title page: **Approval Date:** Version 4, Amendment ***2*** ~~1~~, ***27 January 2021*** ~~09 September 2020~~

Signature page: Changed from Amendment 1 to Amendment 2

**Synopsis**

**Safety and efficacy monitoring**: The IDMC will also review unblinded efficacy data at ***a*** pre-specified interim ***analysis*** ~~analyses.~~

**Analysis:** The primary analysis will occur after all participants complete Day 35. ***There will be 1 interim analysis to assess futility. The interim analysis will occur after the first approximately 200 participants complete Day 28.***

~~There will be up to 3 interim analyses to assess the appropriateness of the planned sample size and efficacy. The first interim analysis will occur after the first approximately 200 participants complete Day 28. At the first interim analysis, a sample size re-assessment will be performed. If the final sample size is not adjusted, the first interim analysis for efficacy will also occur at this time and the second interim analysis will occur when approximately 400 participants complete Day 28. If the final sample size is adjusted at the first interim analysis, the first and second interim analyses for efficacy will occur when approximately one-third and two-thirds of the adjusted total number of participants complete Day 28, respectively.~~

If the trial does not cross the pre-specified boundaries to declare early ~~efficacy or~~ futility at the interim ***analysis*** ~~analyses~~, a final efficacy and safety analysis will occur after all participants complete Day 35.

**Section 10.1** **Sample Size Determination**

Efficacy

The total number of participants planned to be enrolled is approximately 600 (300 per group). If the true control rate for progression to moderate or severe category or greater is at least 30%, 294 participants per group will result in 80% power when the true Study Intervention effect is 35%. This sample size assumes a drop-out rate of 10% and a 1-sided Type I error rate of 2.5%.

~~To account for two planned interim efficacy analyses without adversely affecting the statistical power, the sample size was increased to 300 per group.~~

~~Because the statistical power relies heavily on the observed control event rate, the final target sample size will be assessed using the observed control event rate and overall drop-out rate as a blinded sample size reassessment after approximately 200 participants have completed Day 28 of follow-up (see Section 10.6 for interim analyses).~~

**Section 10.3.1 Assigning Shared Controls**

- The primary comparison will utilize eligible participants in the ~~SOC~~ ***control*** arm with all control formulations.

**Section 10.5 Statistical Methods**

The statistical analysis plan will be developed and finalized before database lock and will describe the detailed analytical plans and procedures for ***the*** interim and final analyses***,*** ~~performing sample size reassessments,~~ ~~interim and final analyse~~s, and accounting for missing data.

**Section 10.6 Interim *Analysis* ~~Analyses~~**

There will be ***1*** ~~up to 3~~ prespecified interim ~~analyses~~ analysis to assess ***futility*** ~~the appropriateness of the planned sample size and efficacy~~. If the trial does not cross the pre-specified boundaries to declare ~~early efficacy or~~ futility at the interim ***analysis*** ~~analyses~~, a final efficacy and safety analysis will occur after all participants complete the study Day 35 of follow-up.

A group sequential design strategy will be used to assess ***futility*** ~~efficacy at each~~ at ***the*** interim analysis. The ~~first and second analyses~~ interim ***analysis*** will occur after approximately 200 ~~and 400~~ participants have completed Day 28 of follow-up.

~~The first interim analysis will occur after the first approximately 200 participants complete Day 28. At the first interim analysis, a sample size re-assessment will be performed. The procedure for this blinded sample size reassessment will be detailed in the statistical analysis plan. In brief, the unblinded study statistician will assess the control event rate and drop-out rate, and recalculate the sample size required to detect treatment effects of 35%, 40%, 45% and 50% with 80% statistical power using a 2.5% one-sided type I error rate. Based on this assessment, the IDMC will make recommendations to the Sponsor for the final target sample size. This will be done with proper firewalls to maintain blinding of the information as it relates to the final target sample size.~~

~~If the final sample size is not adjusted, the first interim analysis for efficacy will also occur at this time and the second interim analysis will occur when approximately 400 participants complete Day 28. If the final sample size is adjusted at the first interim analysis, the first and second interim analyses for efficacy will occur when approximately one-third and two-thirds of the adjusted total number of participants complete Day 28.~~

**Section 11.1 Independent Data Monitoring Committee**

The purpose of the IDMC is to monitor the study for safety and efficacy. ~~The IDMC will also review any sample size re-assessment analyses and recommend sample size changes as appropriate.~~

The IDMC will also review unblinded efficacy data at ***a*** pre-specified interim ***analysis*** ~~analyses~~.
