# Supplementary material for: Randomized study of rivaroxaban vs. placebo on disease progression and symptoms resolution in high-risk adults with mild COVID-19
Source: Clin Infect Dis. 2021 Sep 15:ciab813. doi: 10.1093/cid/ciab813 (PMC8522357; doi:10.1093/cid/ciab813)
Supplement: Supplementary file 1 [file ciab813_suppl_Supplementary_Table_S3.docx]

**Supplemental Table 3: WHO Ordinal Scale for Assessment of Clinical Status of COVID-19 Patients**

| **Participant Status** | **Descriptor** | **Score** |
| --- | --- | --- |
| Uninfected | Uninfected; no viral RNA detected | 0 |
| Ambulatory mild disease | Asymptomatic; viral RNA detected | 1 |
|  | Symptomatic; independent | 2 |
|  | Symptomatic; assistance needed | 3 |
| Hospitalized moderate disease | Hospitalized; no oxygen therapy ^a^ | 4 |
|  | Hospitalized; oxygen by mask or nasal prongs | 5 |
| Hospitalized severe disease | Hospitalized; oxygen by NIV or high flow | 6 |
|  | Intubation and mechanical ventilation, pO2/FiO2 ≥150 or SpO2/FiO2 ≥200 | 7 |
|  | Mechanical ventilation pO2/FiO2 <150 (SpO2/FiO2 <200) or vasopressors | 8 |
|  | Mechanical ventilation pO2/FiO2 <150 and vasopressors, dialysis, or ECMO | 9 |
| Dead | Dead | 10 |

WHO clinical progression scale, WHO Working group [24]

^a ^If hospitalized for isolation only, record status as for ambulatory patient

NIV=non-invasive ventilation			

pO2=partial pressure of oxygen 

FiO2=fraction of inspired oxygen

SpO2=oxygen saturation

ECMO=extracorporeal membrane oxygenation
